# Auto-oxidation of *Ent*-beyer-15-en-19-al isolated from the essential oil of the heartwood of *Erythroxylum monogynum* Roxb.: formation of 15,16-epoxy-*ent*-beyeran-19-oic acid and other products

**DOI:** 10.1186/s13065-020-00671-9

**Published:** 2020-03-13

**Authors:** T. M. Samantha Gome Tennakoon, G. M. Kamal Bandara Gunaherath, K. Tuley Dayananda De Silva, Chayanika Padumadasa, D. Siril Abeywickrama Wijesundara, Ajita Mahendra Abeysekera

**Affiliations:** 1 Research and Development Laboratory, Link Natural Products Pvt. Ltd. Malinda, Kapugoda, Sri Lanka; 2 Department of Chemistry, Open University of Sri Lanka, P. O. Box 21, Nugegoda, Sri Lanka; 3 Department of Chemistry, University of Sri Jayewardenepura, Nugegoda, Sri Lanka; 4 National Institute of Fundamental Studies, Hantane, Kandy, Sri Lanka

**Keywords:** Erythroxylaceae, *Erythroxylum monogynum*, Essential oil, Auto-oxidation, Diterpenoids, Epoxy bayeranes, Hydroperoxides, Axial aldehyde group, 1D and 2D NMR

## Abstract

Chemical investigation of the essential oil obtained from the heartwood of *Erythroxylum monogynum* Roxb. yielded three beyerene type diterpenoids *ent*-beyer-15-ene (**1**), *ent*-beyer-15-en-19-ol (erythroxylol A) (**2**) and *ent*-beyer-15-en-19-al (**3**). *Ent*-beyer-15-en-19-al (**3**) was found to be unstable at room temperature, giving rise to hitherto unknown 15,16-epoxy-*ent*-beyeran-19-oic acid (**4**). This conversion involves the auto-oxidation of a C-4 axial aldehyde group of an *ent*-beyer-15-ene diterpenoid with the concurrent epoxidation of the C-15 double bond. This is the first report of the auto-oxidation of an aldehyde group to a carboxylic acid group with the concurrent epoxidation of a double bond in the same compound. Further investigation of this observation under controlled conditions resulted in the isolation and identification of *ent*-beyer-15-en-19-oic acid (**5**), two new epoxy hydroperoxides, 15,16-epoxy-19-*nor*-*ent*-beyeran-4*α*-hydroperoxide (**6a**), 15,16-epoxy-18-*nor*-*ent*-beyeran-4*β*-hydroperoxide (**6b**), and two new hydroperoxides, *ent*-beyer-19-*nor*-15-en-4*α*-hydroperoxide (**7**), *ent*-beyer-18-*nor*-15-en-4*β*-hydroperoxide (**8**) and *ent*-beyer-18-*nor*-15-en-4*β*-ol (**9**). Identification of these compounds was carried out by the extensive usage of spectroscopic data including 1D and 2D NMR. The acid **5** and the alcohol **9** have been reported previously as natural products from *Elaeoselinum asclepium* and *Erythroxylum monogynum*. The mechanistic basis of this auto-oxidation reaction is discussed. 
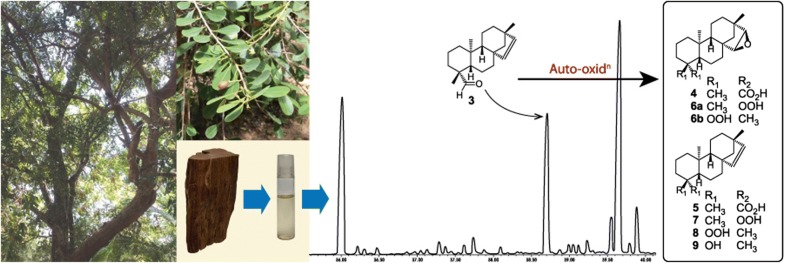

## Introduction

*Erythroxylum monogynum* Roxb. (Erythroxylaceae), is a small evergreen tree indigenous to Sri Lanka and India. The oil obtained by the distillation of its heartwood has been used traditionally as a wood preservative [1] and in the local perfumery industry. Many diterpenoids belonging to the kaurane and beyerane groups have been reported from this plant [2]. In continuing our search for industrially useful essential oils in Sri Lanka, we initiated an investigation of the heartwood of *E. monogynum*. The essential oil obtained by the initial hydrodistillation of the heartwood contained a mixture of monoterpenoids along with three high molecular weight compounds, one of which was identified as *ent*-beyer-15-ene (**1**) by Gas chromatography–mass spectrometry (GC–MS) and confirmed by ^1^H and ^13^C NMR data. With the view to identifying the remaining two high molecular weight compounds, steam distillation of the heartwood of *E. monogynum* has been carried out initially at 40 psi for 24 h (Stage 1) exhaustively to remove the monoterpene fraction and then at 70 psi for additional 12 h (Stage 2) to obtain mainly the high molecular weight fraction. The essential oil obtained in the Stage 2 showed the presence of three compounds on GC–MS analysis, one of which was identified as *ent*-beyer-15-ene (**1**). The remaining two compounds were identified as erythroxylol A (**2**) and an unsaturated diterpene aldehyde, *ent*-beyer-15-en-19-al (**3**) by the analysis of their ^1^H and ^13^C NMR data. This is the first report of **3** from this plant as well as the complete assignment of its ^1^H and ^13^C NMR data supported by 2D NMR spectroscopic data. Compound **3** has been reported previously as an unstable oil from the timber of *Erythroxylum zambesicum*. Its structure had been established by its conversion to the corresponding alcohol (erythroxylol A, **2**), and the observation of an aldehyde group, three tertiary methyl groups and a *cis* double bond in its ^1^H NMR spectrum [3]. Prior to the report by Ansell [3] it had been reported as a semisynthetic product obtained from the oxidation of erythroxylol A (**2**) and to be a low melting solid (m. p. 63–65 °C), stable at 0 °C under nitrogen. The structure had been established by reducing it to *ent*-beyer-15-ene via its ethylene thioacetal [4]. Compound **3** has also been reported from *Viguiera grammatoglossa* [5] and *Myriocephalus stuartii* [6].

**Fig. 1:**
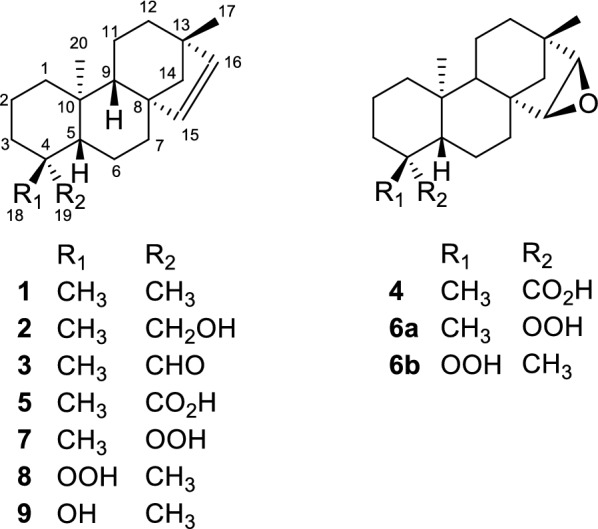
Structures of *ent*-beyer-15-ene (**1**), *ent*-beyer-15-en-19-ol (erythroxylol A) (**2**), *ent*-beyer-15-en-19-al (**3**) and auto-oxidation products of **3**

Compound **3** was found to be unstable at room temperature converting into more polar compounds. Of these the major compound was isolated and identified as 15,16-epoxy-*ent*-beyeran-19-oic acid (**4**). According to published data, this is the first report of the auto-oxidation of an aldehyde group to a carboxylic acid group with the concurrent epoxidation of a double bond in the same compound. Prompted by this observation we set-up an experiment to study this auto-oxidation process. In this experiment when **3** was exposed to air as a solution in cyclohexane at room temperature (28–30 °C) it underwent auto-oxidation to give two major products and five minor products. Of the two major products, the more polar one was found to be **4** while the other major compound was identified as *ent*-beyer-15-en-19-oic acid (**5**). Further investigation of the reaction mixture enabled us to isolate and identify the five minor compounds produced during this auto-oxidation process as, 15,16-epoxy-19-*nor*-*ent*-beyeran-4*α*-hydroperoxide (**6a**), 15,16-epoxy-18-*nor*-*ent*-beyeran-4*β*-hydroperoxide (**6b**), *ent*-beyer-19-*nor*-15-en-4*α*-hydroperoxide (**7**), *ent*-beyer-18-*nor*-15-en-4*β*-hydroperoxide (**8**), and *ent*-beyer-18-*nor*-15-en-4*β*-ol (**9**). Herein we report the occurrence of **3** in *E. monogynum* along with its complete assignment of ^1^H and ^13^C NMR data and the structure elucidation of compounds **4, 5, 6a, 6b, 7–9** (Fig. 1) utilizing their spectroscopic data. The plausible pathway of the formation of these compounds during the auto-oxidation of **3** is also discussed.

## Results and discussion

Heartwood of *E. monogynum* was subjected to steam distillation in two stages. The oil obtained in stage 1 was found to contain a mixture of monoterpenoids along with three high molecular weight compounds by GC–MS analysis (see Additional file 2). The oil obtained in stage 2 was shown to consist of mainly the above three major compounds by GC–MS analysis. The oil obtained in stage 2 was subjected to silica gel column chromatography to isolate these three compounds.

**Fig. 2:**
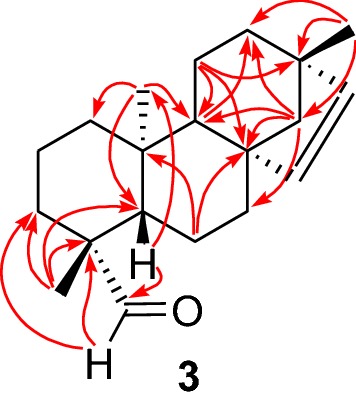
Selected HMBCs of **3**

Compound **3**, a viscous liquid, was identified as *ent*-beyer-15-en-19-al from the following spectroscopic data. It showed the molecular ion at *m*/*z* 286.2 [M]^+^. Its ^1^H and ^13^C NMR spectra (Tables 1 and 2) together with distortionless enhancement by polarization transfer (DEPT)135 and heteronuclear single quantum coherence (HSQC) data showed the presence of three methyls attached to quaternary carbons (*δ*_H_ 0.99 s, 1.00 s, 0.60 s and *δ*_C_ 24.4, 24.8, 14.6), eight methylenes, five methines, two of which are olefinic [*δ*_H_ 5.68 d (*J *= 5.6 Hz), 5.46 d (*J *= 5.6 Hz); *δ*_C_ 134.5, 136.8] and one which is aldehydic [*δ*_H_ 9.75 d (*J *= 1.4 Hz); *δ*_C_ 205.9] and four quaternary carbons accounting for C_20_H_30_O. Because the aldehyde carbonyl and the olefinic double bond accounted for two degrees of unsaturation, it was evident that **3** possesses a tetracyclic framework. NMR data together with connectivity of quaternary methyl groups, non-protonated carbons, methylenes and methines which were established by the analysis of heteronuclear multiple bond correlations (HMBC) (Fig. 2) showed that it had an *ent*-beyerene skeleton. Beyerane diterpenoids are common to *Erythroxylum* species [3, 4]. The tertiary methyl group at *δ*_H_ 1.00 (s) assigned to CH_3_-18 showed HMBC correlations with C-3 (*δ*_C_ 34.3), C-4 (*δ*_C_ 48.3), C-5 (*δ*_C_ 56.8) and the aldehyde carbonyl carbon (*δ*_C_ 205.9), placing the methyl and aldehyde groups at C-4. The tertiary methyl group at *δ*_H_ 0.99 showed HMBC correlations with the olefinic carbon at *δ*_C_ 136.8 (C-16), methylene carbons at *δ*_C_ 32.9 (C-12) and 61.0 (C-14) and quaternary carbon at *δ*_C_ 43.7 (C-13), placing it at C-13. The remaining tertiary methyl group at *δ*_H_ 0.60 showed HMBC correlations with the methine carbons at *δ*_C_ 56.8 (C-5), and 51.7 (C-9) and with methylene carbon at *δ*_C_ 38.7 (C-1) placing it at C-10. The olefinic proton at *δ*_H_ 5.46 assigned to H-16 showed HMBC correlations with the quaternary carbon at *δ*_C_ 43.7 (C-13) while the remaining olefinic proton at *δ*_H_ 5.68 showed HMBC correlation with the quaternary carbon at *δ*_C_ 48.9 (C-8) placing it at C-15, while both the olefinic protons showed HMBC correlations with the methylene carbon at *δ*_C_ 61.0 (C-14). Compound **3** underwent reduction with NaBH_4_ to give *ent*-beyer-15-en-19-ol (erythroxylol A) (**2**) confirming its structure as *ent*-beyer-15-en-19-al.

**Table 1:** ^1^H NMR (400 MHz) Spectroscopic Data (*δ*) of Compounds **3**, **4**, **6a**, **6b**, **7,** and **8** in CDCl_3_

#	**3**	**4**	**6a**	**6b**	**7**	**8**
1	0.88 m, 1.64 m	0.89 m, 1.69 m	0.87 m, 1.67 m	0.89 m, 1.69 m	0.88 m, 1.66 m	0.89 m, 1.57 m
2	1.41 m, 1.52 m	1.44 m	1.39 m, 1.66 m	1.40 m, 1.72 m	1.37 m, 1.64 m	1.56 m, 1.44 m
3	0.98 m, 2.11 m	1.01 m, 2.15 m	1.15 m, 2.16 m	1.37 m, 1.64 m	1.14 m, 2.16 m	1.67 m, 1.71 m
4	–	–	–	–	–	–
5	1.20 m	1.12 m	1.12 m	1.54 m	1.10 m	1.54 m
6	1.70 m, 1.86 m	1.91 m	1.51 m^a^	1.61 m^b^	1.24 m, 1.51 m	1.36 m, 1.64 m
7	1.34 m, 1.71 m	1.17 m, 1.90 m	1.18 m, 1.90 m	1.27 m, 1.88 m	1.28 m, 1.64 m	1.37 m, 1.62 m
8	–	–	–	–	–	–
9	0.99 m	1.13 m	1.24 m	1.26 m	0.95 m	1.08 m
10	–	–	–	–	–	–
11	1.53 m	1.51 m	1.50 m^a^	1.50 m^b^	1.72 m	1.26 m, 1.51 m
12	1.25 m	1.37 m, 1.64 m	1.37 m, 1.64 m	1.37 m, 1.64 m	1.25 m	1.26 m
13	–	–	–	–	–	–
14	1.02 m, 1.46 m	0.53 d (*J* = 11.0 Hz) (*β*-H) 1.16 d (*J* = 11.0 Hz) (*α*-H)	0.50 d (*J *= 10.8 Hz) (*β*-H) 1.16 d (*J *= 10.8 Hz) (*α*-H)	0.55 d (*J *= 10.9 Hz) (*β*-H) 1.17 d (*J *= 10.9 Hz) (*α*-H)	1.00 m, 1.44 m	1.04 m, 1.45 m
15	5.68 d (*J* = 5.6 Hz)	3.43 d (*J* = 3.0 Hz)	3.47 d (*J *= 3.0 Hz)	3.40 d (*J *= 3.0 Hz)	5.71 d (*J *= 5.7 Hz)	5.67 d (*J *= 5.7 Hz)
16	5.46 d (*J* = 5.6 Hz)	3.04 d (*J* = 3.0 Hz)	3.02 d (*J *= 3.0 Hz)	3.03 d (*J *= 3.0 Hz)	5.45 d (*J *= 5.7 Hz)	5.46 d (*J *= 5.7 Hz)
17	0.99 s	1.02 s	1.01 s	1.02 s	0.99 s	0.99 s
18	1.00 s	1.25 s	1.30 s	–	1.28 s	–
19	9.75 d (J = 1.4 Hz)	–	–	1.13 s	–	1.11 s
20	0.60 s	0.84 s	1.03 s	0.92 s	0.85 s	0.74 s

^a^Values may be interchanged

^b^Values may be interchanged

**Table 2:** ^13^C NMR (100 MHz) Spectroscopic Data (*δ*) of Compounds **3**, **4**, **6a**, **6b**, **7**, and **8** in CDCl_3_

#	**3**		**4**		**6a**		**6b**		**7**		**8**	
1	38.7	CH_2_	39.7	CH_2_	39.3	CH_2_	38.3	CH_2_	39.1	CH_2_	38.13	CH_2_
2	18.5	CH_2_	19.1	CH_2_	17.7	CH_2_	18.7	CH_2_	17.8	CH_2_	19.3	CH_2_
3	34.3	CH_2_	37.7	CH_2_	34.9	CH_2_	35.3	CH_2_	34.9	CH_2_	35.7	CH_2_
4	48.3	C	43.7	C	84.0	C	84.7	C	84.2	C	85.0	C
5	56.8	CH	56.9	CH	55.9	CH	50.5	CH	55.9	CH	50.4	CH
6	19.6	CH_2_	21.3	CH_2_	19.35^a^	CH_2_	19.3^b^	CH_2_	20.4	CH_2_	19.1	CH_2_
7	37.4	CH_2_	33.5	CH_2_	33.3	CH_2_	32.3	CH_2_	37.4	CH_2_	36.4	CH_2_
8	48.9	C	44.3	C	44.1	C	44.2	C	48.9	C	48.9	C
9	51.7	CH	55.7	CH	56.1	CH	56.3	CH	52.4	CH	52.7	CH
10	37.6	C	38.2	C	37.5	C	38.4	C	37.2	C	38.10	C
11	20.6	CH_2_	19.5	CH_2_	19.44^a^	CH_2_	19.5^b^	CH_2_	19.6	CH_2_	20.4	CH_2_
12	32.9	CH_2_	35.4	CH_2_	35.4	CH_2_	35.5	CH_2_	33.0	CH_2_	33.1	CH_2_
13	43.7	C	38.9	C	39.1	C	39.0	C	43.7	C	43.7	C
14	61.0	CH_2_	46.6	CH_2_	46.9	CH_2_	46.7	CH_2_	61.2	CH_2_	61.1	CH_2_
15	134.5	CH	55.9	CH	56.0	CH	55.9	CH	135.2	CH	135.0	CH
16	136.8	CH	60.1	CH	60.20	CH	60.16	CH	136.4	CH	136.6	CH
17	24.4	CH_3_	21.4	CH_3_	21.5	CH_3_	21.5	CH_3_	24.9	CH_3_	24.9	CH_3_
18	24.8	CH_3_	29.0	CH_3_	24.7	CH_3_	–		24.8	CH_3_	–	
19	205.9	CH	183.9	C	–		18.3	CH_3_	–		18.4	CH_3_
20	14.6	CH_3_	14.2	CH_3_	16.0	CH_3_	15.6	CH_3_	15.5	CH_3_	14.9	CH_3_

^a^Values may be interchanged

^b^Values may be interchanged

The remaining two compounds isolated from the essential oil were identified as *ent*-beyerene (**1**) by its GC–MS data [7] and comparison with reported ^13^C NMR data [8] (Additional file 2: Table S1) and *ent*-beyer-15-en-19-ol (erythroxylol A) (**2**) by the analysis of its ^1^H and ^13^C NMR data and comparison with reported ^13^C NMR data [8] (Additional file 2: Table S1). These two compounds have been reported previously from the timber of *E. monogynum* [4].

Thin layer chromatographic (TLC) analysis of a solution of compound **3** in cyclohexane showed that the compound was unstable when exposed to air and decomposed to a number of compounds, all of which were more polar than the parent compound. It was observed that the decomposition of **3** was prevented by the addition of butylated hydroxy toluene (BHT) to the solution supporting the view that the decomposition was an auto-oxidation occurring through a free radical mechanism. Compound **3** was found to be stable when stored at 0 °C under nitrogen in the absence of a solvent.

**Fig. 3:**
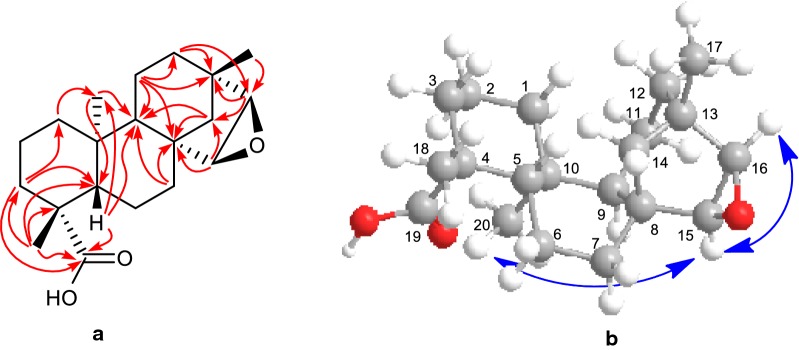
**a** Selected HMBCs and **b** NOEs observed in Selective NOESY gradient experiments of **4**

Periodic TLC analysis of a solution of **3** in cyclohexane indicated a changing pattern of spots characteristic of a radical reaction, which stabilized after 14 days to give a pattern of six spots, two of which were present in larger amounts than the others. Compound **4**, the more polar one of the two major products was obtained as a viscous liquid, analyzed for C_20_H_30_O_3_ by a combination of high-resolution electro-spray ionization mass spectrometry (HRESIMS) and ^13^C NMR spectroscopy. The ^1^H and ^13^C NMR spectra (Tables 1 and 2) together with HSQC data of **4** indicated that its structure was very similar to that of **3**. In comparing the ^13^C NMR spectrum of **4** with that of **3**, the oxidation of the aldehyde group to a carboxylic acid group is clearly indicated by the appearance of a signal at *δ*_C_ 183.9 and the loss of the signal at *δ*_C_ 205.9. This structural change is also reflected in the ^1^H NMR spectrum, where the signal due to aldehyde proton at *δ*_H_ 9.75 in **3** is absent in **4**. Epoxidation of the double bond is indicated by the loss of two olefinic CH groups [*δ*_H_ 5.68 d, (*J *= 5.6 Hz), 5.46 d, (*J *= 5.6, Hz); *δ*_C_ 134.5, 136.8] and the appearance of two new oxygenated methines [*δ*_H_ 3.43 d, (*J *= 3.0 Hz), 3.04 d, (*J *= 3.0 Hz); *δ*_C_ 55.9, 60.1). The tertiary methyl group at *δ*_H_ 1.25 assigned to 18-H_3_ [9] showed HMBC correlations with C-3 (*δ*_C_ 37.7), C-4 (*δ*_C_ 43.7), C-5 (*δ*_C_ 56.9) and the carboxyl carbonyl carbon (*δ*_C_ 183.9), placing the methyl and carboxylic acid groups at C-4. The tertiary methyl group at *δ*_H_ 1.02 showed HMBC correlations with the oxygenated methine carbon at *δ*_C_ 60.1 (C-16) and methylene carbon at *δ*_C_ 46.6 (C-14) placing it at C-13. The remaining tertiary methyl group at *δ*_H_ 0.84 showed HMBC correlations with the methine carbons at *δ*_C_ 56.9 (C-5), and 55.7 (C-9) placing it at C-10. The oxygenated methine proton at *δ*_H_ 3.04 (d, *J *= 3.0 Hz) assigned to H-16 showed HMBC correlation with the quaternary carbon at *δ*_C_ 38.9 (C-13) and the remaining oxygenated methine proton at *δ*_H_ 3.43 (d, *J *= 3.0 Hz) showed HMBC correlation with the quaternary carbon at *δ*_C_ 44.3 (C-8) placing it at C-15, while both the oxygenated methine protons showed HMBC correlations with the methylene carbon at *δ*_C_ 46.6 (C-14). Connectivity of remaining carbons was established by the HMBC correlations as shown in Fig. 3a. Irradiation of 15-H [*δ*_H_ 3.43 d (*J *= 3.0 Hz)] in the Selective nuclear Overhauser effect spectroscopy (NOESY) Gradient experiment exhibited enhancement of 20-H_3_ (*δ*_H_ 0.84 s) (Fig. 3b) (Additional file 2: Figure S15) suggesting the *α*- orientation of the 15-H, confirming the formation of epoxide from the *β*-face (*exo* epoxide). The formation of the epoxide brings about clear differentiation of the two H atoms on 14-C with one H moving up-field to *δ*_H_ 0.53 as a doublet with a coupling constant of 11.0 Hz, typical for a geminal coupling. Thus, structure of **4** was determined to be 15,16-epoxy-*ent*-beyeran-19-oic acid.

Compound **5**, the less polar major product was obtained as a colorless viscous liquid. The ^1^H and ^13^C NMR spectroscopic data together with HSQC and DEPT135 of **5** (Additional file 2: Table S2 and Figures S16–S21) showed very close resemblance of its structure to those of **3** and **4**. The presence of a carboxylic acid C = O was indicated by the signal at *δ*_C_ 183.8 in its ^13^C NMR spectrum while the presence of the two olefinic protons as in the case of **3** were clearly evident by the presence of the signals at *δ*_H_ 5.73 d (*J *= 5.7 Hz); *δ*_C_ 134.8 (CH-15) and *δ*_H_ 5.45 d (*J *= 5.7 Hz); *δ*_C_ 136.5 (CH-16). Thus **5** was identified as *ent*-beyer-15-en-19-oic acid by the comparison of its ^13^C NMR data with reported data for *ent*-beyer-15-en-19-oic acid (Additional file 2: Table S2), which has been isolated from *Elaeoselinum asclepium* [10]. As the reported ^13^C NMR data of this compound was not supported by 2D NMR (HSQC and HMBC) spectroscopic data and its ^1^H NMR data was not available, we assigned the ^1^H and ^13^C NMR data of **5** with the help of HSQC and HMBC correlations (Additional file 2: Table S2).

**Fig. 4:**
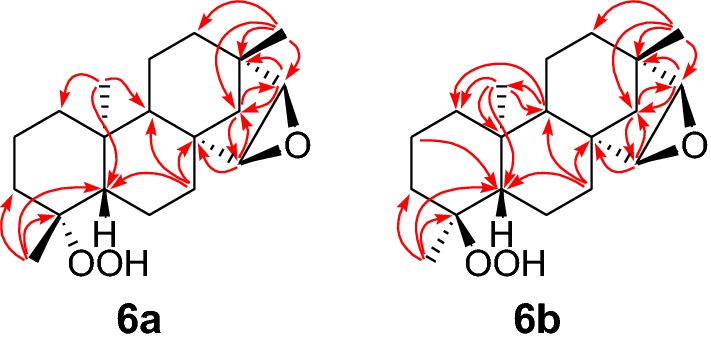
Selected HMBCs of **6a** and **6b**

The most polar minor auto-oxidation product (**6**) obtained as a colorless viscous liquid was determined to be a mixture of two isomers **6a** and **6b** epimeric at C-4 in 1:2 ratio from the following evidence. GC–MS analysis showed two peaks in the GC, both of which showed the same [M]^+^*m*/*z* 306.4; ^1^H NMR spectrum of **6** showed two doublets at *δ*_H_ 3.40 (*J *= 3.0 Hz) and *δ*_H_ 3.47 (*J *= 3.0 Hz) in 2:1 ratio and 6 tertiary methyl groups and ^13^C NMR spectrum showed 37 carbon signals. ^1^H and ^13^C NMR spectroscopic data (Tables 1 and 2) together with HSQC and DEPT135 revealed that it contained eight quaternary carbons of which two are oxygenated (*δ*_C_ 84.7 and 84.0), eight methines of which four are oxygenated [*δ*_H_ 3.40 d (*J *= 3.0 Hz), 3.03 d (*J *= 3.0 Hz), 3.47 d (*J *= 3.0 Hz), 3.02 d (*J *= 3.0 Hz); *δ*_C_ 55.9, 60.16, 56.0, 60.20], sixteen methylenes and six tertiary methyls (*δ*_H_ 0.92 s, 1.02 s, 1.13 s, 1.03 s, 1.01 s, 1.30 s; *δ*_C_ 15.6, 21.5, 18.3, 16.0, 21.5, 24.7) of which two overlapped at *δ*_C_ 21.5 in the ^13^C NMR spectrum. These data suggested that these two isomers lack a carbon atom from each of these two isomers and presence of 15(16) epoxide as in the case of compound **4**. ^13^C NMR spectrum of **6** did not show either CHO or CO_2_H carbon signals but showed two signals at *δ*_C_ 84.7 and 84.0 for oxygenated quaternary carbons indicating that the 18 (or 19) C has been lost from the beyerane skeleton and a hydroperoxide (-OOH) group has been attached to 4-C, suggesting the possibility of these two being C-4 epimers of each other. Analysis of HMBC correlations (Fig. 4) permitted the unambiguous assignment of NMR signals of each of the epimers (Tables 1 and 2).

**Fig. 5:**
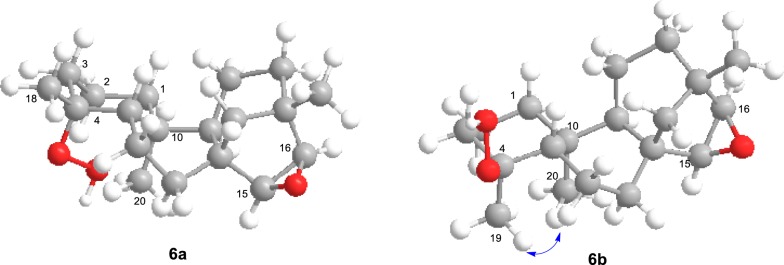
3D structures of **6a** and **6b** showing NOE observed in Selective NOESY gradient experiments of **6b**

Differentiation of epimers **6a** and **6b** and the relative configuration at C-4 in **6b** was achieved by a series of Selective NOESY Gradient experiments (Fig. 5). Irradiation of the signal at *δ*_H_ 0.92 (20-H_3_) in a Selective NOESY Gradient experiment caused enhancement of the methyl signal at *δ*_H_ 1.13 (19-H_3_) and the oxygenated methine proton at *δ*_H_ 3.40 (15-H) suggesting that these two methyl groups and the oxygenated methine proton belong to the epimer **6b** and both methyl groups are on the same side of the molecule confirming α-orientation of the C-4 methyl group. Further it is evident that the H-15 is also *α*-orientated confirming the formation of epoxide from the *β*-face (*exo* epoxide) as in the case of **4**. Irradiation of the methyl signal at *δ*_H_ 1.13 (19-H_3_) caused enhancement of signal at *δ*_H_ 0.92 (20-H_3_) confirming the above (Additional file 2: Figures S29 and S30). When the signal at *δ*_H_ 1.30 [18-H_3_ (Me at C-4)] of **6a** was irradiated in a Selective NOESY Gradient experiment no enhancement of signals was observed confirming the *β*-orientation of this methyl group (Additional file 2: Figure S31).

Methyl protons at *δ*_H_ 1.13 (19-H_3_) in compound **6b** showed HMBC correlations with the quaternary carbon at *δ*_C_ 84.7 (C-4), methylene carbon at *δ*_C_ 35.3 (C-3) and methine carbon at *δ*_C_ 50.5 (C-5) placing this methyl at C-4. Further the methylene proton signals at *δ*_H_ 1.72 and 1.40 (2-H_2_) and the methine proton signal at *δ*_H_ 1.54 (H-5) showed HMBC correlations with the quaternary carbon at *δ*_C_ 84.7 (C-4). The methyl protons at *δ*_H_ 0.92 assigned to CH_3_-20 showed HMBC correlations to methylene carbon at *δ*_C_ 38.3 (C-1), quaternary carbon at *δ*_C_ 38.4 (C-10), and methine carbons at 50.5 (C-5) and 56.3 (C-9). The protons of remaining methyl group at *δ*_H_ 1.02 (17-H_3_) showed HMBC correlations to quaternary carbon at *δ*_C_ 39.0 (C-13), methine carbon at *δ*_C_ 60.16 (C-16), methylene carbons at *δ*_C_ 35.5 (C-12) and 46.7 (C-14). Methine protons of the epoxide ring at *δ*_H_ 3.03 (H-16) and *δ*_H_ 3.40 (H-15) showed HMBC correlations to quaternary carbons *δ*_C_ 39.0 (C-13) and *δ*_C_ 44.2 (C-8) respectively while both of them showed correlations to *δ*_C_ 46.7 (C-14). The remaining key HMBC correlations useful for the establishment of connectivity in the molecule are shown in Fig. 4.

It appears that the remaining three methyl signals in the ^1^H NMR spectrum of **4** responsible for tertiary methyl groups belong to the epimer **6a**. The methyl protons at *δ*_H_ 1.30 (18-H_3_) in compound **6a** showed HMBC correlations with the quaternary carbon at *δ*_C_ 84.0 (C-4), methylene carbon at *δ*_C_ 34.9 (C-3) and methine carbon at *δ*_C_ 55.9 (C-5) placing this methyl at C-4. Methyl protons at *δ*_H_ 1.03 (20-H_3_) showed HMBC correlations with the methylene carbon at *δ*_C_ 39.3 (C-1) and methine carbons at *δ*_C_ 55.9 (C-5) and *δ*_C_ 56.1 (C-9). The remaining methyl group at *δ*_H_ 1.01 (17-H_3_) showed HMBC correlations to quaternary carbon at *δ*_C_ 39.1 (C-13), methine carbon at *δ*_C_ 60.20 (C-16), methylene carbons at *δ*_C_ 35.4 (C-12) and 46.9 (C-14). Methine protons of the epoxide ring at *δ*_H_ 3.47 (15-H) and *δ*_H_ 3.02 (16-H) showed HMBC correlations to quaternary carbons *δ*_C_ 44.1 (C-8) and 39.1 (C-13) respectively while both of them showed correlations to *δ*_C_ 46.9 (C-14). The remaining key HMBC correlations useful for the establishment of connectivity in the molecule are shown in Fig. 4. Although these two epimers were inseparable under normal phase chromatographic techniques, it was possible to separate them by reverse phase analytical TLC and the two compounds were subjected to HRESIMS to determine their molecular formulae. Compound **6a** analyzed for C_19_H_30_O_3_, *m*/*z* 307.22594 [M + H]^+^ (calcd. for C_19_H_31_O_3_, 307.22746), *m*/*z* 305.21215 [M – H]^–^ (calcd. for C_19_H_29_O_3_, 305.21180) and **6b** analyzed for C_19_H_30_O_3_, *m*/*z* 307.22617 [M + H]^+^ (calcd. for C_19_H_31_O_3_, 307.22746), *m*/*z* 305.21217 [M—H]^–^ (calcd. for C_19_H_29_O_3_, 305.21180). These data confirmed the structures of **6a** and **6b** as15,16-epoxy-19-*nor*-*ent*-beyeran-4*α*-hydroperoxide and 15,16-epoxy-18-*nor*-*ent*-beyeran-4*β*-hydroperoxide respectively. The ^1^H and ^13^C NMR spectra obtained for these two samples were found to be consistent with the assignments made for **6a** and **6b** based on the above analysis of the spectra of their mixture **6** (Additional file 2: Figures S32–S37).

**Fig. 6:**
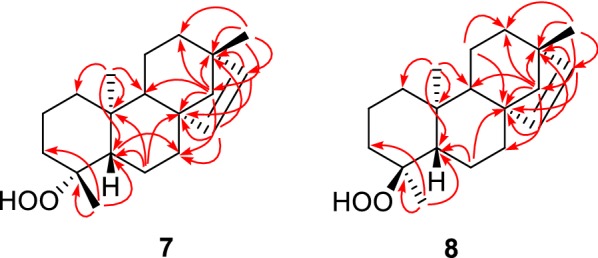
Selected HMBC correlations of **7** and **8**

Compound **7** obtained as a colorless viscous liquid, analyzed for C_19_H_30_O_2_ by a combination of HRESIMS and ^13^C NMR spectroscopy. ^1^H and ^13^C NMR spectroscopic data (Tables 1 and 2) together with HSQC and DEPT135 of compound **7** showed the presence four quaternary carbons of which one is oxygenated (*δ*_C_ 84.2), four methines of which two are olefinic [*δ*_H_ 5.71 d (*J *= 5.7 Hz), 5.45 d (*J *= 5.7 Hz); *δ*_C_135.2, 136.4], eight methylenes and three tertiary methyls (*δ*_H_ 0.99 s, 1.28 s, and 0.85 s; *δ*_C_ 24.9, 24.8, and 15.5). Comparison of this data with the corresponding data for **6a**/**6b** suggested that **7** could be a C-4 hydroperoxide similar to **6a** or **6b** but with a C-15 olefinic double bond. Compound **8** obtained as a colorless viscous liquid, analyzed for C_19_H_30_O_2_ by a combination of HRESIMS and ^13^C NMR spectroscopy. ^1^H and ^13^C NMR spectroscopic data (Tables 1 and 2) together with HSQC and DEPT135 of compound **8** also showed the presence four quaternary carbons of which one is oxygenated (*δ*_C_ 85.0), four methines of which two are olefinic [*δ*_H_ 5.67 d (*J *= 5.7 Hz), 5.46 d (*J *= 5.7 Hz); *δ*_C_ 135.0, 136.6], eight methylenes and three tertiary methyls (*δ*_H_ 0.99 s, 1.12 s, and 0.74 s; *δ*_C_ 24.9, 18.4, and 14.9) suggesting that this could be the C-4 epimer of **7**. Unambiguous assignment of ^1^H and ^13^C NMR signals of each of the compounds **7** and **8** was enabled by the analysis of HMBC correlations of respective compounds (Fig. 6).

Since **7** and **8** are C-4 epimers of each other it was necessary to establish the stereochemistry at C-4 in these two compounds. Comparison of the ^1^H and ^13^C NMR chemical shifts of C-4 methyl groups (18-H_3_ or 19-H_3_) and 20-H_3_ of these two compounds with those of **6a** and **6b** (Tables 1 and 2) indicated that the hydroperoxide group in **7** is *α*-oriented and in **8** it is *β*-oriented. This was further confirmed by series of Selective NOESY Gradient experiments. Irradiation of the signal at *δ*_H_ 0.74 (20-H_3_) of **8** in a Selective NOESY Gradient experiment caused enhancement of the methyl signal at *δ*_H_ 1.12 (19-H_3_) suggesting that these two methyls are in the same side of the molecule indicating the *α*-orientation of the C-4 methyl group. Irradiation of the methyl signal at *δ*_H_ 1.12 (19-H_3_) caused enhancement of signal at *δ*_H_ 0.74 (20-H_3_) further confirming the above suggestion (Additional file 2: Figures S50 and S51). Irradiation of the signal at *δ*_H_ 0.85 (20-H_3_) or *δ*_H_ 1.28 (18-H_3_) of **7** did not cause enhancements of either signals (Additional file 2: Figures S43 and S44) suggesting that these two methyl groups are not in the same face and hence suggested the *β*-orientation of methyl group at C-4 (18-H_3_). Thus, the compounds **7** and **8** were identified as *ent*-beyer-19-*nor*-15-en-4*α*-hydroperoxide and *ent*-beyer-18-*nor*-15-en-4*β*-hydroperoxide respectively.

Compound **9** was obtained as a white, amorphous solid. Comparison of ^1^H and ^13^C NMR spectroscopic data (Additional file 2: Table S3) together with HSQC and DEPT135 of compound **9** with those of **7**/**8** suggested that this is a C-4 alcohol with C-15 olefinic double bond. Although both *ent*-beyer-18(19)-*nor*-15-en-α- and β-ols were known, their spectroscopic assignments were not supported by 2D NMR (HSQC and HMBC) spectroscopic data. Hence, we assigned the ^1^H and ^13^C NMR data of this compound with the help of HSQC and HMBC data. The HMBC correlations useful for the assignment of ^1^H and ^13^C NMR signals confirming the structure of compound **9** are shown in (Additional file 2: Table S3). Stereochemistry at C-4 has been established by carrying out Selective NOESY Gradient experiments. Irradiation of the signal at *δ*_H_ 0.71 (20-H_3_) of **9** in a Selective NOESY Gradient experiment caused enhancement of the methyl signal at *δ*_H_ 1.14 (19-H_3_) indicating that these two methyl groups are in the same side of the molecule confirming *α*- orientation of the C-4 methyl group. Irradiation of the methyl signal at *δ*_H_ 1.14 (19-H_3_) caused enhancement of signal at *δ*_H_ 0.71 (20-H_3_) further confirming the above *α*- orientation of the C-4 methyl group (Additional file 2: Figures S57 and S58). Thus, the compound **9** was identified as *ent*-beyer-18-*nor*-15-en-4*β*-ol, which has been isolated previously as a natural product from *E. monogynum* [11].

**Scheme 1:**
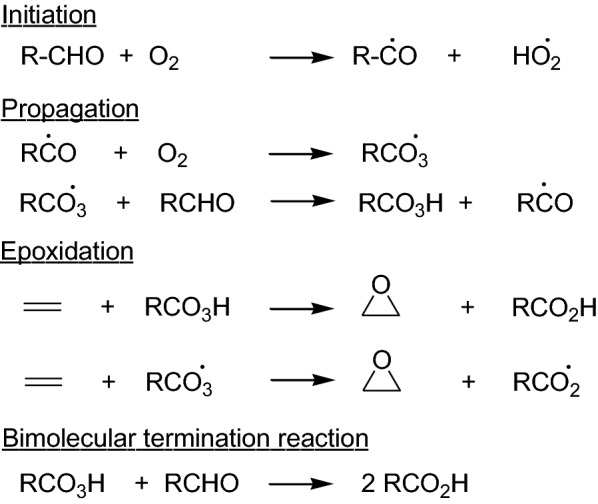
Auto-oxidation of an aldehyde group with concurrent epoxidation of a carbon–carbon double bond

The susceptibility of 4-axial aldehyde groups in the diterpenes towards auto-oxidation giving rise to carboxylic acids and hydroperoxides via radical mechanisms has been previously reported [10, 12, 13]. Although **3** was known to be an unstable compound, there have been no previous reports on the products obtained from the auto-oxidation of **3.** The formation of the epoxy compounds **4, 6a** and **6b** during the auto-oxidation of **3** can be rationalized by considering the steps involved in the auto-oxidation of aldehydes to carboxylic acids. Auto-oxidation of an aldehyde to the corresponding carboxylic acid is a facile reaction and takes place via a free radical mechanism [14, 15]. Acyl peroxy radicals and per-acids are generated as intermediates during the reaction (Scheme 1). Both these species are capable of epoxidizing an olefinic double bond. Thus, both catalyzed and uncatalyzed processes for the epoxidation of an olefin coupled to the oxidation of an aldehyde to the corresponding carboxylic acid by molecular oxygen have been reported [16–20].

We envisage the epoxidation taking place via an intermolecular reaction between a C-19 peroxy group and the C-15,16 olefin group giving rise to the *exo*-epoxide because the approach of the two species to form an *endo*-epoxide would be sterically hindered. Steric hindrance of the α-face of the molecule exerted by the axial 20-methyl group would also allow epoxidation to compete with the usual bimolecular termination reaction (Scheme 1) between the peroxy acid and the aldehyde to give two molecules of carboxylic acids. This interpretation is supported by the observation that the C-20,29 double bond of betulonaldehyde does not undergo epoxidation during the auto-oxidation of its unhindered C-28 formyl group [21] which can be approached without hindrance from the β-face of the molecule. It is interesting to note that betulonaldehyde on auto-oxidation gave in addition to betulonic acid, two epimeric C-17 hydroperoxides which would correspond to **7** and **8** in the current study. In a related process, 14-hydroxypimara-8,15-dien-19-oic acid has been isolated from the auto-oxidation of pimara-8(14),15-dien-19-al (which also has an axial aldehyde group) [12]. It has been proposed that the 14-hydroxy compound arises from the ring opening hydrolysis followed by dehydration of the corresponding 8(14)-epoxy carboxylic acid on the basis of chromatographic evidence, although such an epoxy carboxylic acid has not been isolated from the reaction mixture from the auto-oxidation reaction.

**Scheme 2:**
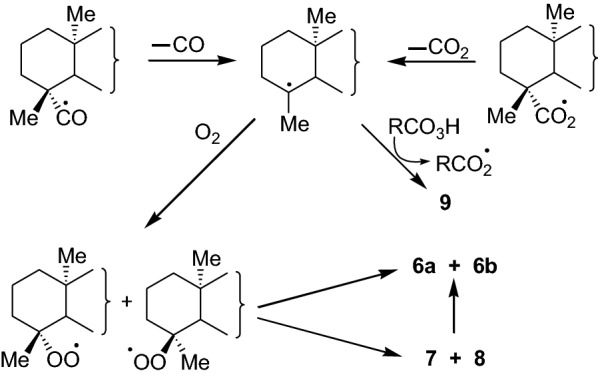
Possible pathways of formation of the epimeric epoxy hydroperoxides **6a** and **6b,** epimeric hydroperoxides **7** and **8,** and the alcohol **9**

The formation of the epimeric epoxy hydroperoxides **6a** and **6b,** the epimeric hydroperoxides **7** and **8**, and the alcohol **9** can be explained (Scheme 2) as arising from the reactions of the tertiary cycloalkyl radical at C-4 which can be formed by the decarbonylation of the acyl radical and decarboxylation of the acyloxy radical that are formed during the auto-oxidation process [12, 15].

The ease of decarbonylation of tertiary acyl radicals is well known. The decarbonylation and decarboxylation reactions are further aided by the loss of steric strain when the *sp*^3^ carbon (C-4) changes to a planar *sp*^2^ carbon removing the 1,3-diaxial interaction of the radical on C-19 with the 20 *α*- methyl group. The 20 *α*- methyl group also directs the approach of molecular oxygen and the peracid group to preferentially approach the planar C-4 radical from the *β*- face by sterically hindering the *α*- face approach. This results in the excess of **6b** over **6a,** as observed in the ^1^H NMR spectrum of **6,** (the mixture of **6a** and **6b**) isolated by column chromatography of the auto-oxidation reaction mixture. Further, of the two possible epimeric alcohols, only the *β*- alcohol **9** could be detected. However, we note that the preference for the *β*- face approach of molecular oxygen is not reflected in the relative isolated yields of the hydroperoxides **7** and **8**, probably due to experimental losses of yield. Although **9** has been reported as a natural product isolated from the timber of *E. monogynum* [11], our results support the suggestion by Caputo [13] and Tanaka [12] that it was an artifact arising from the auto-oxidation of **3.**

The auto-oxidation of **3** is inhibited when it is found as a component of the essential oil of *E. monogynum* by the presence of other components such as **4,** which can act as radical chain breakers. However, we note that no trace of **3** could be detected by GC analysis in a 1 year old sample of the essential oil which had been stored under ambient conditions.

## Conclusions

The auto-oxidation of the aldehyde group of *ent*-beyer-15-en-19-al to a carboxylic acid group is a facile process and takes place both with and without the concurrent epoxidation of the 15,16-double bond. Our results suggest that 4-hydroxy-19-nor-*ent*-beyer-15-ene which has been reported previously as a natural product from *E. monogynum* may be an artefact arising from the auto-oxidation reaction. Steric hindrance exerted by the axial 20-methyl group plays a determining role in the product distribution of the auto-oxidation reaction. The usage of the essential oil from the heartwood of *E. monogynum* in perfumery will be limited by the instability in the presence of oxygen of *ent*-beyer-15-en-19-al, which is a major component of the oil.

## Experimental

### General experimental procedures

Optical rotations were measured in CHCl_3_ with a BioBase Automatic polarimeter BK-P2. IR spectra were recorded as Attenuated total reflections spectra on a Perkin Elmer Spectrum 2. 1D and 2D NMR spectra were recorded in CDCl_3_ with a Bruker Ascend 400 spectrometer at 400 Mz for ^1^H NMR and 100 MHz for ^13^C NMR using residual CHCl_3_ as the internal reference. High resolution MS were measured on a Q-Exactive (ThermoScientific) equipment, with H-ESI source. Thin layer chromatography was carried out on Merck analytical normal phase (G60, F_254_, 0.2 mm) and reverse phase (Silica gel 60 RP-18, 0.25 mm) plates. Preparative layer chromatography was carried out on Analtech normal phase plates (G60, 0.5 mm). All solvents used for chromatography were of AR grade from Sigma-Aldrich. Compounds were visualized by spraying with anisaldehyde-sulfuric acid reagent made by mixing anisaldehyde (0.5 ml) with glacial acetic acid (10 ml), followed by 85 ml of methanol and concentrated sulfuric acid (5 ml). Dry column chromatography was carried out using Analtech silica gel (35–75 micron, 150 A). Column fractions were analyzed by TLC and similar fractions were combined and evaporated under reduced pressure. GC–MS analysis was carried out using an Agilent 7890 A GC system equipped with 5975C inert XL MSD Triple–Axis Detector, and HP-5 MS fused silica capillary with a (5% Phenyl)-methylpolysiloxane stationary phase (30 m × 0.25 mm Id, x 0.25 µm film thickness) capillary column. Helium (99.999%) was used as carrier gas. Mass spectra were acquired in the EI mode at 70 eV within the range of 40.5 to 500 mass units.

### Plant material

The heartwood of *E. monogynum* was collected from a tree found at a home garden in Weerawila, Sri Lanka (6˚14ʹ 56.9ʹʹ N, 81˚13ʹ 46.6ʹʹ E). The species was identified by Prof. D. S. A. Wijesundara, National Institute of Fundamental Studies, Hantane, Kandy, Sri Lanka (former Director of the Royal Botanic Gardens, Peradeniya and the National Herbarium of Sri Lanka). A voucher specimen (Voucher no.43-002-010) was deposited in the herbarium at the R & D division at LINK Natural Products, Sri Lanka.

### Extraction of essential oil

The dried heartwood (46 kg) of *E. monogynum* was cut into 8 mm pieces and subjected to steam distillation in two stages. A steam pressure of 40 psi was used, and the flow rate of the condensate was 500–600 mL min^−1^ during the first stage (24 h) to obtain 76.8 g of a pale yellow, light oil. Distillation was continued for an additional 12 h (stage 2) at a steam pressure of 70 psi and a condensate flow rate of 800–900 mL min^−1^ to obtain 40.8 g of a thick oil. Both fractions were dried over anhydrous sodium sulfate and stored at − 4 °C in a refrigerator. GC–MS analysis of the oil obtained in stage 2, indicated the presence of three major compounds, of which two were identified as *ent*-beyer-15-ene (**1**) and erythroxylol A (**2**). GC–MS analysis of the oil obtained in stage 1 showed that it also contained these three compounds, in lower concentrations along with lower boiling monoterpenoids.

### *Ent*-beyer-15-ene Diterpenoids from the essential oil of *E. monogynum*

The oil obtained in stage 2 of the above steam distillation (1.08 g) was subjected to dry column chromatography using gradient elution with hexane-dichloromethane and 70 fractions (10 mL) were collected. These were analyzed by TLC and fractions containing same compound were combined. Fraction 2 on evaporation under reduced pressure gave *ent*-beyer-15-ene (**1**, 230 mg, 21.00%) as a colorless, odiferous viscous liquid. Fractions 3–15 gave *ent*-beyer-15-en-19-al (**3,** 118 mg, 1.09%) as a colorless odiferous viscous liquid and fractions 38–60 gave erythroxylol A (**2**, 180 mg, 1.66%) as an off-white amorphous powder.

### *Ent*-beyer-15-ene (1)

A colorless viscous liquid; ${[\upalpha ]_{{\text{D}}}}^{{25}}$ +26.5° (*c* 0.061, CHCl_3_); IR (FT-ATR) ν_max_ 2920, 2844, 1386, 1364,750 cm^−1^; GC–MS (*t*_R_ 36.7 min) *m*/*z* 272.3, M^+^. Identity of *Ent*-beyer-15-ene was established by comparison with reported GC–MS and ^13^C NMR data [7, 8] (Additional file 2: Table S3).

### Erythroxylol A (2)

An off white amorphous solid; ${[\upalpha ]_{{\text{D}}}}^{{25}}$ +24.24° (*c* 0.0033, CHCl_3_); IR (FT-ATR) ν_max_ 3380, 2922, 2865, 2845, 1727, 1448, 1379, 1364, 1025. 973, 749; ^1^H and ^13^C NMR spectroscopic data (Additional file 2: Table S3); GC–MS (*t*_R_ 40.24 min) *m*/*z* 288.3, M^+^. Identity of erythroxylol A was established by comparison with reported ^13^C NMR data [8].

### Ent-beyer-15-en-19-al (3)

A colorless viscous liquid;${[\upalpha ]_{{\text{D}}}}^{{25}}$ +27.55° (*c* 0.075, CHCl_3_); IR (FT-ATR) ν_max_ 2931, 2866, 2846, 1716, 1450, 751 cm^−1^; ^1^H and ^13^C NMR spectroscopic data, see Tables 1 and 2; GC–MS (*t*_R_ 39.3 min) *m*/*z* 286.3, M^+^.

### Auto-oxidation of *ent*-beyer-15-en-19-al (3)

*Ent*-beyer-15-en-19-al) **(3,** 118 mg) was dissolved in cyclohexane 50 mL and kept at room temperature (29 ± 2 °C) and monitored daily by TLC (mobile phase, CH_2_Cl_2_; visualizing agent, anisaldehyde-sulfuric acid reagent) for 2 weeks. During the initial period a variable pattern of spots was observed with some spots being transient while others were more long-lasting. A stable TLC pattern of spots was obtained towards the end of the 2-weeks period. It was observed that **3** has been converted into at least six different compounds. The total reaction mixture was chromatographed over a column of dry silica gel (15 g) sequentially eluting with *n*-hexane (200 mL), *n*-hexane:CH_2_Cl_2_ (95:5) (200 mL), *n*-hexane: CH_2_Cl_2_ (90: 10) (200 mL), *n*-hexane:CH_2_Cl_2_ (80:20) (500 mL), *n*-hexane:CH_2_Cl_2_ (75:25) (600 mL), *n*-hexane:CH_2_Cl_2_ (70:30) (200 mL), *n*-hexane:CH_2_Cl_2_ (60:40) (200 mL), *n*-hexane:CH_2_Cl_2_ (50:50) (200 mL), *n*-hexane:CH_2_Cl_2_ (40:60) (200 mL), CH_2_Cl_2_ (300 mL), CH_2_Cl_2_: EtOAc (90:10) (300 mL), and CH_2_Cl_2_: EtOAc (80:20) (200 mL). A total of 453 fractions (F_1_–F_453_) were collected (F_1_–F_132_, 10 mL each and F_133_–F _453_, 5 mL each). The fractions were analyzed by TLC and similar fractions were combined and the solvents were evaporated under reduced pressure. F_420_–F_452_ gave **4** (25.5 mg, 19.5%), F_163_ – F_212_ gave **3** (32.9 mg 26.5%) and F_85_–F_96_ gave **7** (4.5 mg, 3.7%). F_358_–F_389_ gave **6** (9.5 mg, 7.5%) which was found to be a mixture of epimers, **6a** and **6b** by the analysis of NMR spectroscopic data. This mixture of epimers (8.0 mg) was separated by reverse phase TLC using MeOH: H_2_O (9:1) as the eluent (double development, path length 20 cm) to obtain **6a** (2.0 mg) and **6b** (3.0 mg). Fractions F_105_– F_132_ gave a colorless oily mass (7.0 mg) which gave **8** (3.2 mg, 2.2%) and **9** (1.7 mg 1.5%) on separation by preparative TLC using CH_2_Cl_2_ as the eluent (double development, path length 20 cm).

### 15,16-epoxy-*ent*-beyeran-19-oic acid (4)

A colorless viscous liquid; [α]_D_^25^–24° (*c* 0.00525, CHCl_3_); IR (FT-ATR) ν_max_ 2946, 2849, 1693, 1454, 1257, 847 cm^-^1; ^1^H and ^13^C NMR spectroscopic data, see Tables 1 and 2; HRESIMS *m*/*z*, 319.22638 [M + H]^+^ (calculated for C_20_H_31_O_3_, 319.22746).

### *Ent*-beyer-15-en-19-oic acid (5)

A colorless viscous liquid; [α]_D_^25^ +10.9° (*c* 0.0044, CHCl_3_); IR (FT-ATR) ν_max_ 2945, 2846, 1693, 1451, 1255, 753 cm^−1^; ^1^H and ^13^C NMR spectroscopic data, see Tables 1 and 2; GC–MS (*t*_R_ 20.77 min), *m*/*z* 302.2, M^+^.

### 15,16-epoxy-19-*nor*-*ent*-beyeran-4*α*-hydroperoxide (6a)

A colorless viscous liquid; [α]_D_^25^ +72.7° (*c* 0.00165, CHCl_3_); IR (FT-ATR) ν_max_ 3357. 2925, 2868, 2860, 1725, 1455, 1382, 992, 81,846,820,752, 497 cm^−1^; ^1^H and ^13^C NMR spectroscopic data, see Tables 1 and 2. HRESIMS *m*/*z* 307.22594 [M + H]^+^ (calcd. for C_19_H_31_O_3_, 307.22746), *m*/*z* 305.21215 [M–H]^−^ (calcd. for C_19_H_29_O_3_, 305.21180).

### 15,16-epoxy-19-*nor*-*ent*-beyeran-4*β*-hydroperoxide (6b)

Colorless viscous liquid; [α]_D_^25^ + 66.7 (*c* 0.0009, CHCl_3_); IR (FT-ATR) ν_max_ 3316, 2945, 2926, 2850, 1729, 1455, 1370, 996, 882, 847, 814, 747, 498 cm^−1^; ^1^H and ^13^C NMR spectroscopic data, see Tables 1 and 2. HRESIMS, *m*/*z* 307.22617 [M + H]^+^ (calcd. for C_19_H_31_O_3_, 307.22746), *m*/*z* 305.21217 [M–H]^−^ (calcd. for C_19_H_29_O_3_, 305.21180).

### Ent-beyer-19-*nor*-15-en-4*α*-hydroperoixde (7)

A colorless viscous liquid; [α]_D_^25^ +21.82° (*c* 0.00275, CHCl_3_); IR (FT-ATR) ν_max_ 3330, 2922, 2846, 1451, 1365, 1187, 749 cm^−1^; ^1^H and ^13^C NMR spectroscopic data, see Tables 1 and 2. HRESIMS, *m*/*z* 289. 21728 [M–H]^¯^ (calculated for C_19_H_29_O_2_, 289.21689).

### *Ent*-beyer-18-*nor*-15-en-4*β*-hydroperoixde (8)

A colorless viscous; [α]_D_^25^ +50.53° (*c* 0.00095, CHCl_3_); IR (FT-ATR) ν_max_ 3393, 2924, 2848, 1452, 1383, 1187, 751 cm^−1^; ^1^H and ^13^C NMR spectroscopic data, see Tables 1 and 2. HRESIMS, *m*/*z* 289. 21732 [M–H]^¯^ (calculated for C_19_H_29_O_2_, 289.21689).

### *Ent*-beyer-18-*nor*-15-en-4*β*-ol (9)

A colorless viscous liquid; [α]_D_^25^ +53.33° (*c* 0.00075, CHCl_3_); IR (FT-ATR) ν_max_ 3376, 2923, 2850, 1740, 1454, 1383, 1364, 1187, 751 cm^−1^; ^1^H and ^13^C NMR spectroscopic data, (Additional file 2: Table S2). HRESIMS, *m*/*z* 257. 22601 [M + H–H_2_O]^+^ (calculated for C_19_H_30_O, 257.22707).

### Reduction of *Ent*-beyer-15-en-19-al (3) to Erythroxylol A (2)

*Ent*-beyer-15-en-19-al (**3**, 58.5 mg, 0.20 mmol) was dissolved in 25 mL of dry MeOH (dried over molecular sieve-4A) and added excess sodium borohydride. The reaction mixture was kept overnight at room temperature. The reaction mixture was poured into 50 ml of distilled water and acidified with 2 M hydrochloric acid. The acidified reaction mixture was partitioned with CH_2_Cl_2_ (100 ml x 3) and the organic phase was evaporated under reduce pressure to obtain 49 mg of a crude product as white solid which was purified by column chromatography on dry silica eluting with hexane (200 mL) hexane: CH_2_Cl_2_ (95:5) (300 mL) to obtain erythroxylol A (**2**, 30 mg, 51.2%) whose identity was confirmed by comparison with the sample of erythroxylol A isolated by us (TLC, GC-MS).

## Supplementary information

**Additional file 1:** Table giving the composition of essential oil obtained in Stage 1.

**Additional file 2**: **Table S1.** Reported and observed ^13^C NMR data of *ent*-beyer-15-ene (**1**) and erythroxylol A (**2**); **Tables S2** and **S3.** Assignments of spectroscopic data and selected HMBCs of compounds **5** and **9** respectively; **Figures S4–S58.**^1^H NMR, ^13^C NMR, DEPT135, HSQC, and HMBC spectra of compounds **4**–**9** and 1D Selective NOESY Gradient spectra of compounds **4**, **6a**, **6b**, **7**–**9**.

## Data Availability

The data supporting the conclusions of this article is included within the article and its additional files.

## Abbreviations

BHT: Butylated hydroxy toluene
DEPT: Distortionless enhancement by polarization transfer
GC–MS: Gas chromatography-mass spectrometry
HMBC: Heteronuclear multiple bond correlation
HRESIMS: High resolution electro-spray ionization mass spectrometry
HSQC: Heteronuclear single quantum coherence
NOESY: Nuclear overhauser effect spectroscopy
TLC: Thin layer chromatography
