# Supplementary material for: Auto-oxidation of Ent-beyer-15-en-19-al isolated from the essential oil of the heartwood of Erythroxylum monogynum Roxb.: formation of 15,16-epoxy-ent-beyeran-19-oic acid and other products
Source: BMC Chem. 2020 Mar 13;14(1):18. doi: 10.1186/s13065-020-00671-9 (PMC7071572; doi:10.1186/s13065-020-00671-9)
Supplement: Supplementary file 1 — Additional file 1: Table giving the composition of essential oil obtained in Stage 1. [file 13065_2020_671_MOESM1_ESM.pdf]

ADDITIONAL FILE 1

Formation of Epoxy beyeranes during the Auto-oxidation of *Ent*-beyer-15-en-19-al isolated from the Essential oil of the Heartwood of *Erythroxylum monogynum* Roxb.

T. M. Samantha G. Tennakoon[1], G. M. Kamal Bandara Gunaherath[2*], K. Tuley Dayananda De Silva[1], Chayanika Padumadasa[3], D. Siril A. Wijesundara[4], and Ajita Mahendra Abeysekera[3]

[1]Research and Development Laboratory, Link Natural Products Pvt. Ltd. Malinda, Kapugoda, Sri Lanka

[2]Department of Chemistry, Open University of Sri Lanka, P. O. Box 21, Nugegoda, Sri Lanka

[3]Department of Chemistry, University of Sri Jayewardenepura, Nugegoda, Sri Lanka

[4]National Institute of Fundamental Studies, Hantane, Kandy, Sri Lanka

Composition of the Essential Oil (from tage I of the distillation) of the Heartwood of *Erythroxylum monogynum*

Compound*		Relative Peak Area (%)	Retention Index (Calculated)	Retention Index (NIST Database)
1	α-Pinene	53.92	929	937
2	Camphene	1.32	952	952
3	β-Pinene	0.98	979	979
4	β-Myrcene	0.23	991	991
5	*p*- Cymene	0.77	1020	1025
6	Limonene	3.29	1025	1030
7	Fenchyl alcohol	1.74	1110	1115
8	Camphor	0.52	1150	1143
9	Borneol	2.22	1170	1167
10	Terpene-4-ol	0.64	1180	1177
11	α-Terpineol	12.67	1190	1189
12	(-)-Mytenol	0.61	1220	1213
13	*Ent*-beyerene (Stachene)	2.53	1949	1943
14	*Ent*-beyer-15-en-19-al	4.16	2178	#
15	Labd-14-ene-8,13-diol	0.87	2230	2227
16	Erytroxylol-A	6.37	2273	#
	Total	92.84		

*Probability factor for compounds in NIST database > 90%.

#Not listed in the NIST database. Identified by isolation and elucidation of structures.
