# Supplementary material for: Auto-oxidation of Ent-beyer-15-en-19-al isolated from the essential oil of the heartwood of Erythroxylum monogynum Roxb.: formation of 15,16-epoxy-ent-beyeran-19-oic acid and other products
Source: BMC Chem. 2020 Mar 13;14(1):18. doi: 10.1186/s13065-020-00671-9 (PMC7071572; doi:10.1186/s13065-020-00671-9)

ADDITIONAL FILE 2

Formation of Epoxy beyeranes during the Auto-oxidation of *Ent*-beyer-15-en-19-al isolated from the Essential oil of the Heartwood of *Erythroxylum monogynum* Roxb.

T. M. Samantha G. Tennakoon[1], G. M. Kamal Bandara Gunaherath[2*], K. Tuley Dayananda De Silva[1], Chayanika Padumadasa[3], D. Siril A. Wijesundara[4], and Ajita Mahendra Abeysekera[3]

[1]Research and Development Laboratory, Link Natural Products Pvt. Ltd. Malinda, Kapugoda, Sri Lanka

[2]Department of Chemistry, Open University of Sri Lanka, P. O. Box 21, Nugegoda, Sri Lanka

[3]Department of Chemistry, University of Sri Jayewardenepura, Nugegoda, Sri Lanka

[4]National Institute of Fundamental Studies, Hantane, Kandy, Sri Lanka

Table of Content

Table S1. Comparison of reported and observed ^{13}C NMR spectroscopic data of *ent*-beyer-15-ene (**1**) and erythroxylol A (**2**)

Table S2. Comparison of reported ^{1}H (200 MHz) and ^{13}C (50 MHz) NMR Spectroscopic data with observed ^{1}H (400 MHz) and ^{13}C (100 MHz) NMR Spectroscopic Data and Selected HMBC correlations of **5**

Table S3. ^{1}H (400 MHz) and ^{13}C (100 MHz) NMR Spectroscopic Data and Selected HMBC correlations of **9**

For compound 3

Figure S4. ^{1}H NMR Spectrum (400 MHz) of **3** in $CDCl_3$

Figure S5. ^{13}C NMR Spectrum (100 MHz) of **3** in $CDCl_3$

Figure S6. DEPT 135 Spectrum (100 MHz) of **3** in $CDCl_3$

Figure S7. HSQC Spectrum of **3** in $CDCl_3$

Figure S8. HMBC Spectrum of **3** in $CDCl_3$

For compound 4

Figure S9. ^{1}H NMR Spectrum (400 MHz) of **4** in $CDCl_3$

Figure S10. ^{1}H NMR Spectrum (400 MHz) of **4** in $CDCl_3$ (Expansion δ 0.00 – 2.50)

Figure S11. ^{13}C NMR Spectrum (100 MHz) of **4** in $CDCl_3$

Figure S12. DEPT 135 Spectrum (100 MHz) of **4** in $CDCl_3$

Figure S13. HSQC Spectrum of **4** in $CDCl_3$

Figure S14. HMBC Spectrum of **4** in $CDCl_3$

Figure S15. 1D Selective Gradient NOESY spectrum (400 MHz) of **4** in $CDCl_3$; Irradiation at δ 3.433

For compound 5

Figure S16. ^{1}H NMR Spectrum (400 MHz) of **5** in $CDCl_3$

Figure S17. ^{1}H NMR Spectrum (400 MHz) of **5** in $CDCl_3$ (Expansion δ 0.00 – 2.50)

Figure S18. ^{13}C NMR Spectrum (100 MHz) of **5** in $CDCl_3$

Figure S19. DEPT 135 Spectrum (100 MHz) of **5** in $CDCl_3$

Figure S20. HSQC Spectrum of **5** in $CDCl_3$

Figure S21. HMBC Spectrum of **5** in $CDCl_3$

For compound 6a and 6b

Figure S22. ^{1}H NMR Spectrum (400 MHz) of **6a** and **6b** in $CDCl_3$

Figure S23. ^{1}H NMR Spectrum (400 MHz) of **6a** and **6b** in $CDCl_3$ (Expansion δ 0.40 – 2.00)

Figure S24. ^{13}C NMR Spectrum (100 MHz) of **6a** and **6b** in $CDCl_3$

Figure S25. ^{13}C NMR Spectrum (100 MHz) of **6a** and **6b** in $CDCl_3$ (Expansion δ 0.00 – 65.00)

Figure S26. DEPT 135 Spectrum (100 MHz) of **6a** and **6b** in $CDCl_3$

Figure S27. HSQC Spectrum of **6a** and **6b** in $CDCl_3$

Figure S28. HMBC Spectrum of **6a** and **6b** in $CDCl_3$

Figure S29. 1D Selective Gradient NOESY spectrum (400 MHz) of **6a** and **6b** in $CDCl_3$; Irradiation at δ 0.924

Figure S30. 1D Selective Gradient NOESY spectrum (400 MHz) of **6a** and **6b** in $CDCl_3$; Irradiation at δ 1.134

Figure S31. 1D Selective Gradient NOESY spectrum (400 MHz) of **6a** and **6b** in $CDCl_3$; Irradiation at δ 1.296

Figure S32. ^{1}H NMR Spectrum (400 MHz) of **6a** in $CDCl_3$

Figure S33. ^{1}H NMR Spectrum (400 MHz) of **6a** in $CDCl_3$ (Expansion δ 0.40 – 3.50)

Figure S34. ^{13}C NMR Spectrum (100 MHz) of **6a** in $CDCl_3$

Figure S35. ^{1}H NMR Spectrum (400 MHz) of **6b** in $CDCl_3$

Figure S36. ^{1}H NMR Spectrum (400 MHz) of **6b** in $CDCl_3$ (Expansion δ 0.40 – 3.50)

Figure S37. ^{13}C NMR Spectrum (100 MHz) of **6b** in $CDCl_3$

For compound 7

Figure S38. ^{1}H NMR Spectrum (400 MHz) of **7** in $CDCl_3$

Figure S39. ^{13}C NMR Spectrum (100 MHz) of **7**in $CDCl_3$

Figure S40. DEPT 135 Spectrum (100 MHz) of **7** in $CDCl_3$

Figure S41. HSQC Spectrum of **7** in $CDCl_3$

Figure S42. HMBC Spectrum of **7** in $CDCl_3$

Figure S43. 1D Selective Gradient NOESY spectrum (400 MHz) of **7** in $CDCl_3$; Irradiation at δ 0.856

Figure S44. 1D Selective Gradient NOESY spectrum (400 MHz) of **7** in $CDCl_3$; Irradiation at δ 1.282

For compound 8

Figure S45. ^{1}H NMR Spectrum (400 MHz) of **8** in $CDCl_3$

Figure S46. ^{13}C NMR Spectrum (100 MHz) of **8** in $CDCl_3$

Figure S47. DEPT 135 Spectrum (100 MHz) of **8** in $CDCl_3$

Figure S48. HSQC Spectrum of **8** in $CDCl_3$

Figure S49. HMBC Spectrum of **8** in $CDCl_3$

Figure S50. 1D Selective Gradient NOESY spectrum (400 MHz) of **8** in $CDCl_3$; Irradiation at δ 0.740

Figure S51. 1D Selective Gradient NOESY spectrum (400 MHz) of **8** in $CDCl_3$; Irradiation at δ 1.117

For compound 9

Figure S52. ^{1}H NMR Spectrum (400 MHz) of **9** in $CDCl_3$

Figure S53. ^{13}C NMR Spectrum (100 MHz) of **9** in $CDCl_3$

Figure S54. DEPT 135 Spectrum (100 MHz) of **9** in $CDCl_3$

Figure S55. HSQC Spectrum of **9** in $CDCl_3$

Figure S56. HMBC Spectrum of **9** in $CDCl_3$

Figure S57. 1D Selective Gradient NOESY spectrum (400 MHz) of **9** in $CDCl_3$; Irradiation at δ 1.136

Figure S58. 1D Selective Gradient NOESY spectrum (400 MHz) of **9** in $CDCl_3$; Irradiation at δ 0.709

S1. Comparison of reported and observed ^{13}C NMR spectroscopic data of *ent*-beyer-15-ene (**1**) and erythroxylol A (**2**) in $CDCl_3$

	Ent-beyer-15-ene (**1**)		Erythroxylol A (**2**)	
C	*δ* (reported) at 20 MHz	*δ* (observed) at 100 MHz	*δ* (reported) at 20 MHz	*δ* (observed) at 100 MHz
1	39.3	39.3	39.2	39.2
2	18.7	18.7	18.3	18.3
3	42.2	42.2	35.7	35.6
4	33.3	33.2	38.5	38.5
5	56.1	56.1	56.8	56.8
6	20.3	20.2	20.1	20.2
7	37.4	37.36 or 37.41	37.3	37.7 or 37.2
8	49.1	49.1	49.0	49.0
9	53.0	52.9	53.0	52.9
10	37.4	37.36 or 37.41	37.3	37.7 or 37.2
11	20.5	20.3	20.3	20.3
12	33.7	33.3	33.2	33.2
13	43.6	43.7	43.6	43.6
14	61.3	61.3	61.2	61.1
15	135.2	135.4	135.0	135.0
16	136.1	136.3	136.0	136.5
17	25.0	25.1	24.9	24.9
18	33.8	33.8	27.0	27.0
19	22.0	22.1	65.5	65.5
20	15.1	15.1	15.6	15.8

S2. Comparison of reported ^{1}H (200 MHz) and ^{13}C (50 MHz) NMR Spectroscopic data with observed ^{1}H (400 MHz) and ^{13}C (100 MHz) NMR Spectroscopic Data and Selected HMBC correlations of **5**

C	Type	δ_H (Rep)	δ_C (Rep)	δ_H (Obs)	δ_C (Obs)	HMBC
1	CH_2		39.6	0.90 m, 1.69 m	39.5	2
2	CH_2		19.3	1.41 m, 1.80 m	19.2	
3	CH_2		38.0	1.01 m, 2.15 m	37.9	1, 5
4	C		43.9	-	43.8	
5	CH		57.2	1.10 m	57.0	4, 9, 10, 19
6	CH_2		21.6	1.82 m	21.6	5,8
7	CH_2		37.7	1.29 m, 1.66 m	37.7	
8	C		49.2	-	49.1	
9	CH		52.4	0.97 m	52.3	
10	C		37.7	-	37.9	
11	CH_2		20.5	1.53 m, 1.25	20.4	8, 12, 13
12	CH_2		33.2	1.25 m	33.1	
13	C		43.7	-	43.7	
14	CH_2		61.1	1.01 m, 1.45 m	61.0	7, 9
15	CH	5.47 d (*J*=5.7 Hz)	134.8	5.73 d (*J*=5.7 Hz)	134.8	8, 14
16	CH	5.76 d (*J*=5.7 Hz)	136.5	5.45 d (*J*=5.7 Hz)	136.5	8, 13, 14, 17
17	CH_3	1.01 s	24.9	0.99 s	24.9	16, 12, 13
18	CH_3	1.26 s	29.1	1.24 s	29.1	3, 4, 5, 19
19	C		184.3	-	183.8	
20	CH_3	0.69 s	13.8	0.67 s	13.8	1, 5, 9, 10

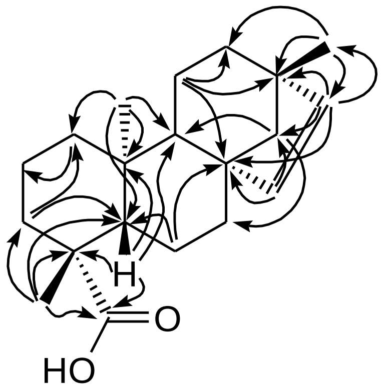

S3. ^{1}H (400 MHz) and ^{13}C (100 MHz) NMR Spectroscopic Data and Selected HMBC correlations of **9**

C	δ_H	δ_C		HMBC
1	0.89 m, 1.57 m	38.4	CH_2	
2	1.51 m	19.9	CH_2	
3	1.33 m, 1.76 m	43.0	CH_2	2
4	-	72.4	C	
5	1.13 m	57.6	CH	
6	1.38 m, 1.75 m	19.1	CH_2	
7	1.37 m, 1.66 m	36.7	CH_2	5, 6
8	-	49.0	C	
9	1.05 m	52.6	CH	
10	-	38.0	C	
11	0.83 m, 1.27 m	20.4	CH_2	13
12	1.27 m	33.1	CH_2	14
13	-	43.7	C	
14	1.04 m, 1.45 m	61.1	CH_2	8, 9, 12, 13
15	5.68 d (*J*= 5.7 Hz)	135.0	CH	8, 13, 14, 16
16	5.46 d (*J*= 5.7 Hz)	136.5	CH	8, 13, 14, 15
17	1.00 s	24.9	CH_3	12, 13, 14, 16
18	-	-		
19	1.14 s	23.3	CH_3	3, 4, 5,
20	0.71 s	14.5	CH_3	1, 5, 9, 10

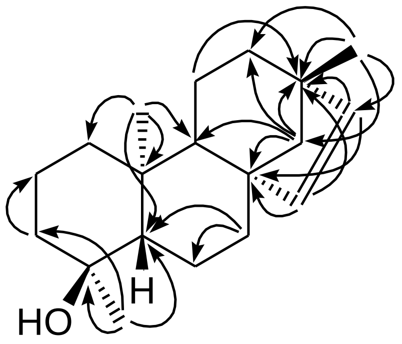

S4. ^{1}H NMR Spectrum (400 MHz) of **3** in $CDCl_3$

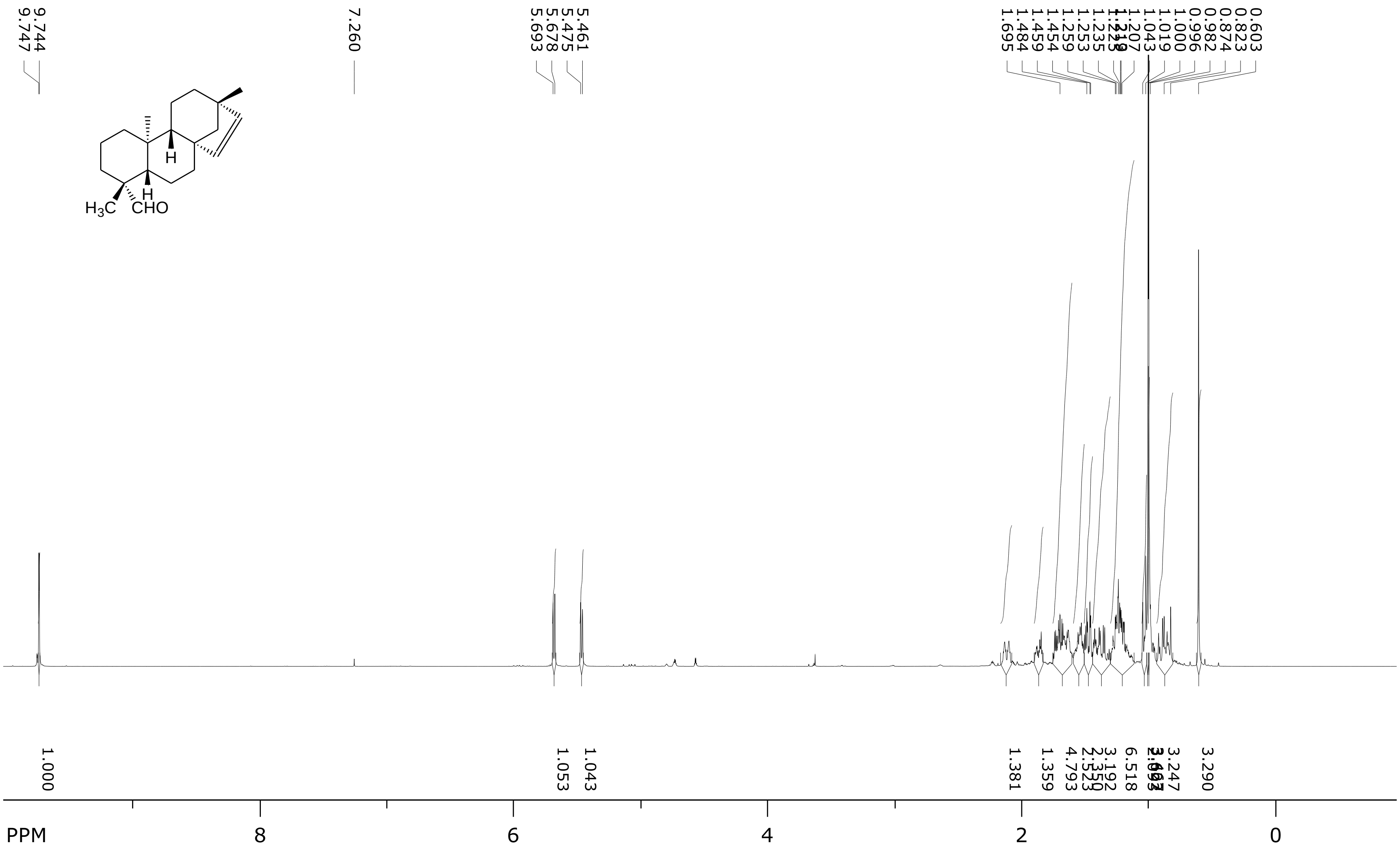

S5. ^{13}C NMR Spectrum (100 MHz) of **3** in $CDCl_3$

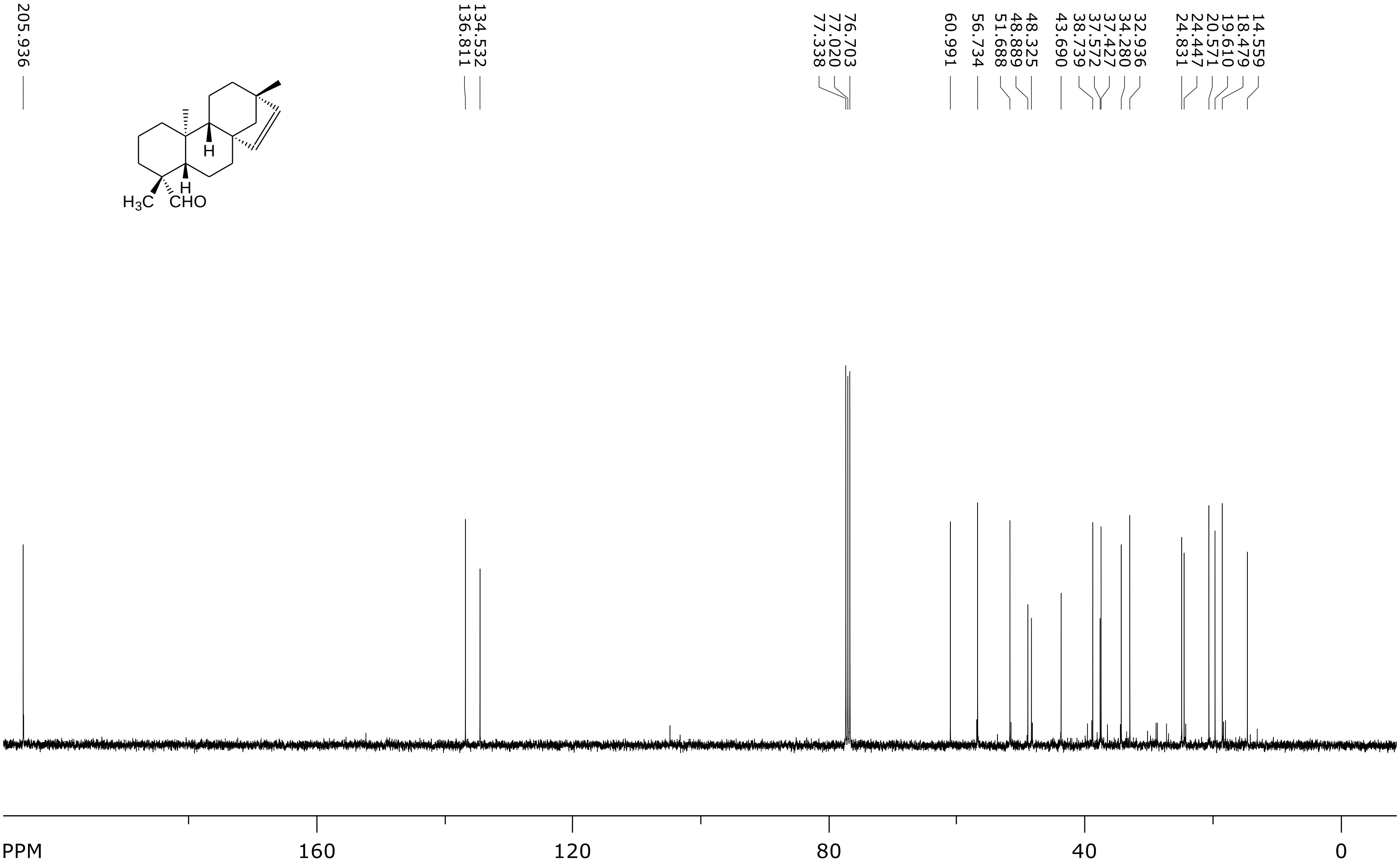

S6. DEPT 135 Spectrum (100 MHz) of **3** in $CDCl_3$

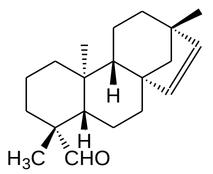

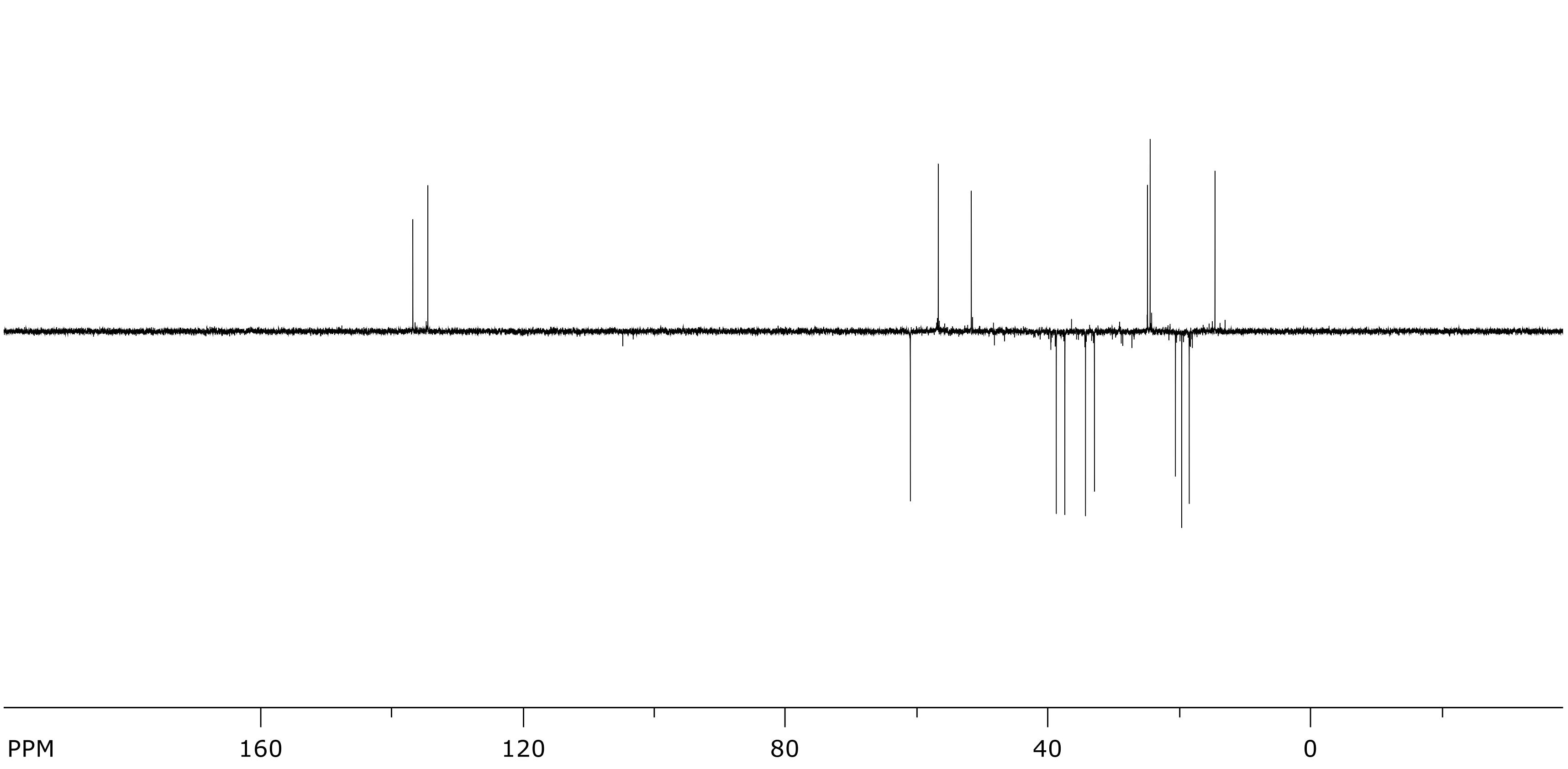

S7. HSQC Spectrum of **3** in $CDCl_3$

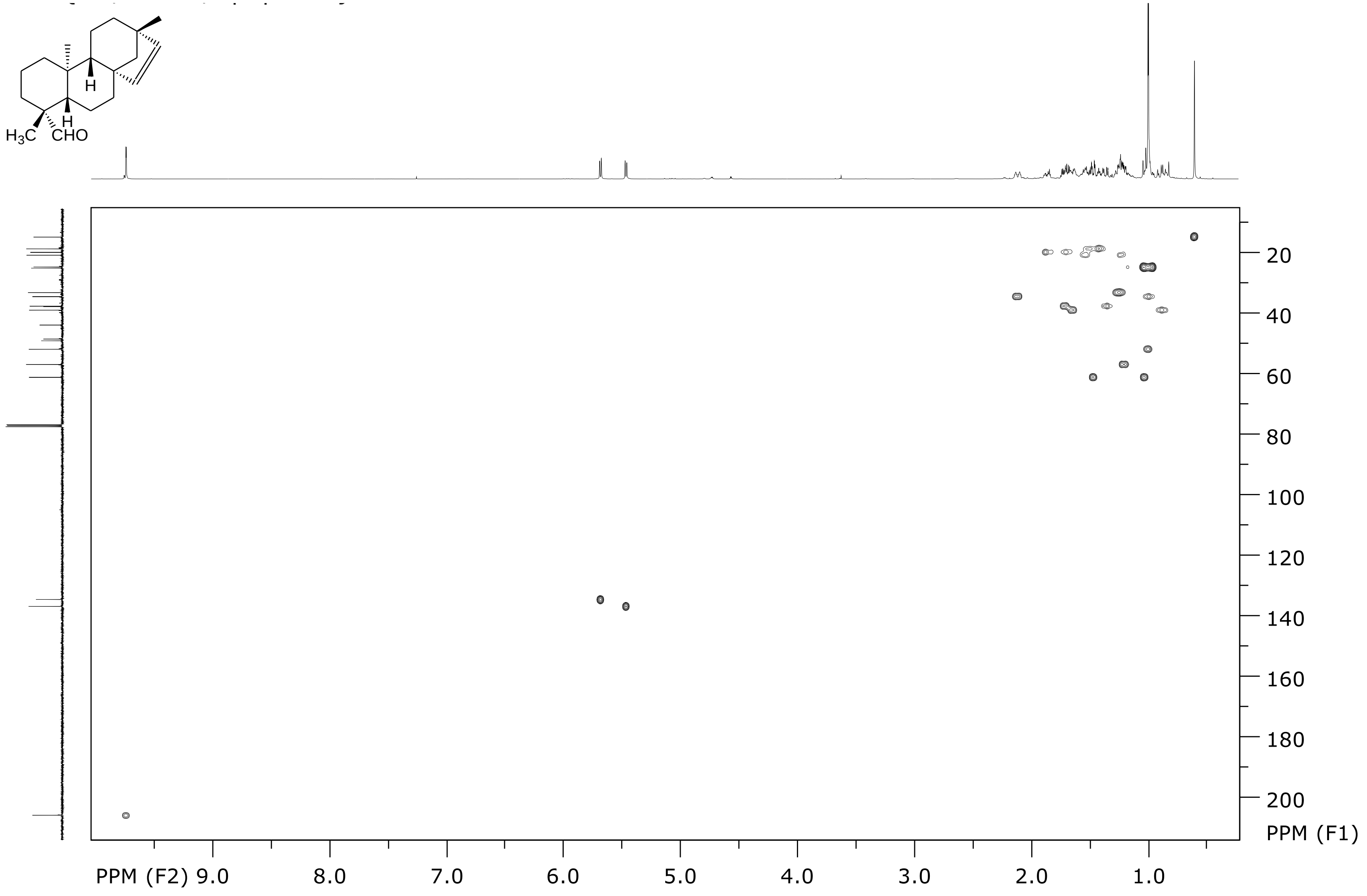

S8. HMBC Spectrum of **3** in $CDCl_3$

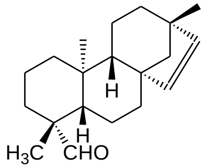

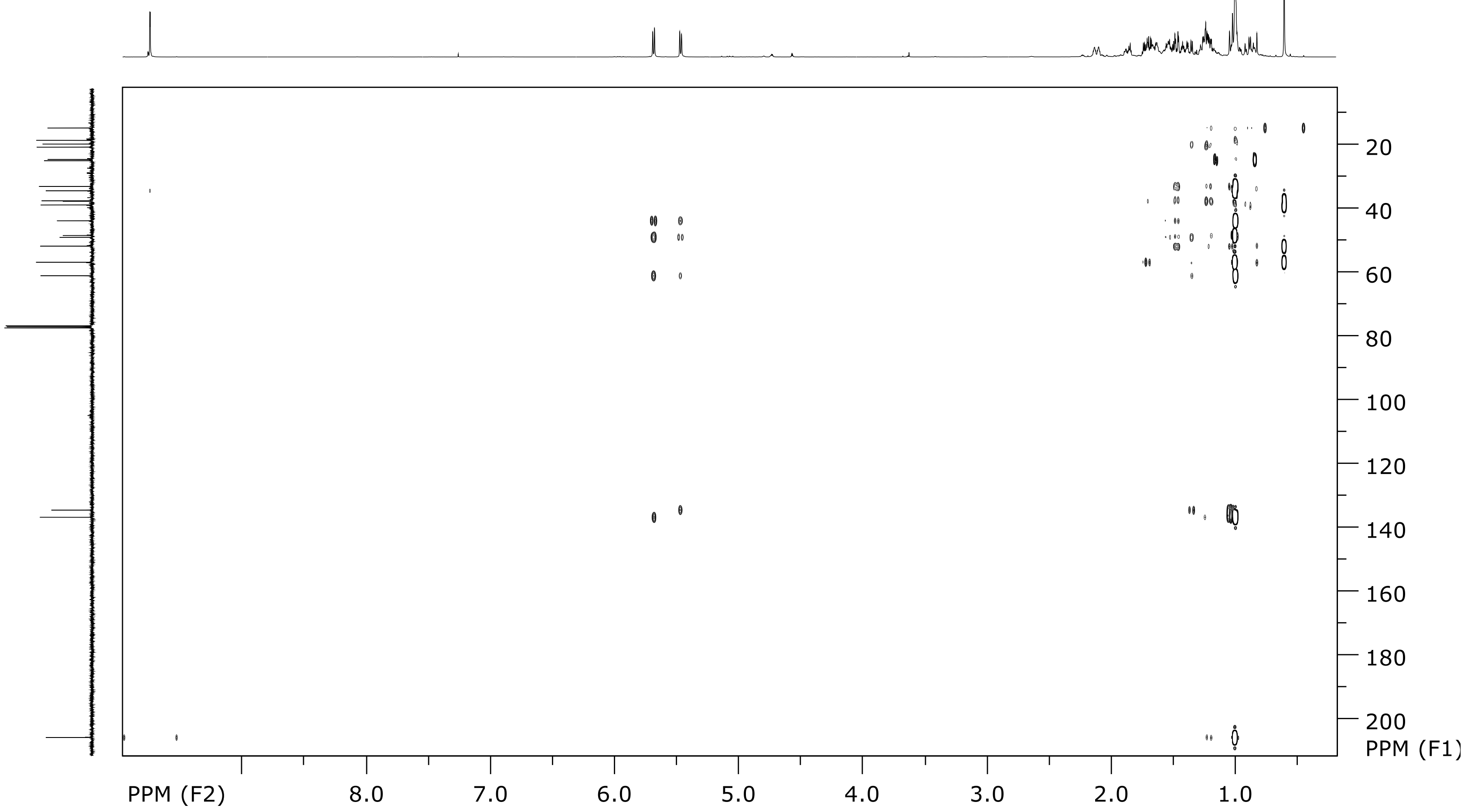

S9. ^{1}H NMR Spectrum (400 MHz) of **4** in $CDCl_3$

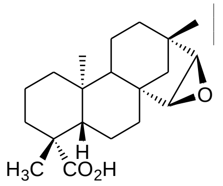

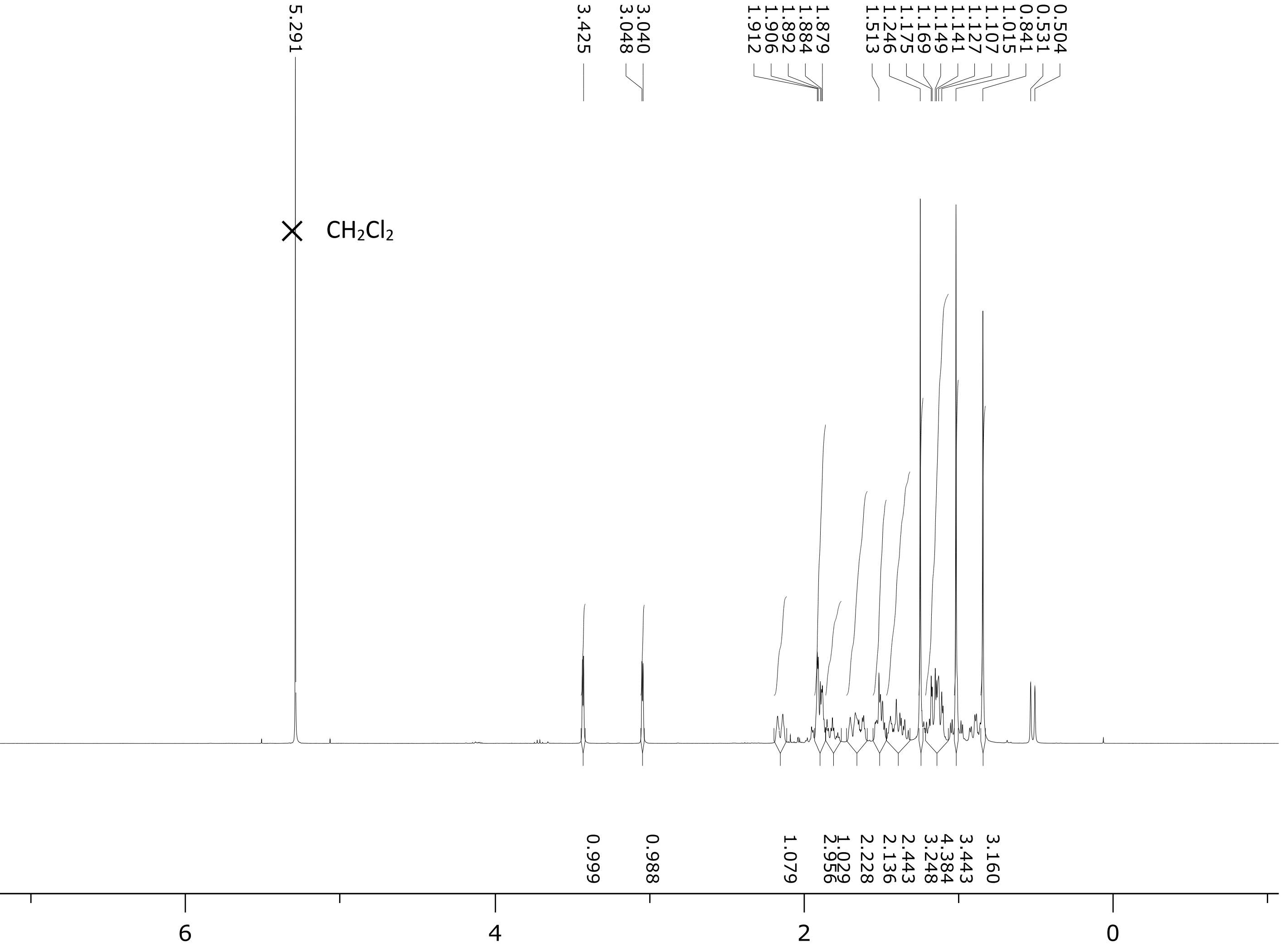

S10. ^{1}H NMR Spectrum (400 MHz) of **4** in $CDCl_3$ (Expansion δ 0.00 – 2.50)

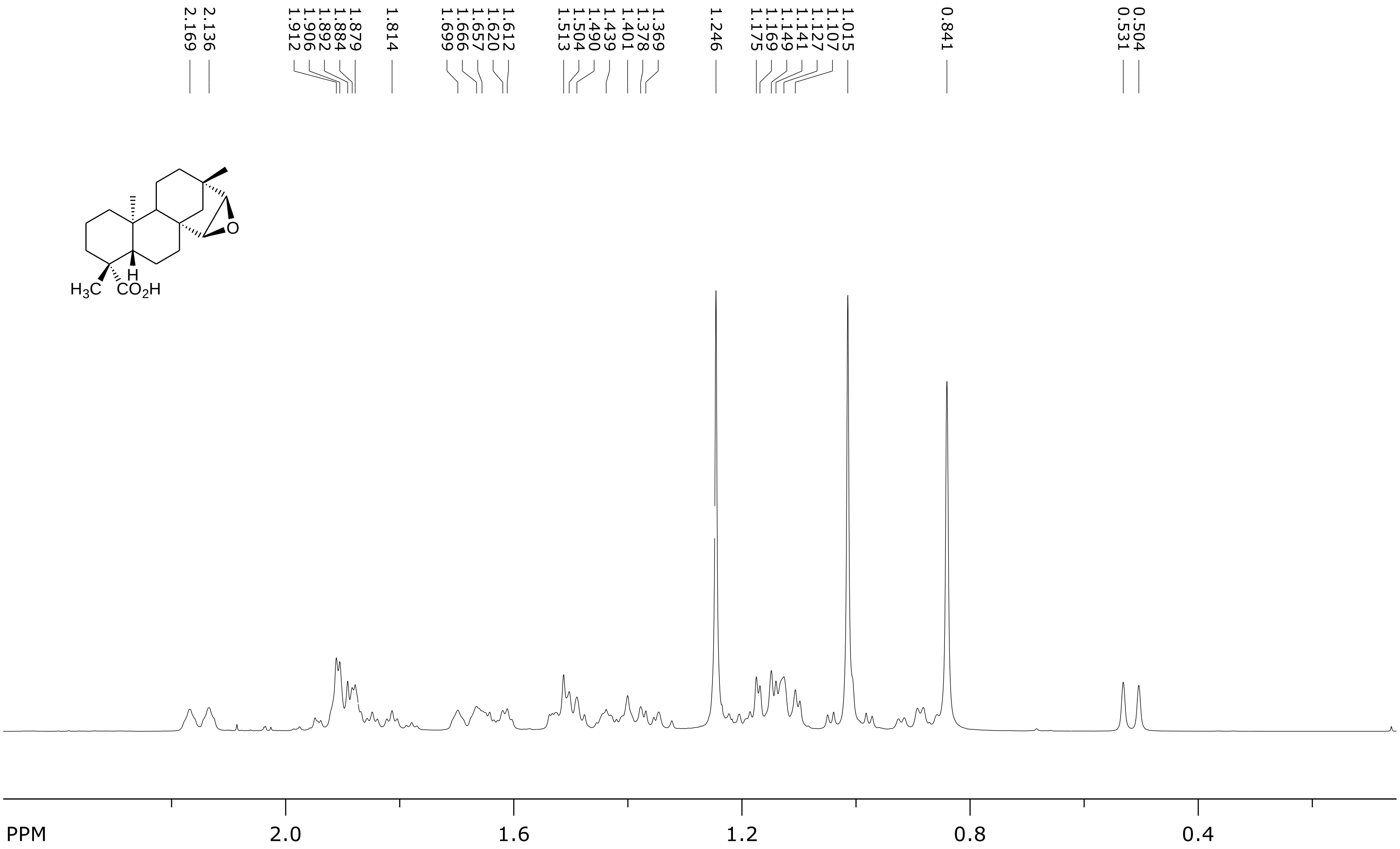

S11. ^{13}C NMR Spectrum (100 MHz) of **4** in $CDCl_3$

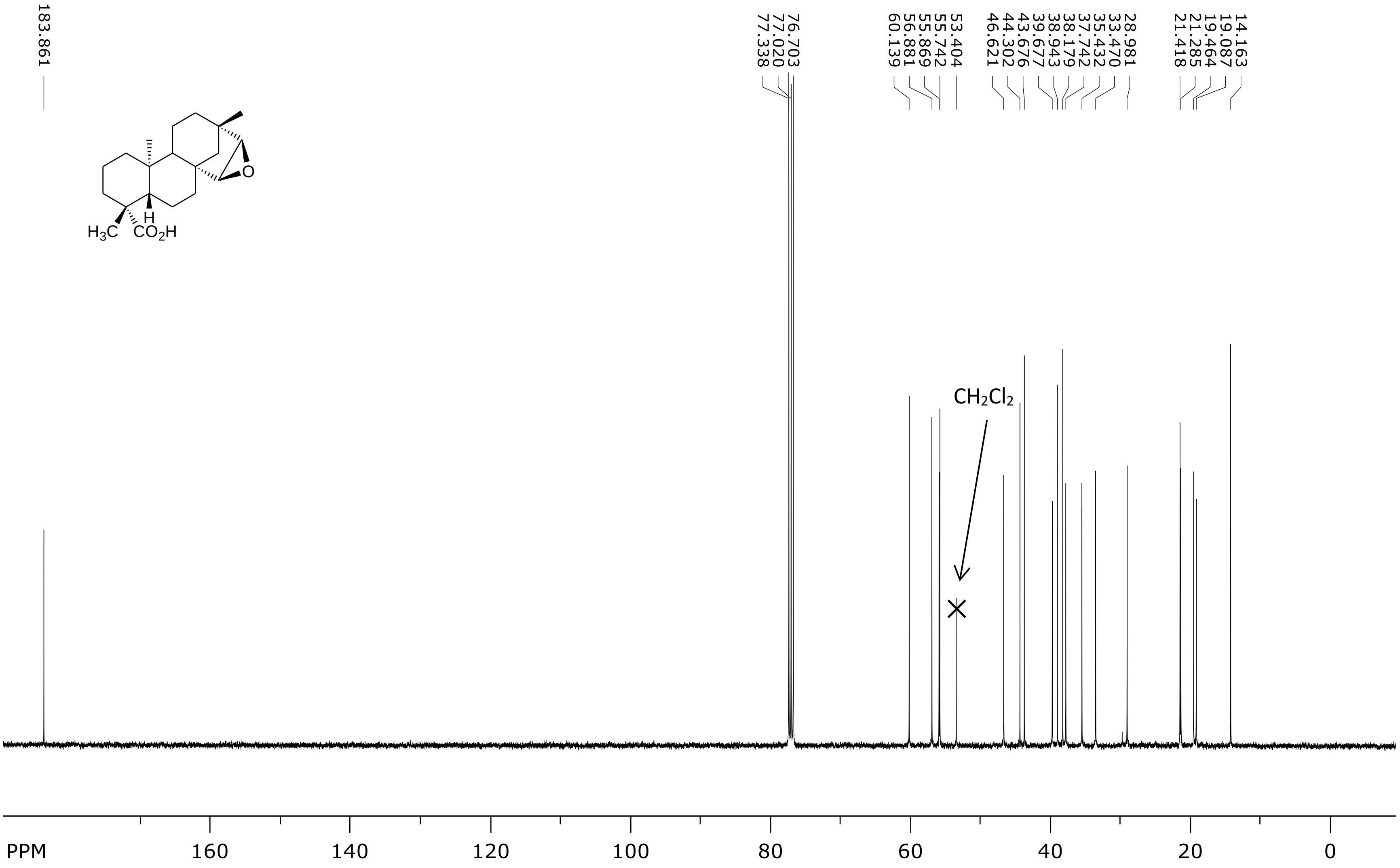

S12. DEPT135 Spectrum (100 MHz) of **4** in $CDCl_3$

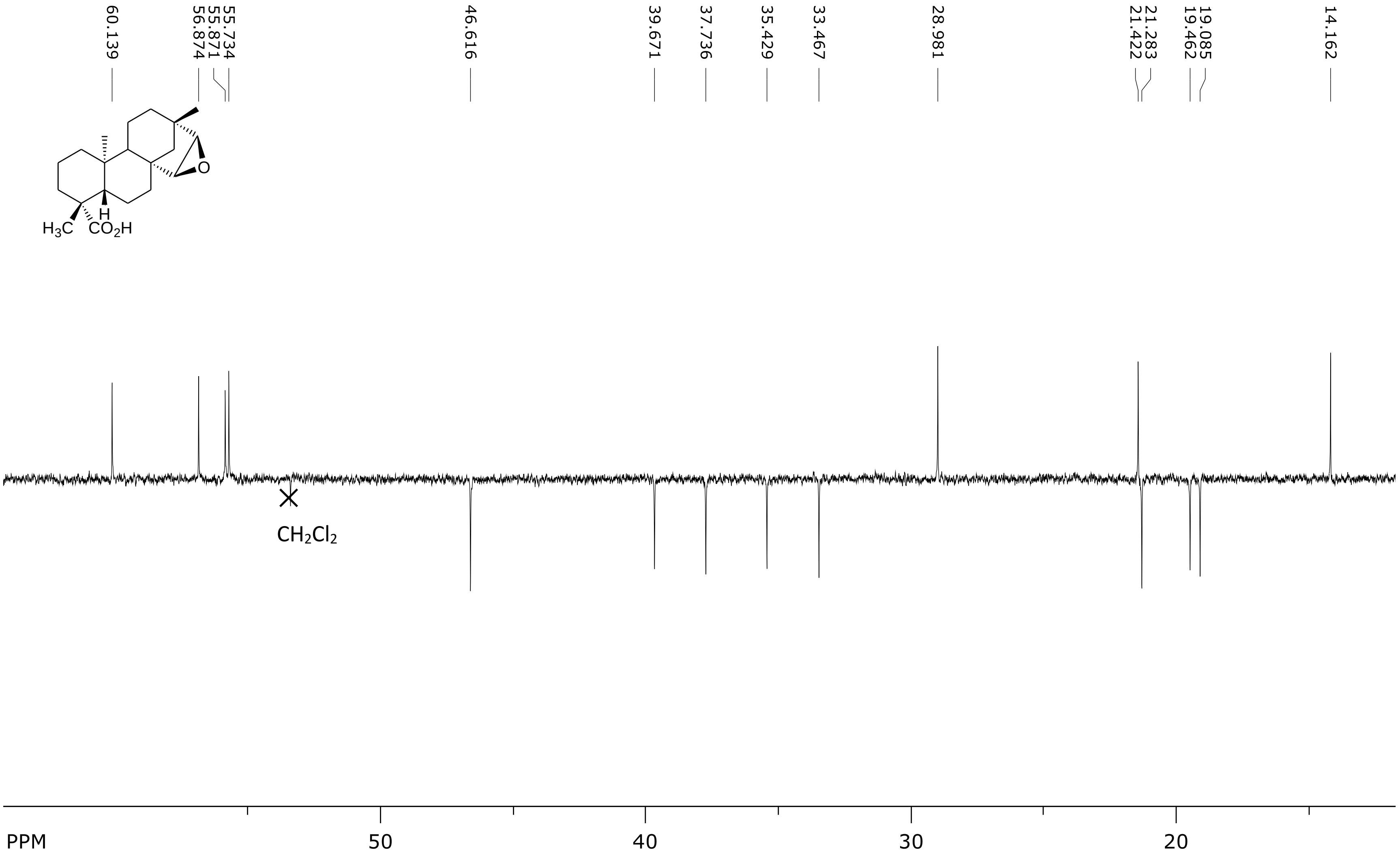

S13. HSQC spectrum of **4** in $CDCl_3$

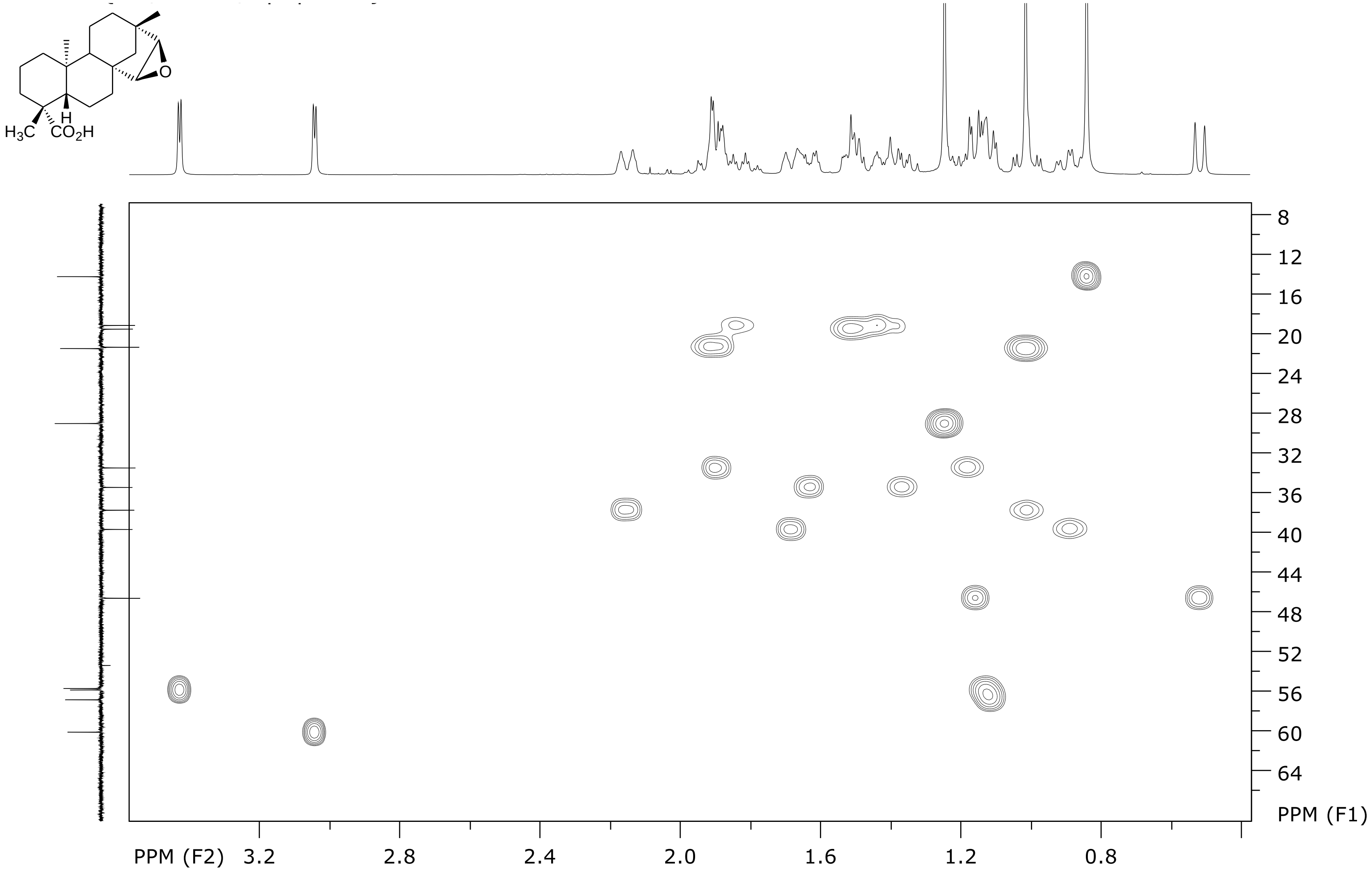

S14. HMBC spectrum of **4** in $CDCl_3$

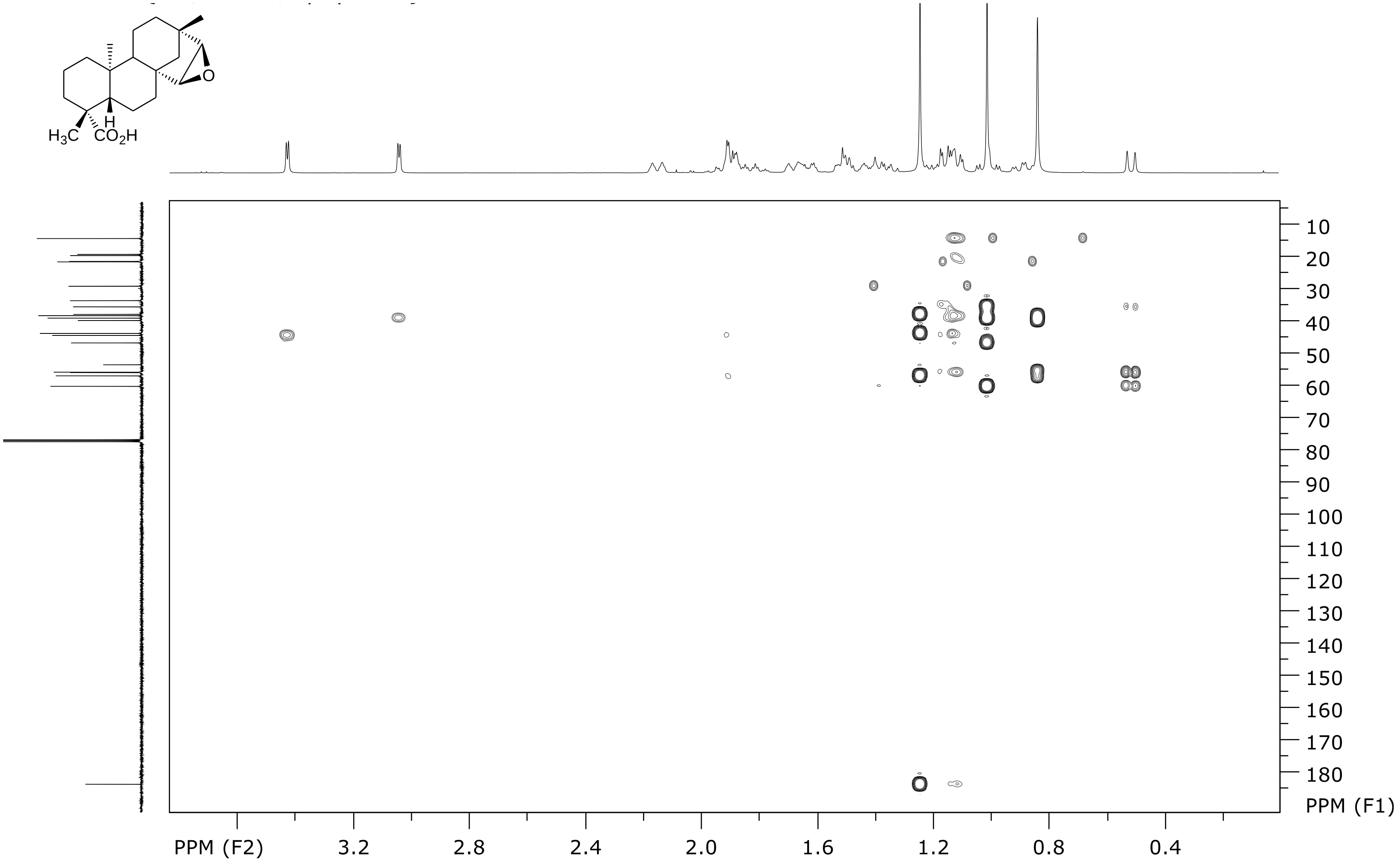

S15. 1D Selective Gradient NOESY spectrum (400 MHz) of **4** in $CDCl_3$; Irradiation at δ 3.433

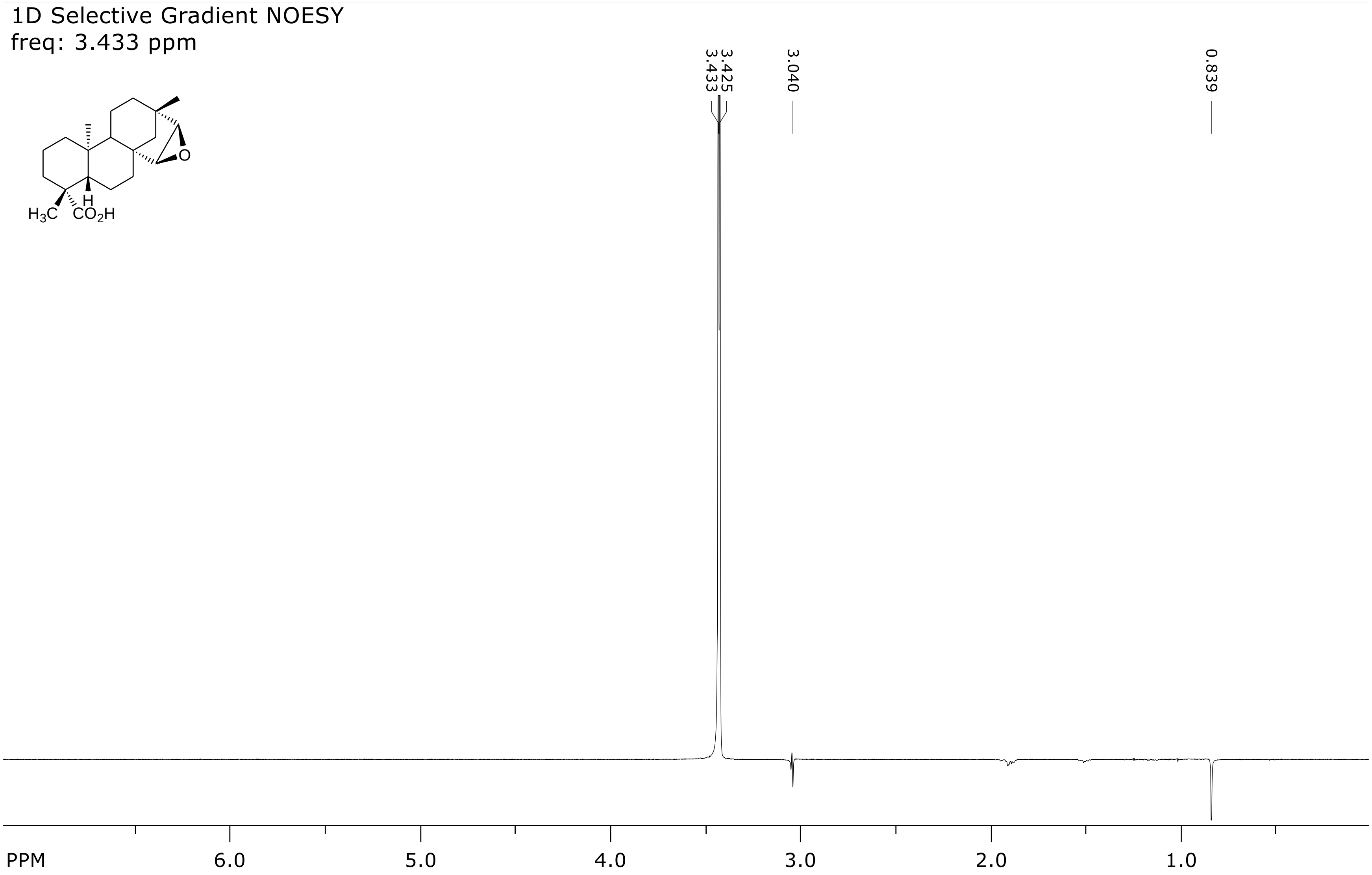

S16. ¹H NMR Spectrum (400 MHz) of **5** in $CDCl_3$

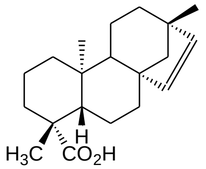

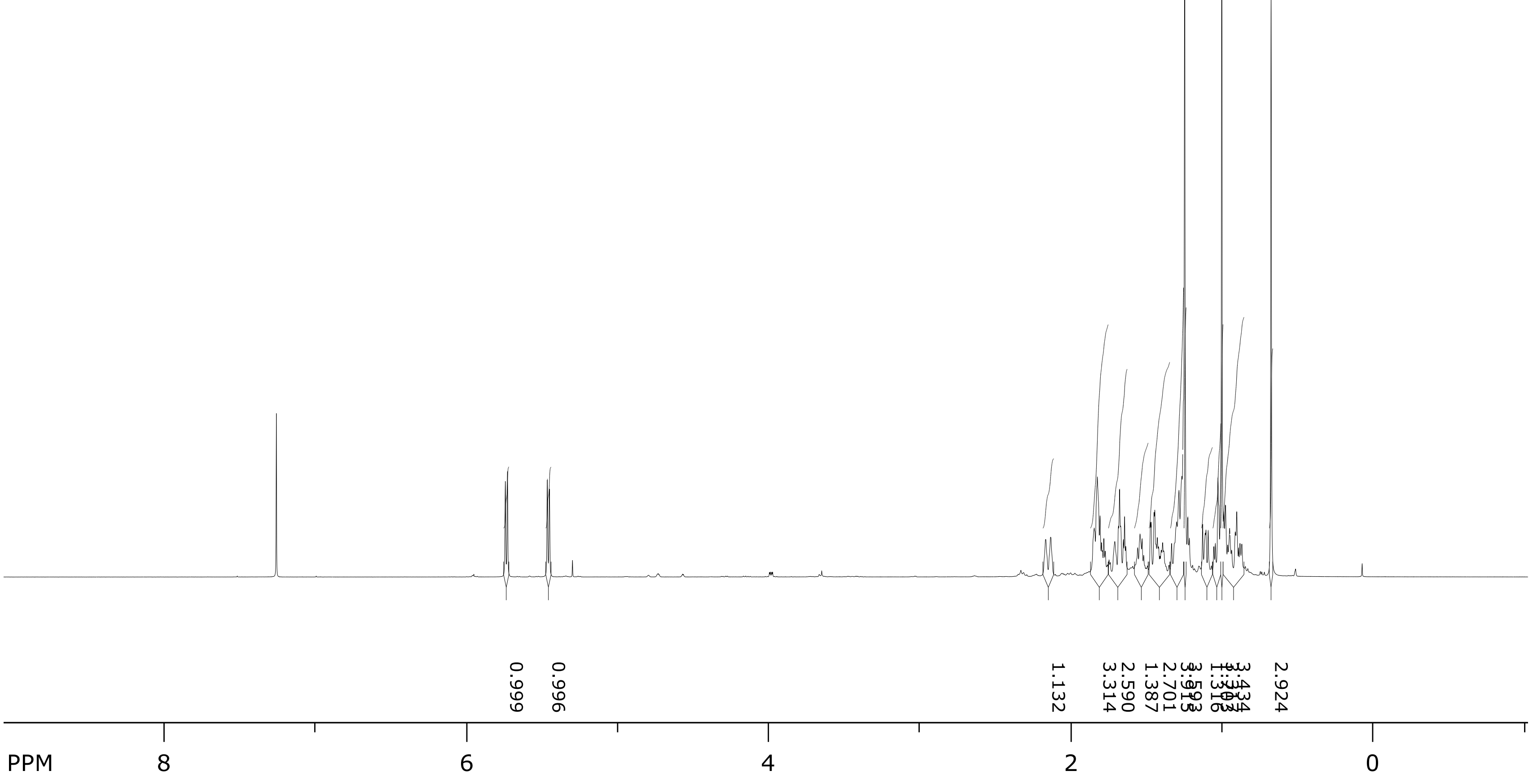

S17. ^{1}H NMR Spectrum (400 MHz) of **5** in $CDCl_3$ (Expansion δ 0.00 – 2.50)

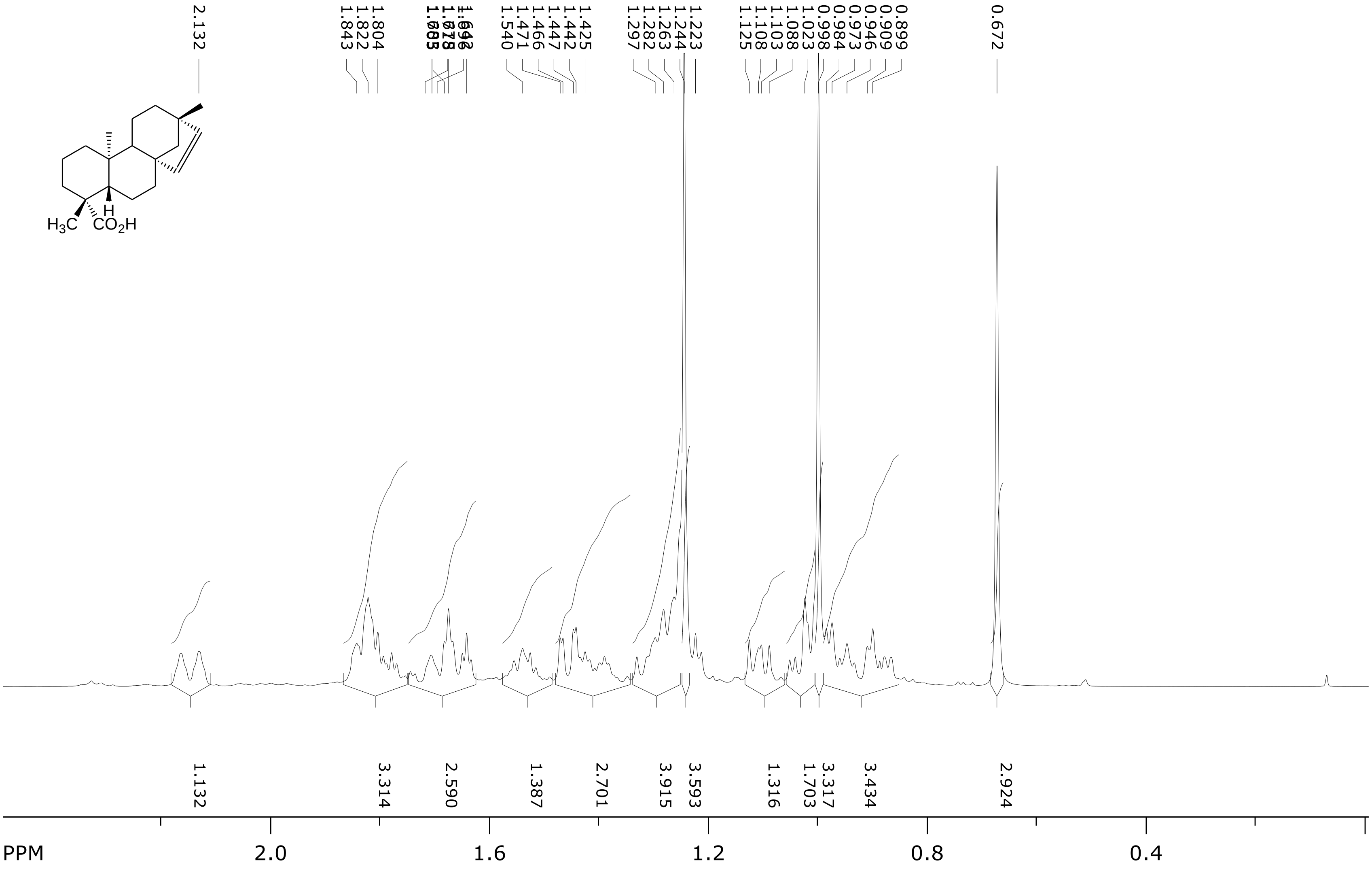

S18. ^{13}C NMR Spectrum (100 MHz) of **5** in $CDCl_3$

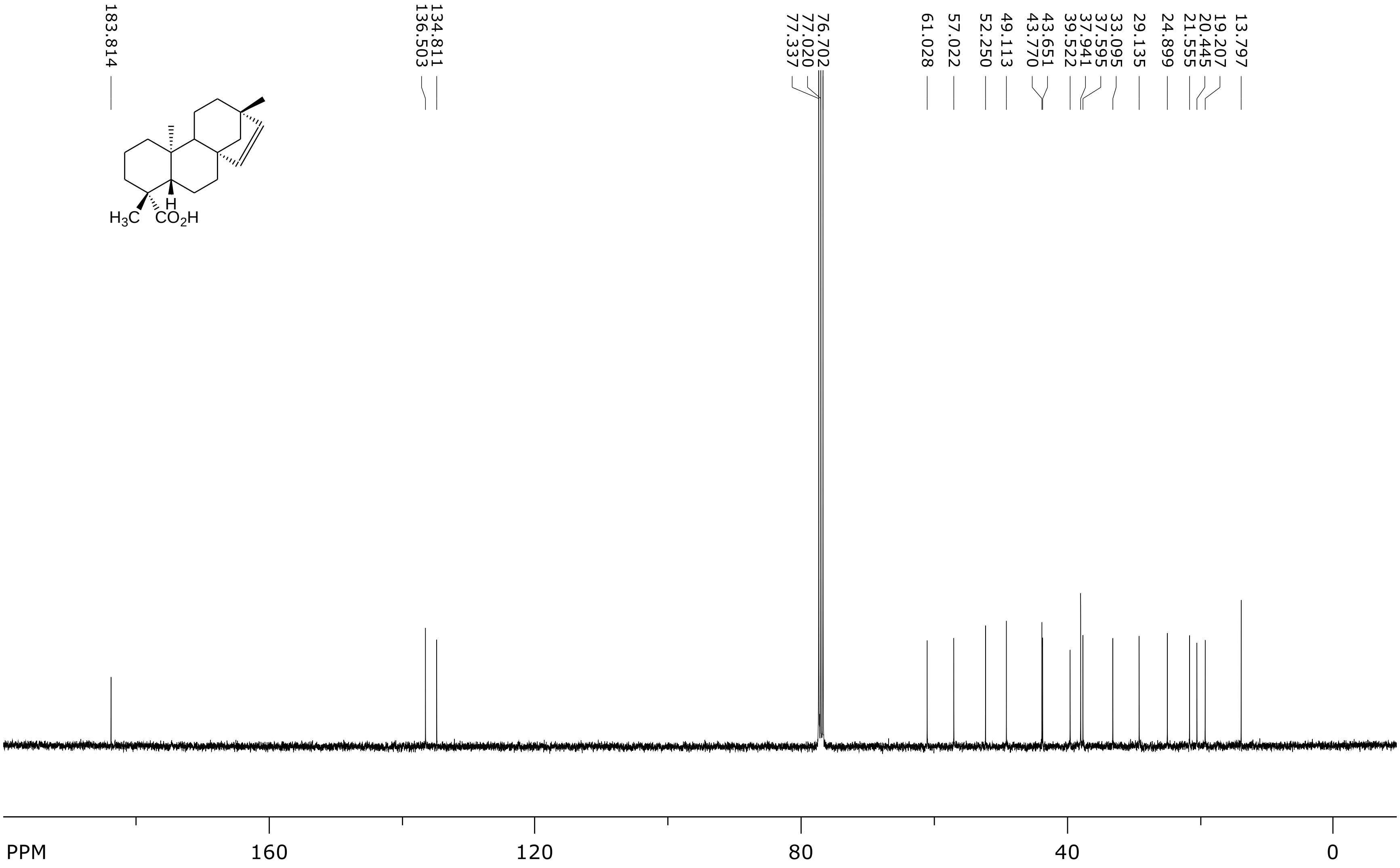

S19. DEPT 135 Spectrum (100 MHz) of **5** in $CDCl_3$

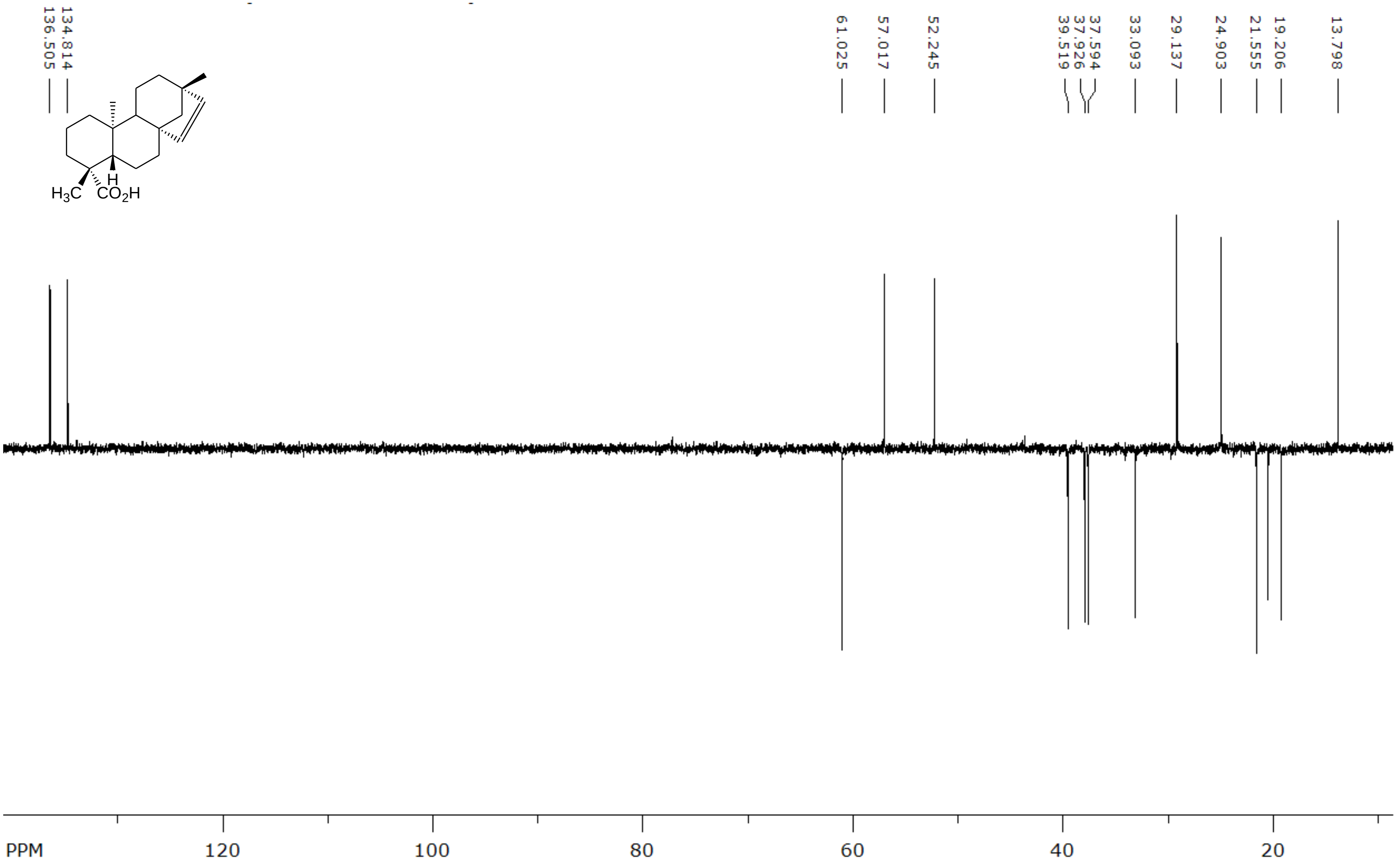

S20. HSQC Spectrum of **5** in $CDCl_3$

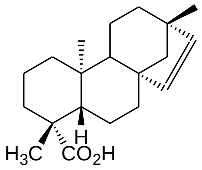

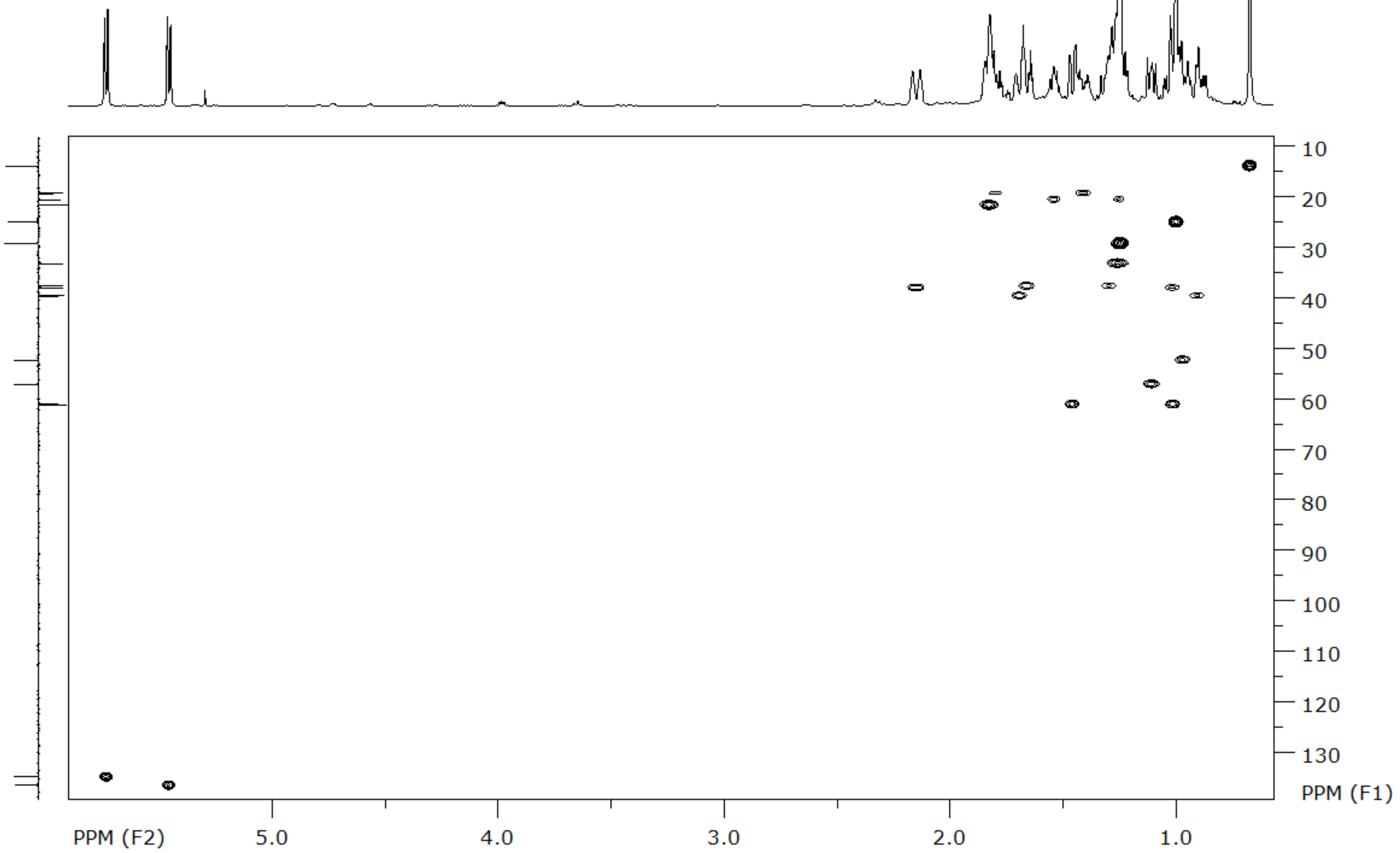

S21. HMBC Spectrum of **5** in $CDCl_3$

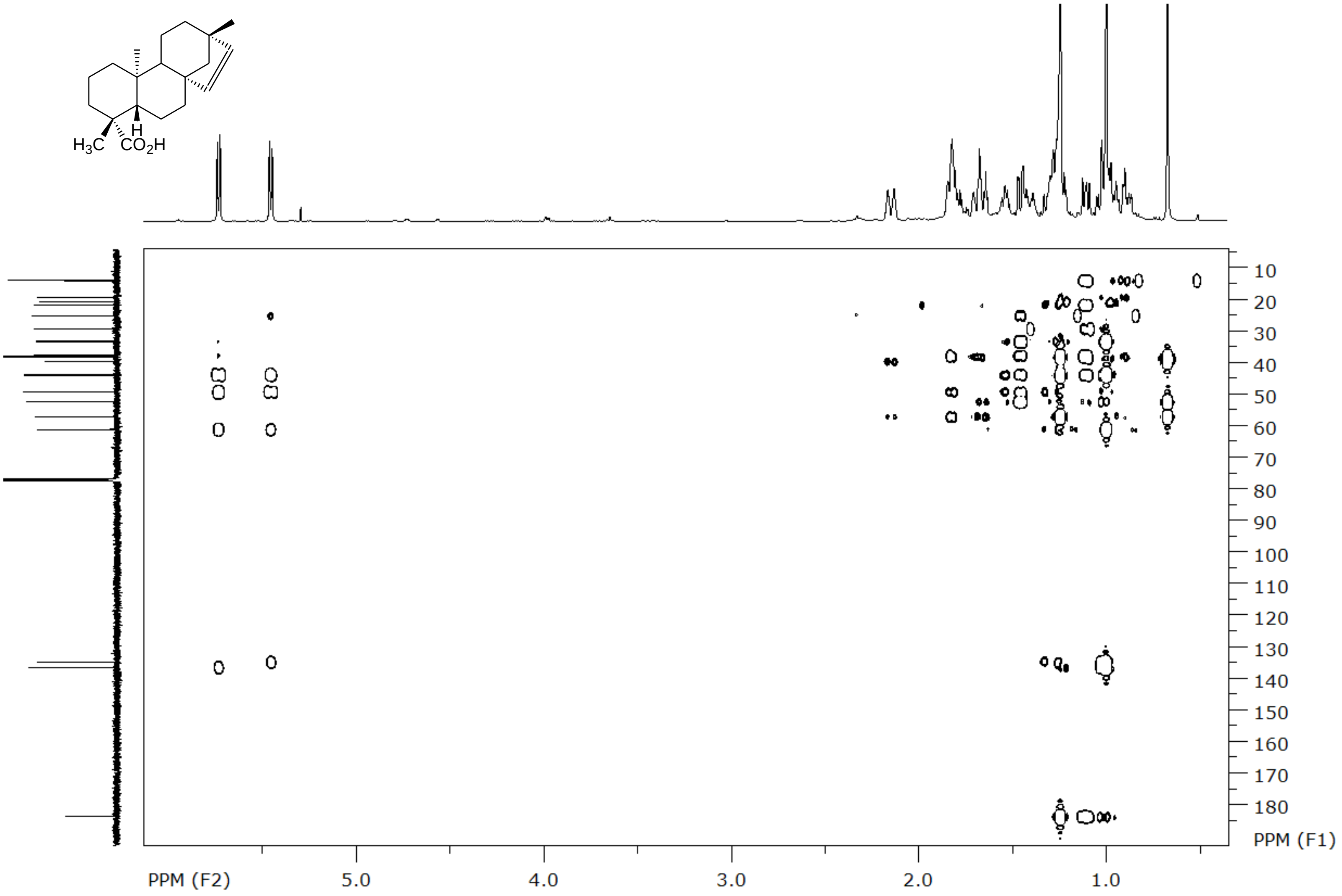

S22. ^{1}H NMR Spectrum (400 MHz) of **6a** and **6b** in $CDCl_3$

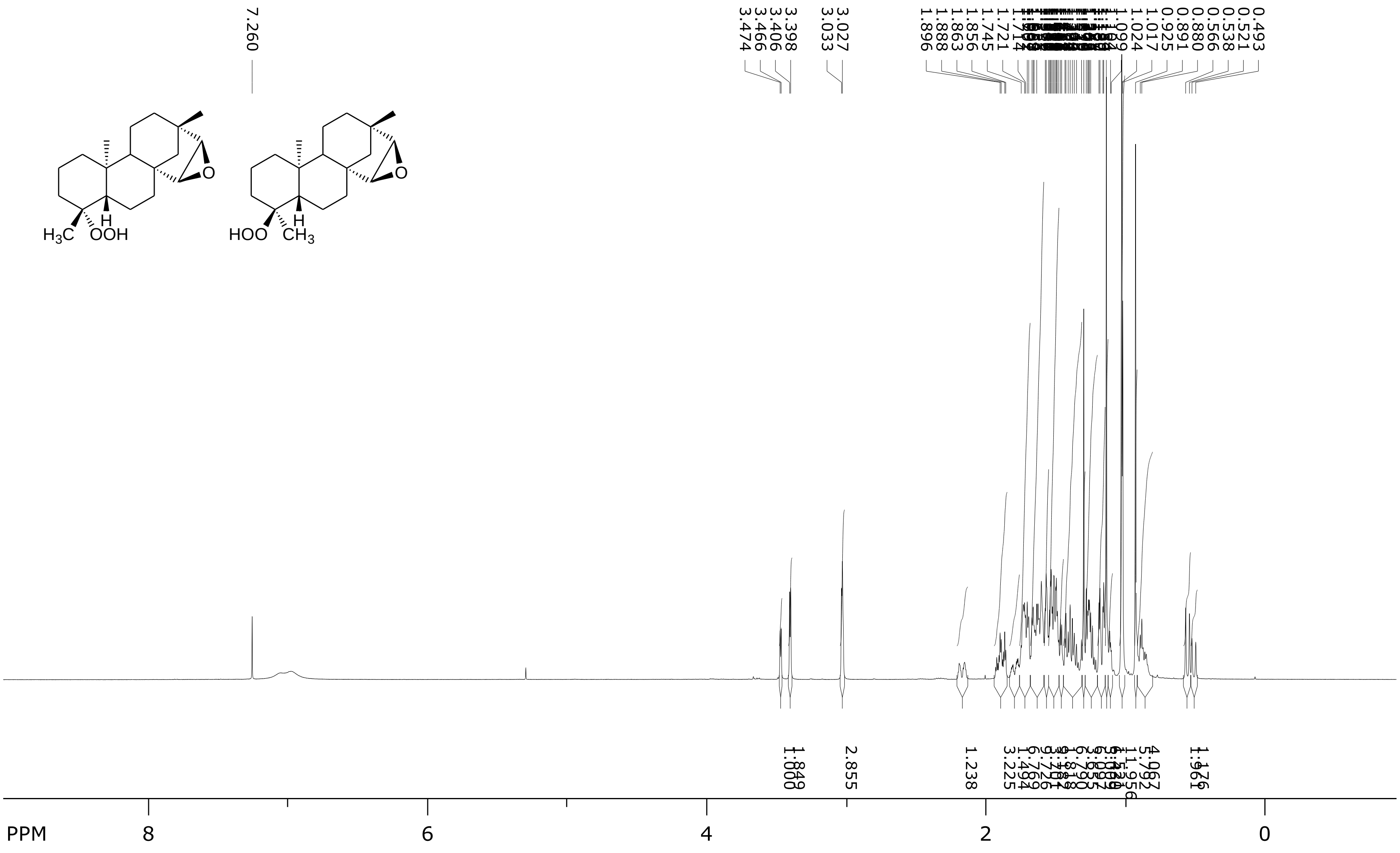

S23. ^{1}H NMR Spectrum (400 MHz) of **6a** and **6b** in $CDCl_3$ (Expansion δ 0.40 – 2.00)

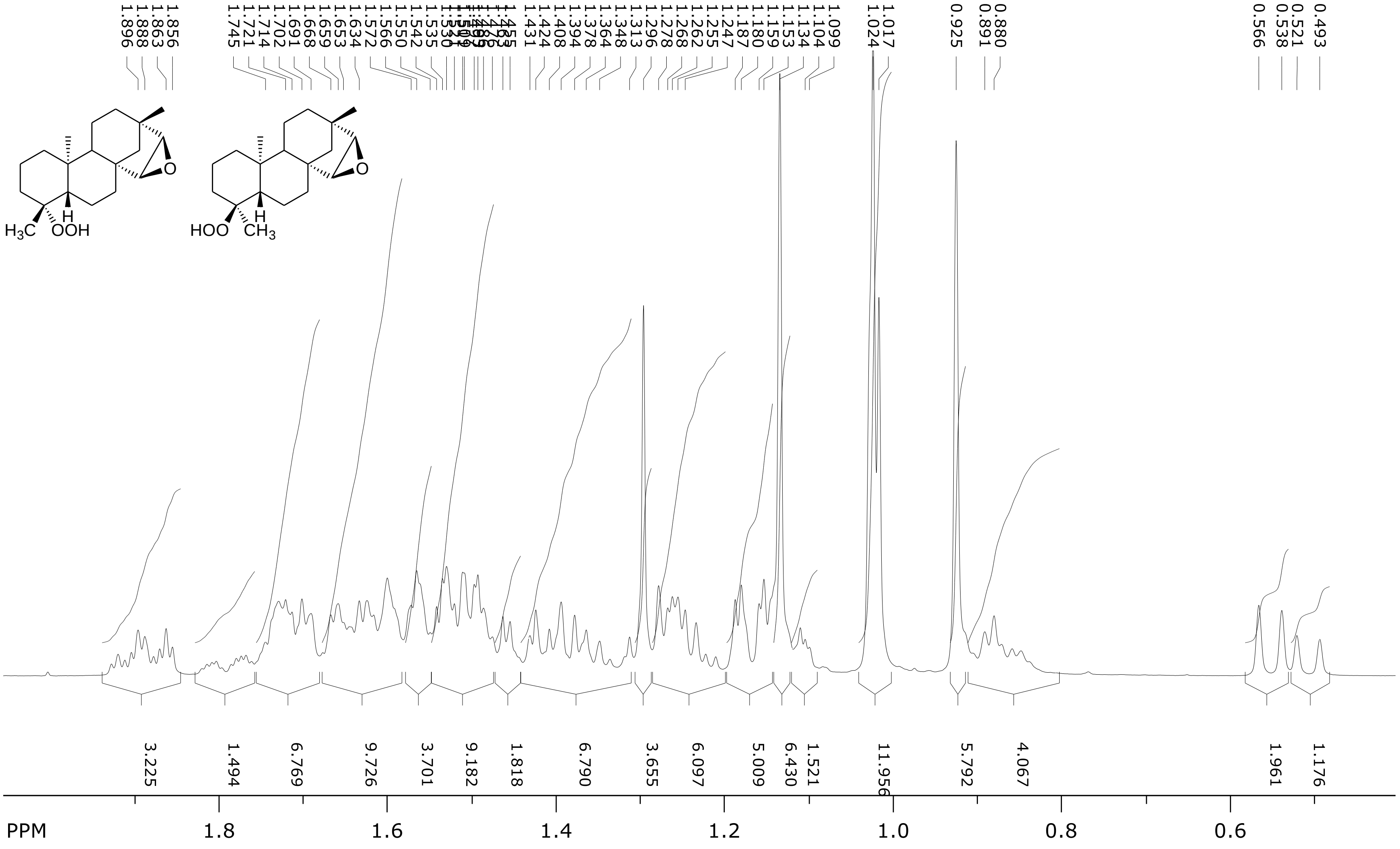

S24. ^{13}C NMR Spectrum (100 MHz) of **6a** and **6b** in $CDCl_3$

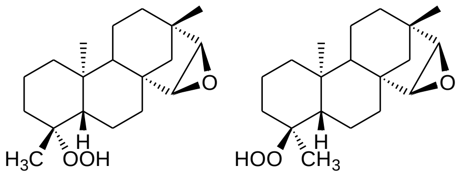

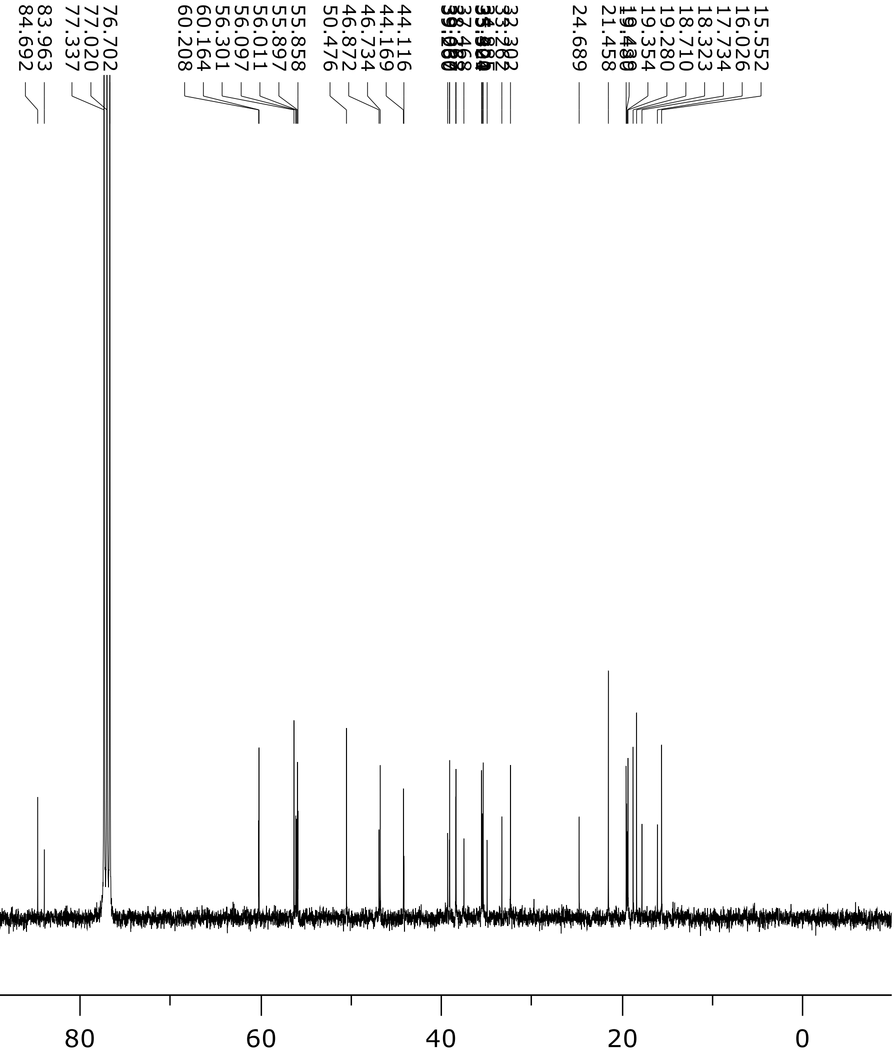

S25. ^{13}C NMR Spectrum (100 MHz) of **6a** and **6b** in $CDCl_3$ (Expansion δ 0.00 – 65.00)

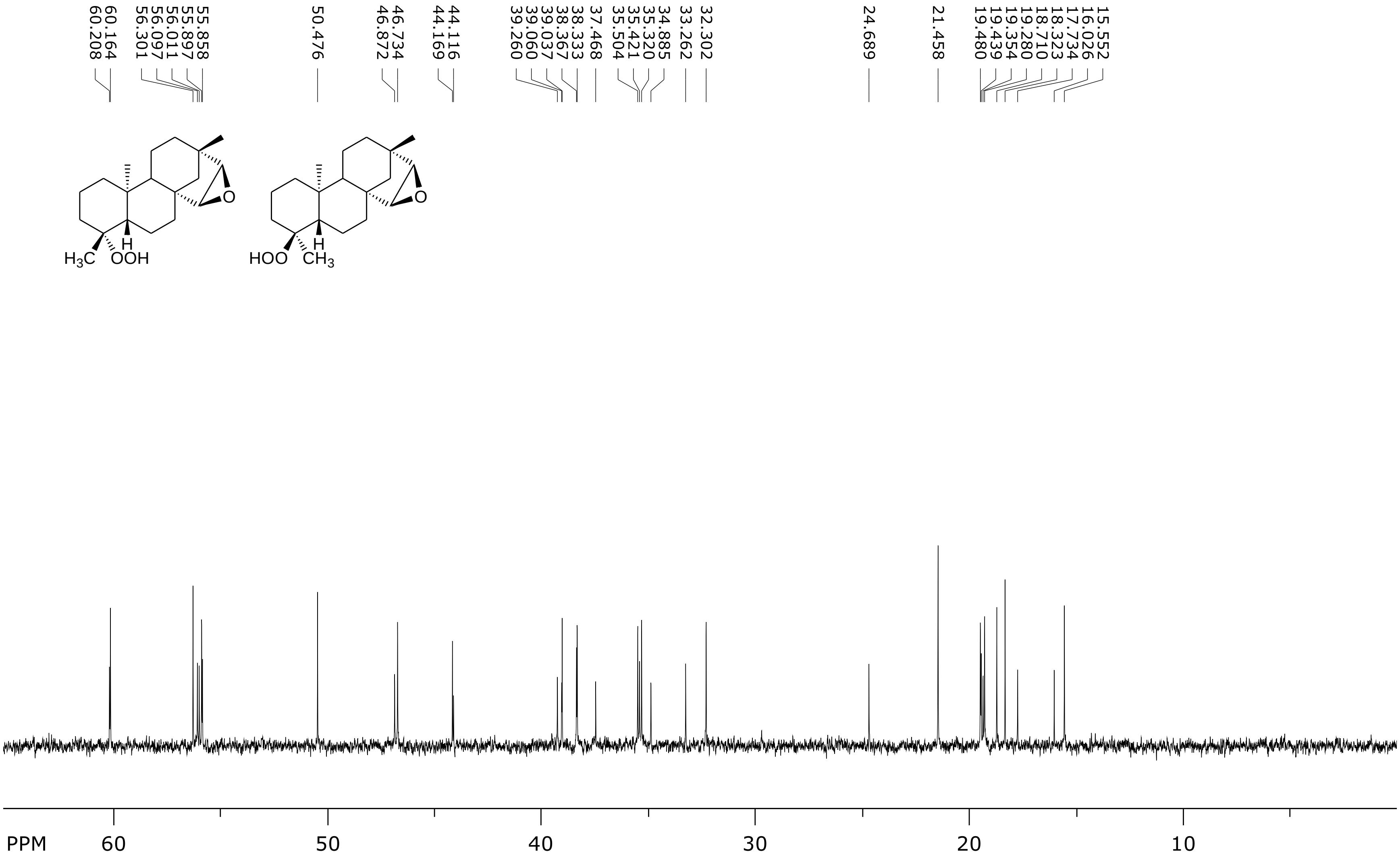

S26. DEPT 135 Spectrum (100 MHz) of **6a** and **6b** in $CDCl_3$

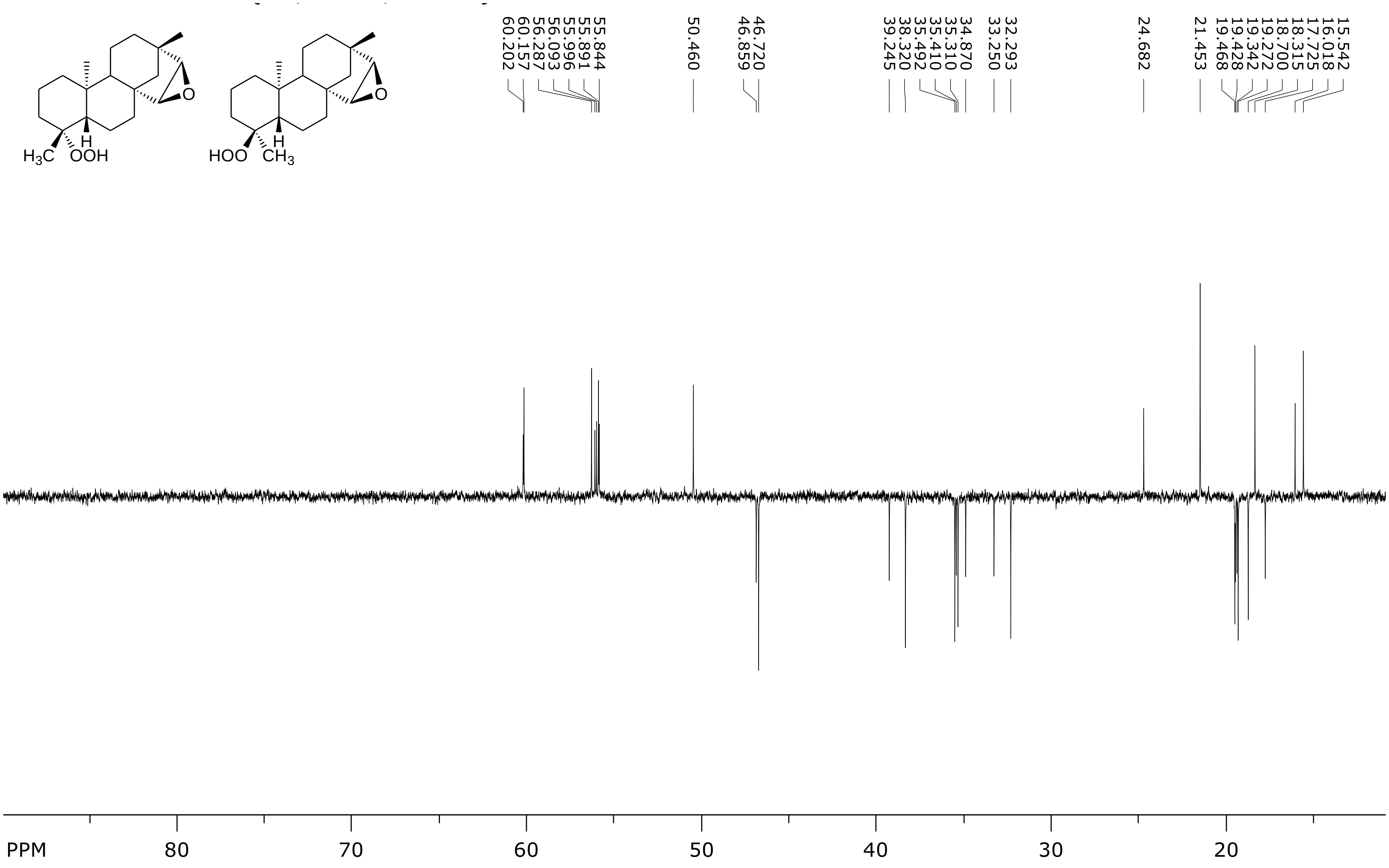

S27. HSQC Spectrum of **6a** and **6b** in $CDCl_3$

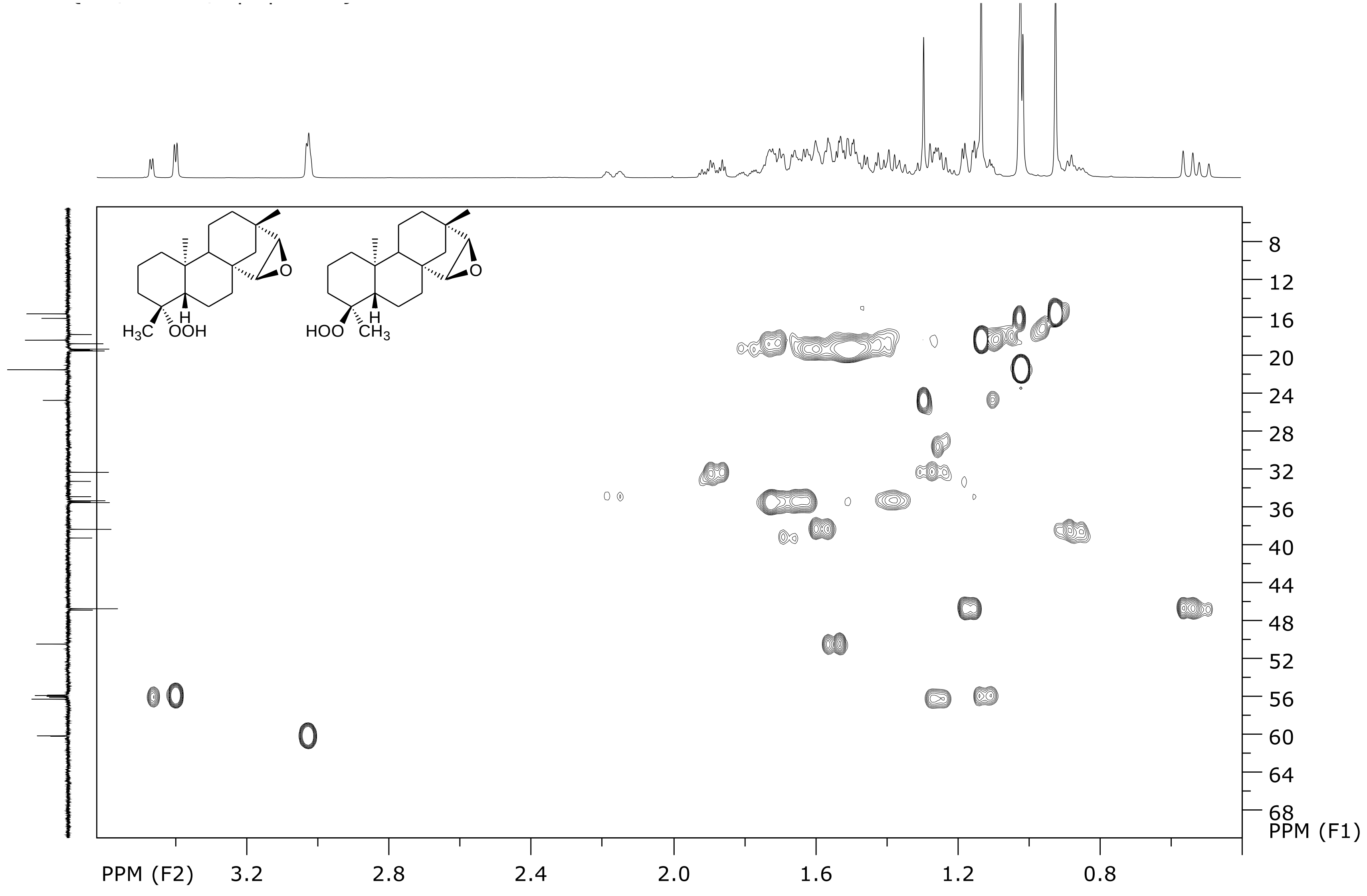

S28. HMBC Spectrum of **6a** and **6b** in $CDCl_3$

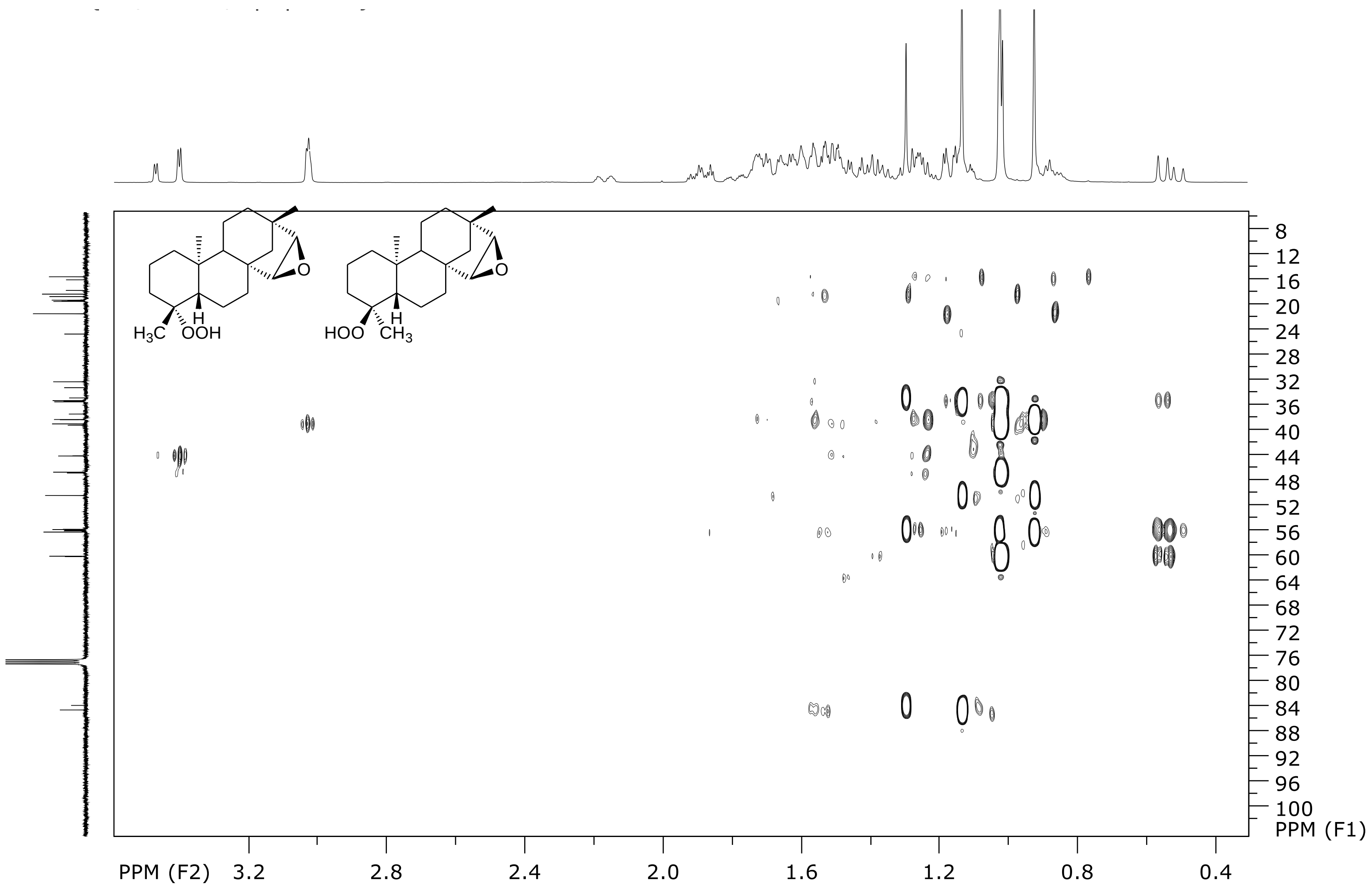

S29. 1D Selective Gradient NOESY spectrum (400 MHz) of **6a** and **6b** in $CDCl_3$; Irradiation at δ 0.924

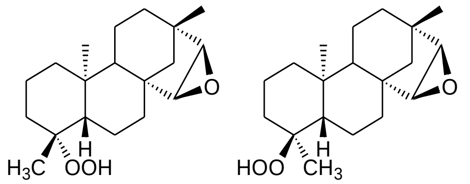

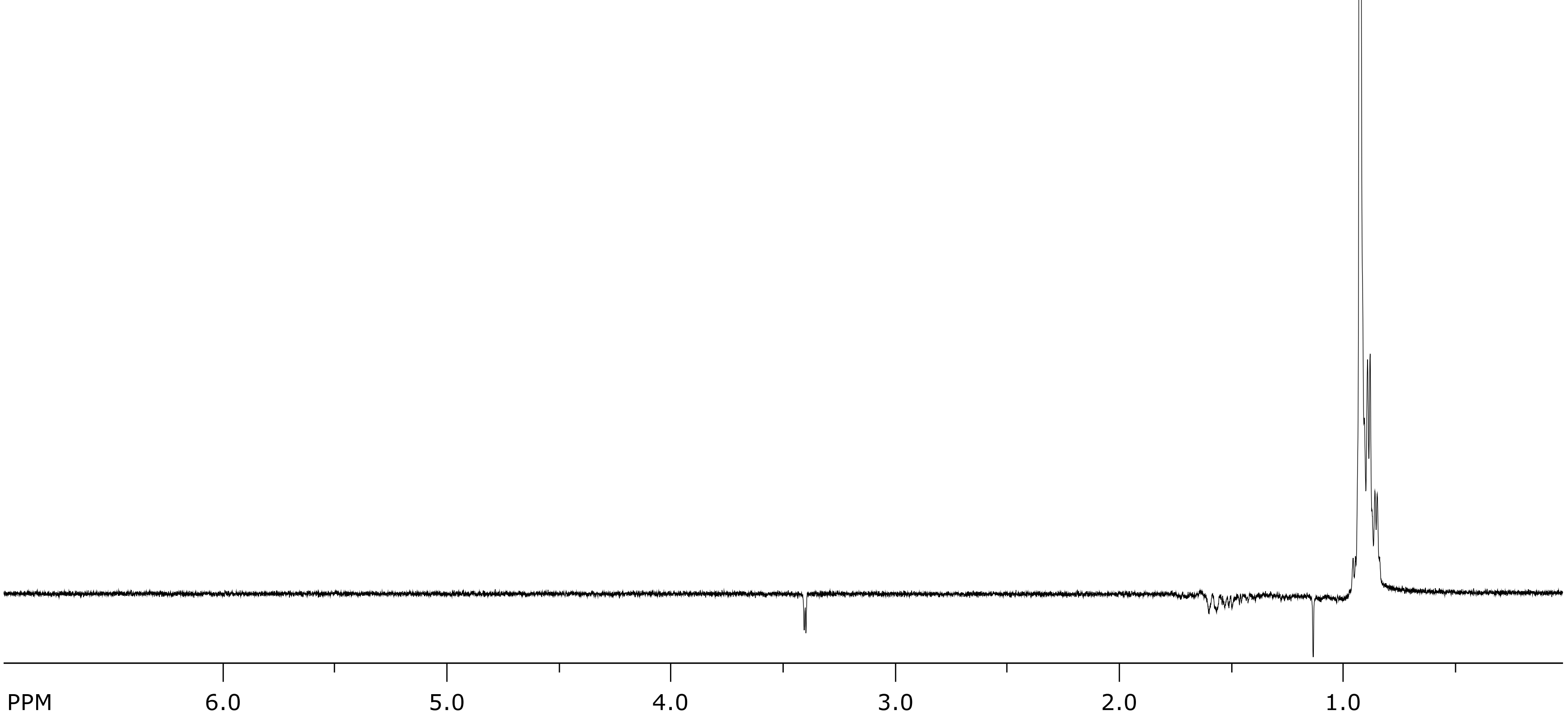

S30. 1D Selective Gradient NOESY spectrum (400 MHz) of **6a** and **6b** in $CDCl_3$; Irradiation at δ 1.134

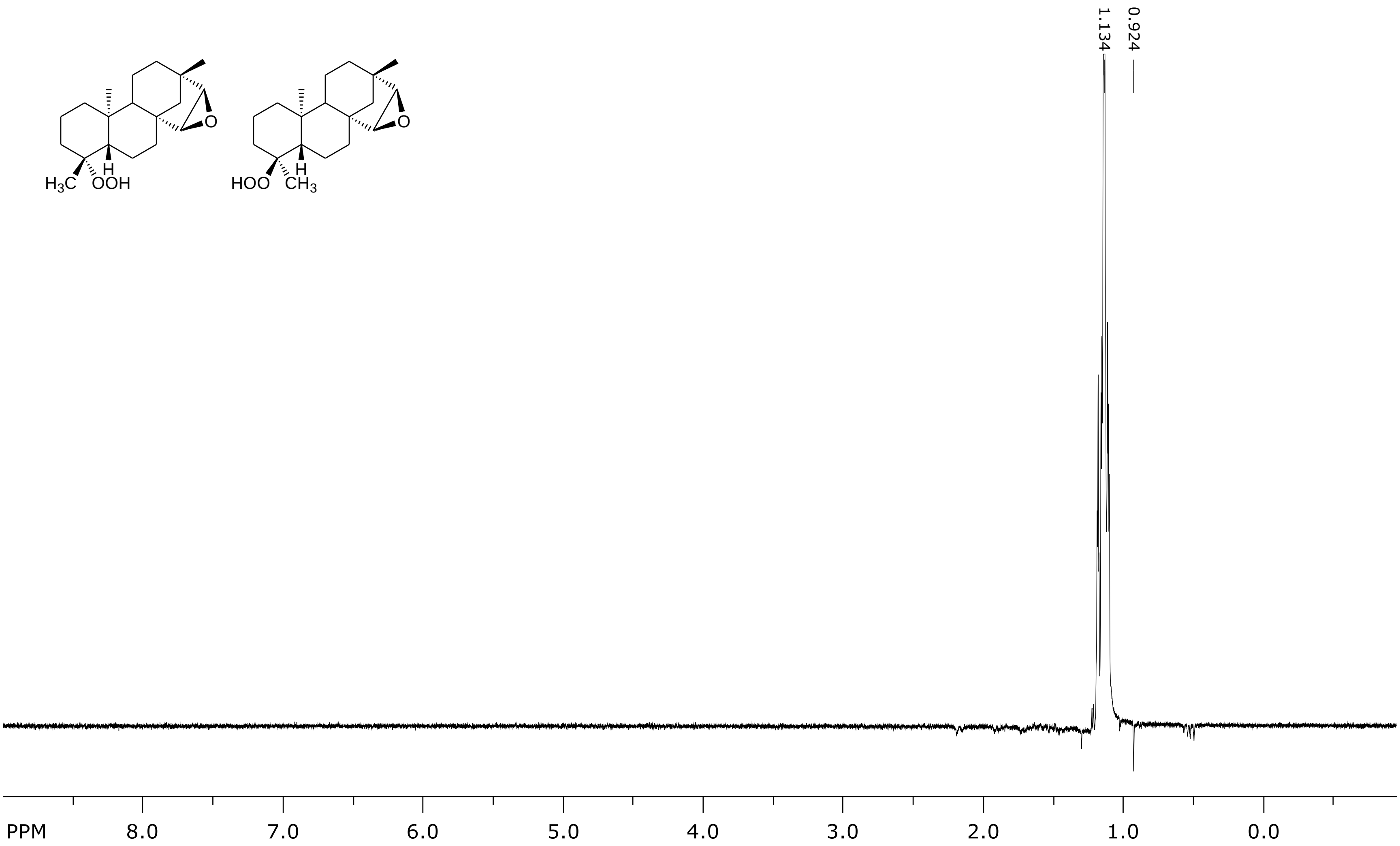

S31. 1D Selective Gradient NOESY spectrum (400 MHz) of **6a** and **6b** in $CDCl_3$; Irradiation at δ 1.296

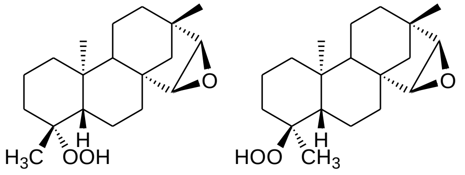

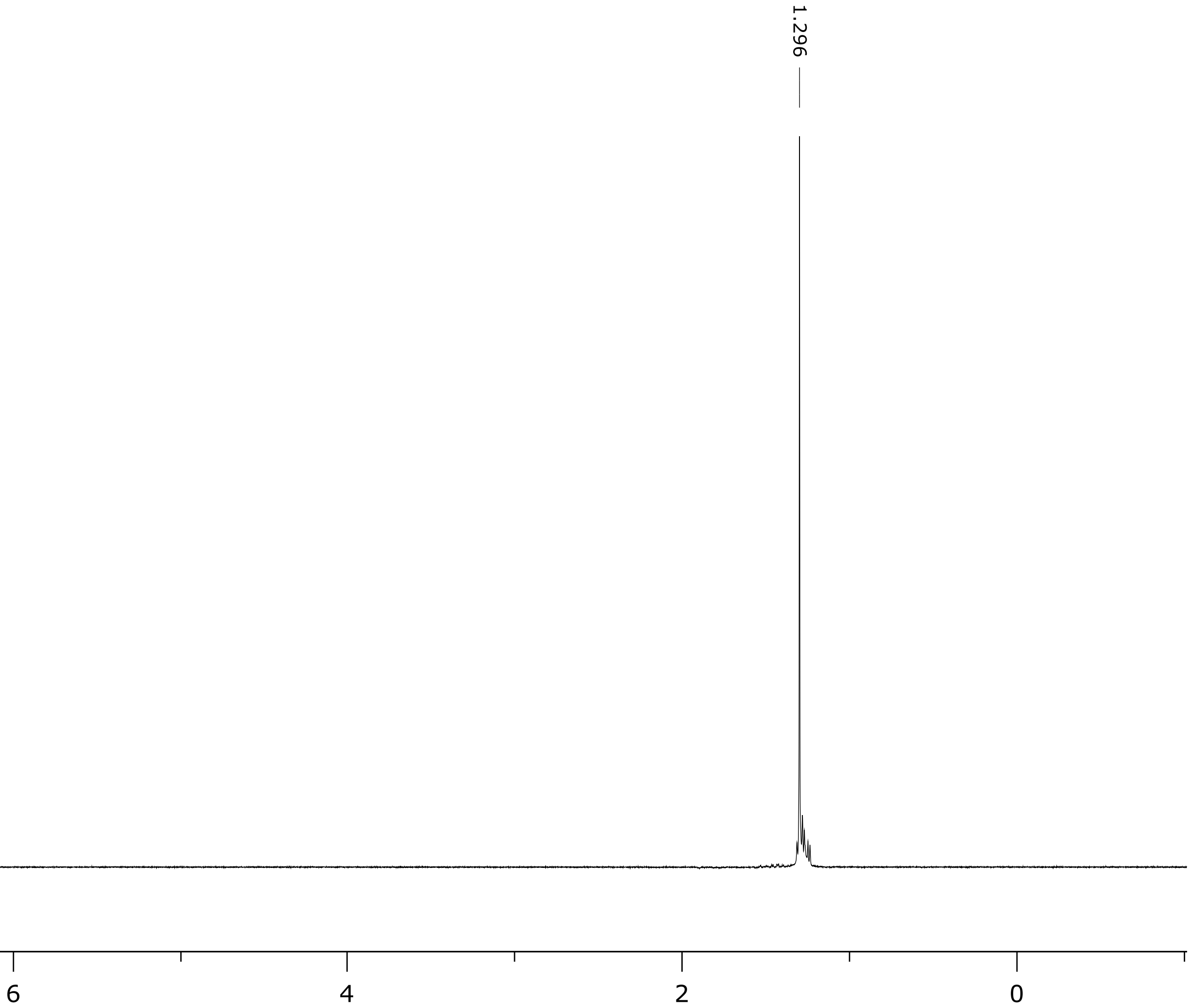

S32. ^{1}H NMR Spectrum (400 MHz) of **6a** in $CDCl_3$

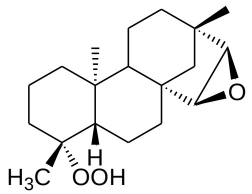

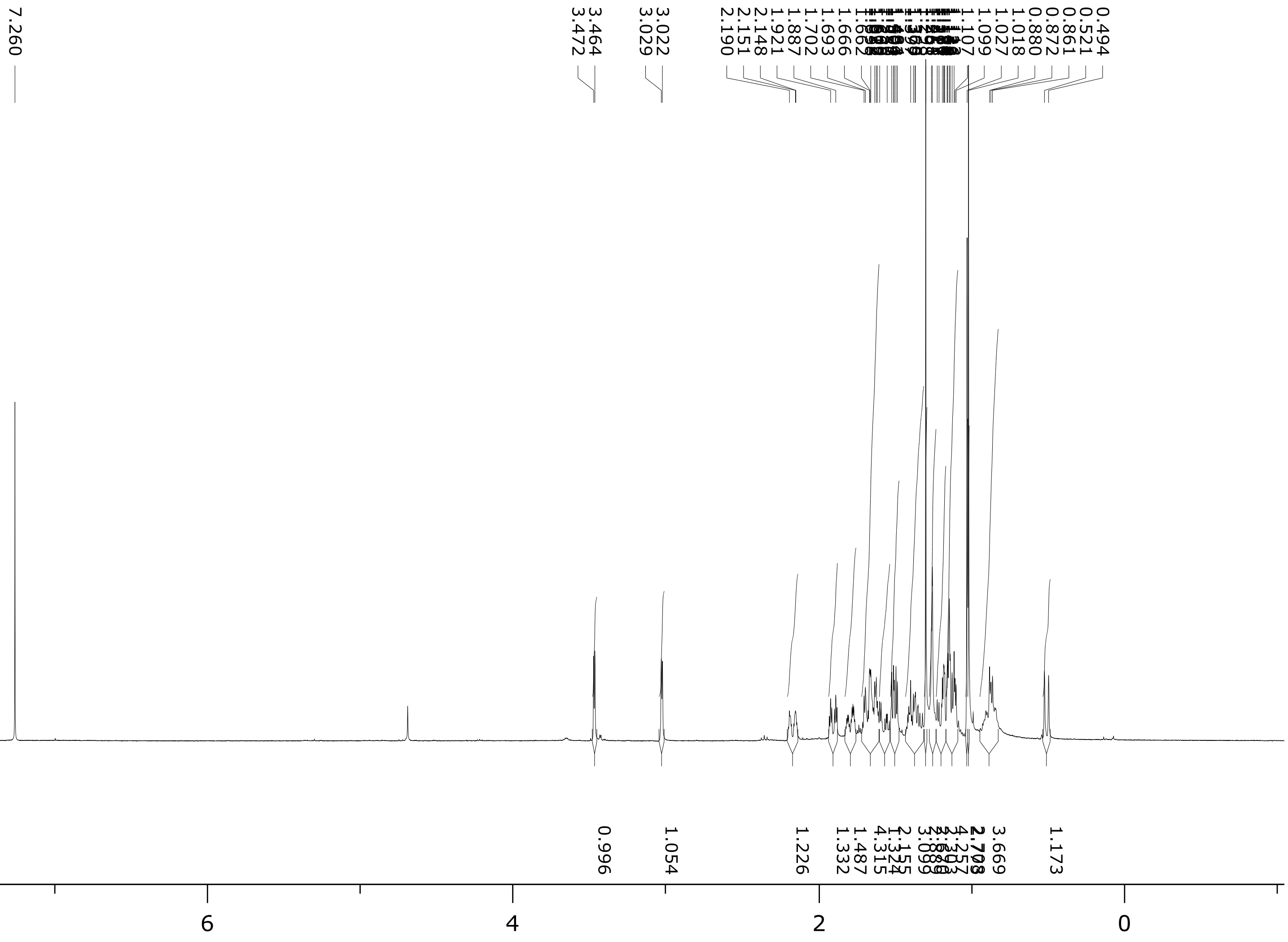

S33. ^{1}H NMR Spectrum (400 MHz) of **6a** in $CDCl_3$ (Expansion δ 0.40 – 3.50)

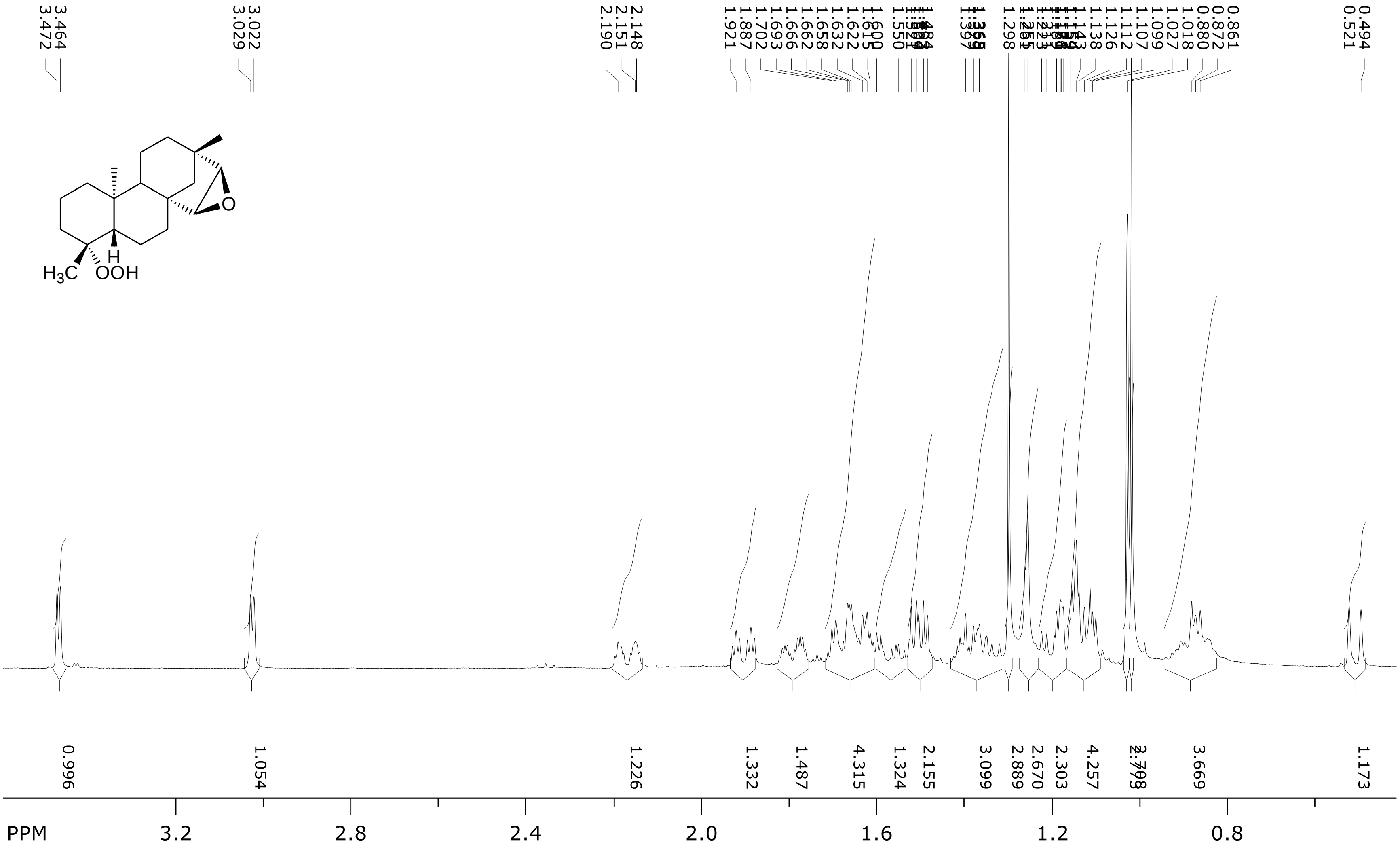

S34. ^{13}C NMR Spectrum (100 MHz) of **6a** in $CDCl_3$

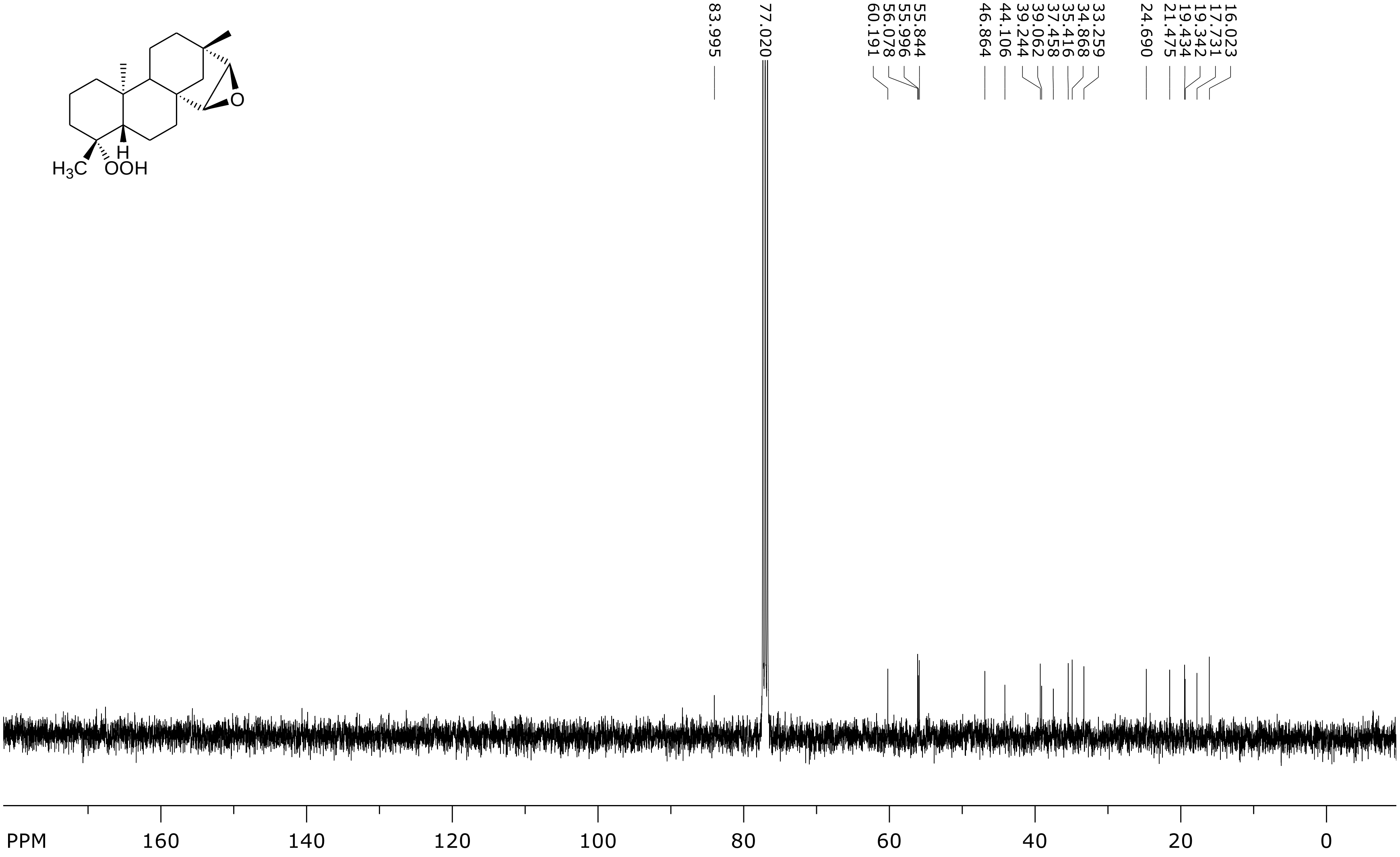

S35. ^{1}H NMR Spectrum (400 MHz) of **6b** in $CDCl_3$

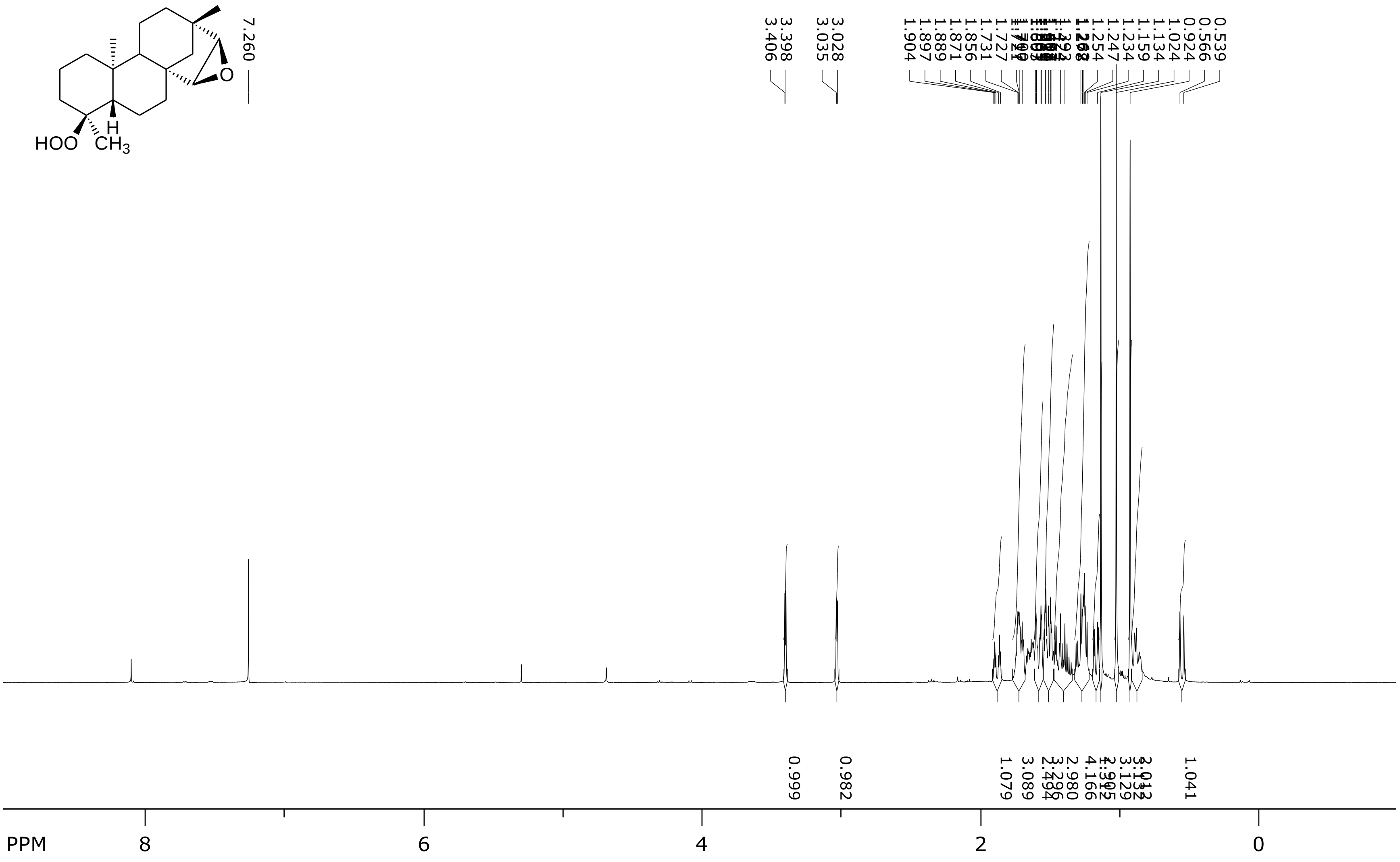

S36. ^{1}H NMR Spectrum (400 MHz) of **6b** in $CDCl_3$ (Expansion δ 0.40 – 3.50)

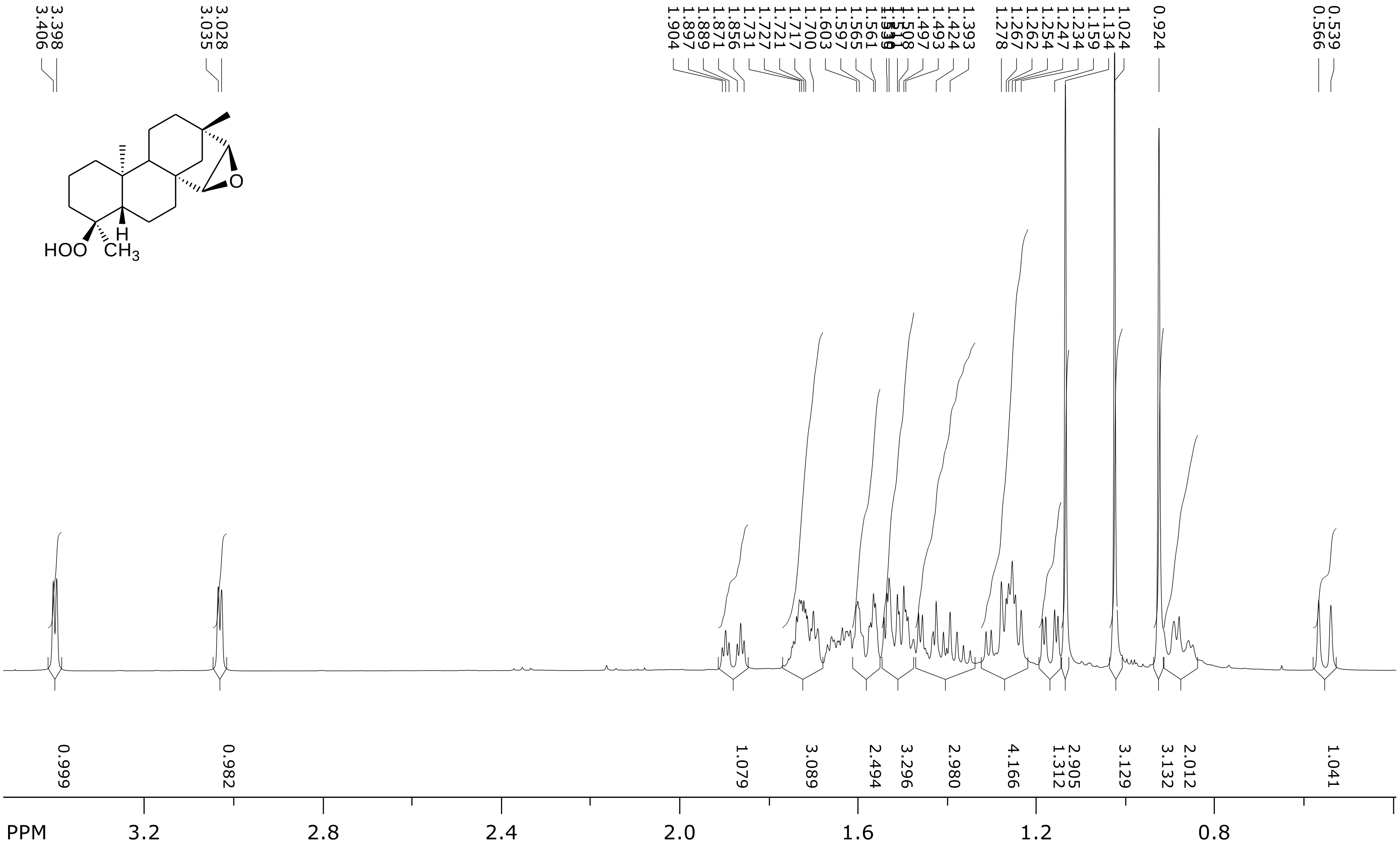

S37. ^{13}C NMR Spectrum (100 MHz) of **6b** in $CDCl_3$

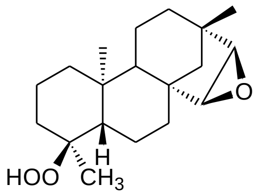

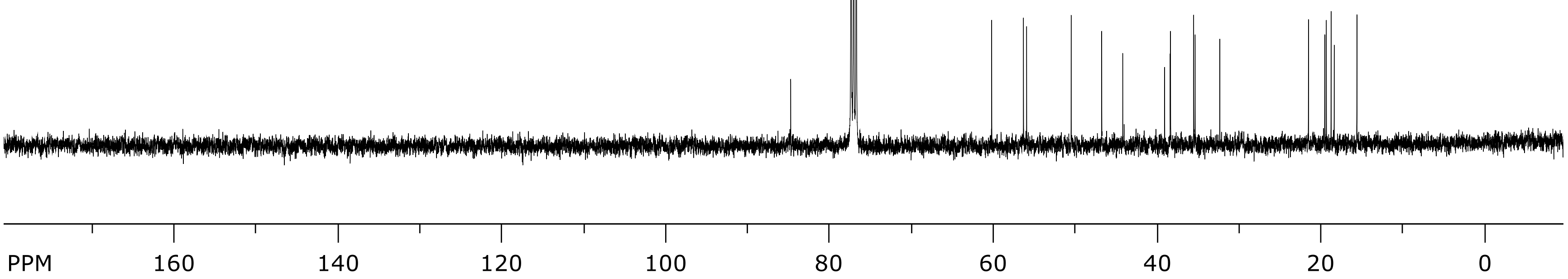

S38. ^{1}H NMR Spectrum (400 MHz) of **7** in $CDCl_3$

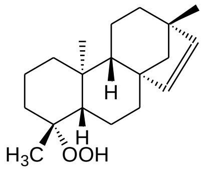

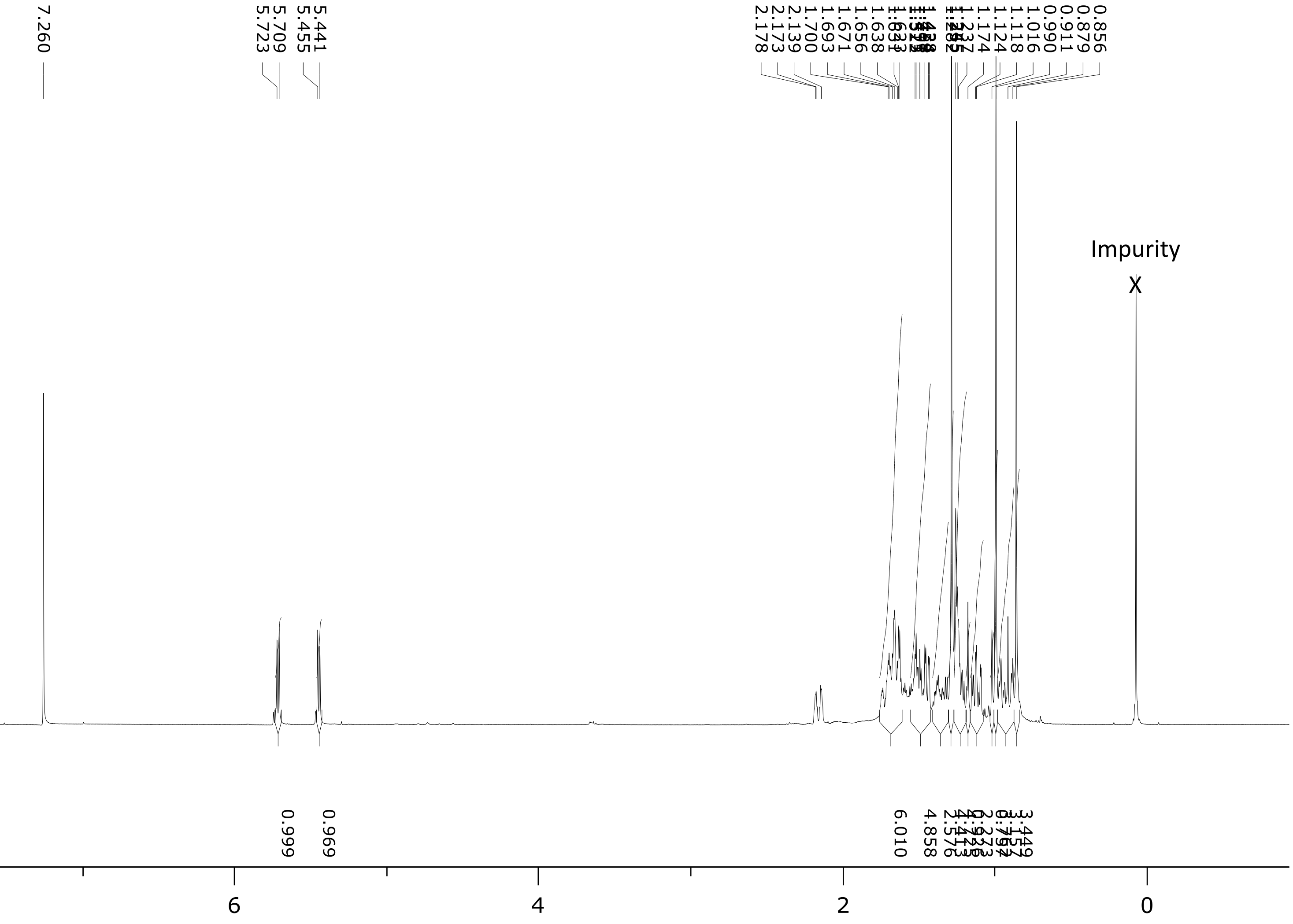

S39. ^{13}C NMR Spectrum (100 MHz) of **7** in $CDCl_3$

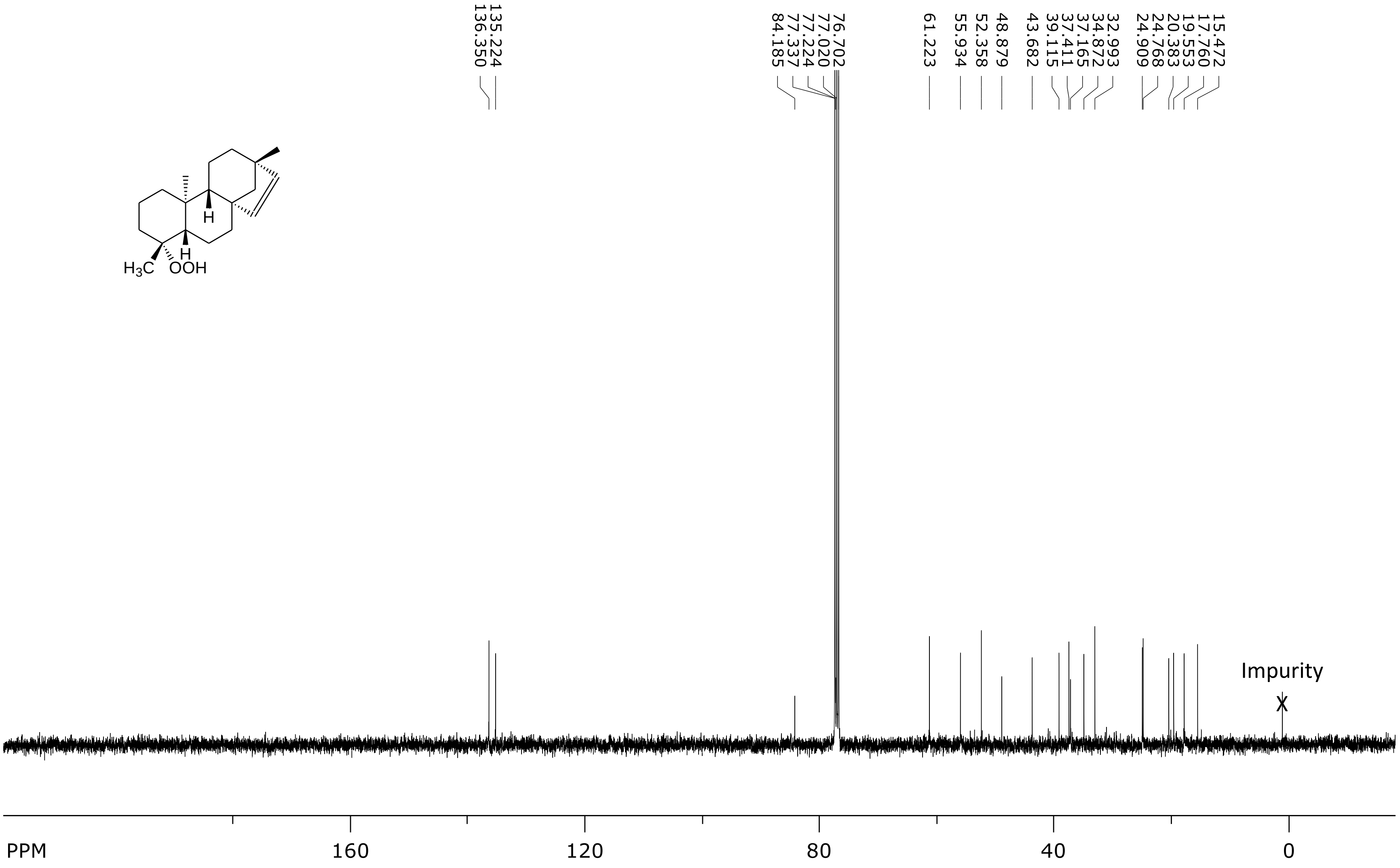

S40. DEPT 135 Spectrum (100 MHz) of **7** in $CDCl_3$

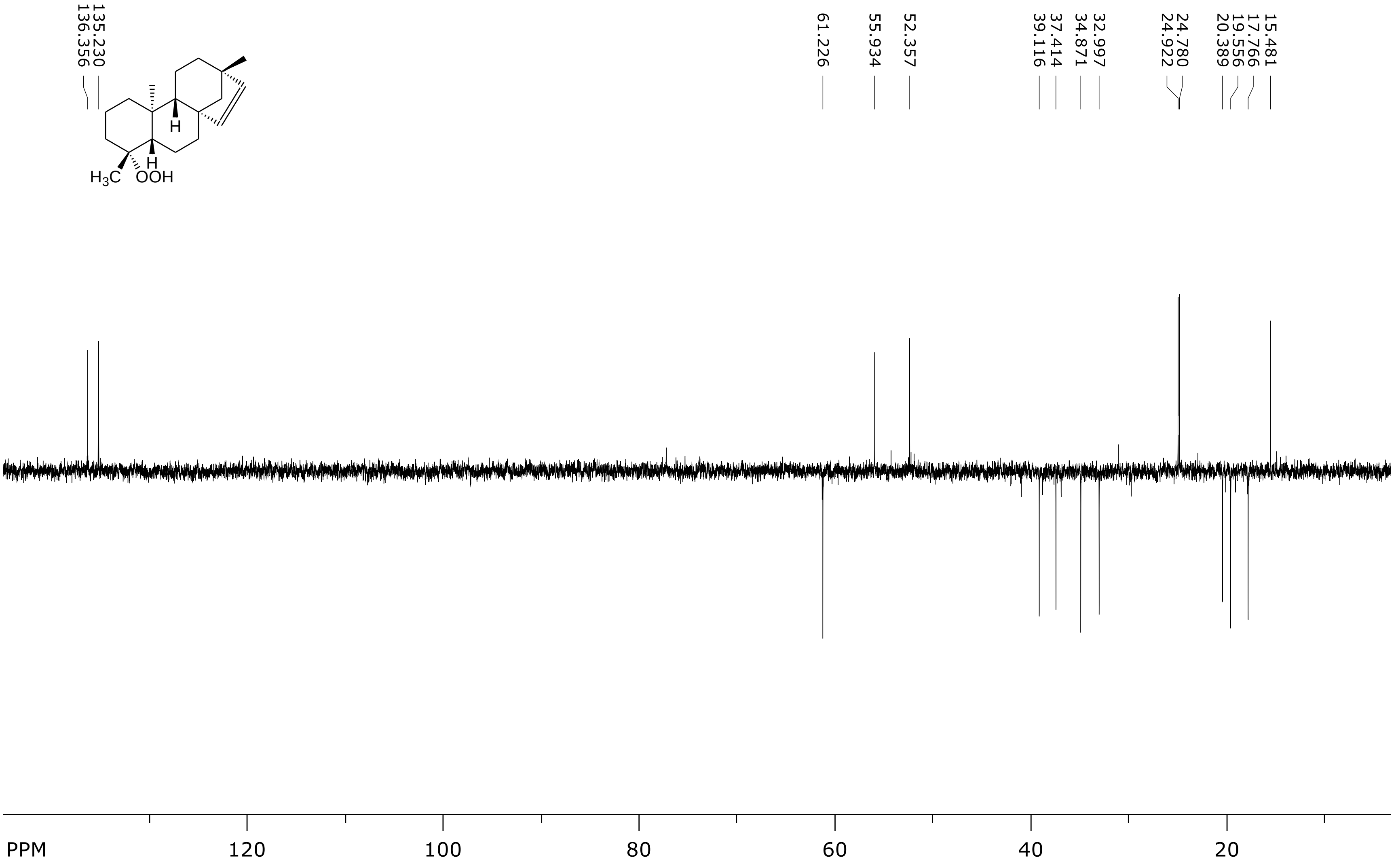

S41. HSQC Spectrum of **7** in $CDCl_3$

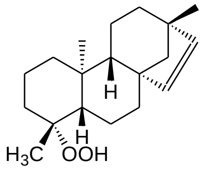

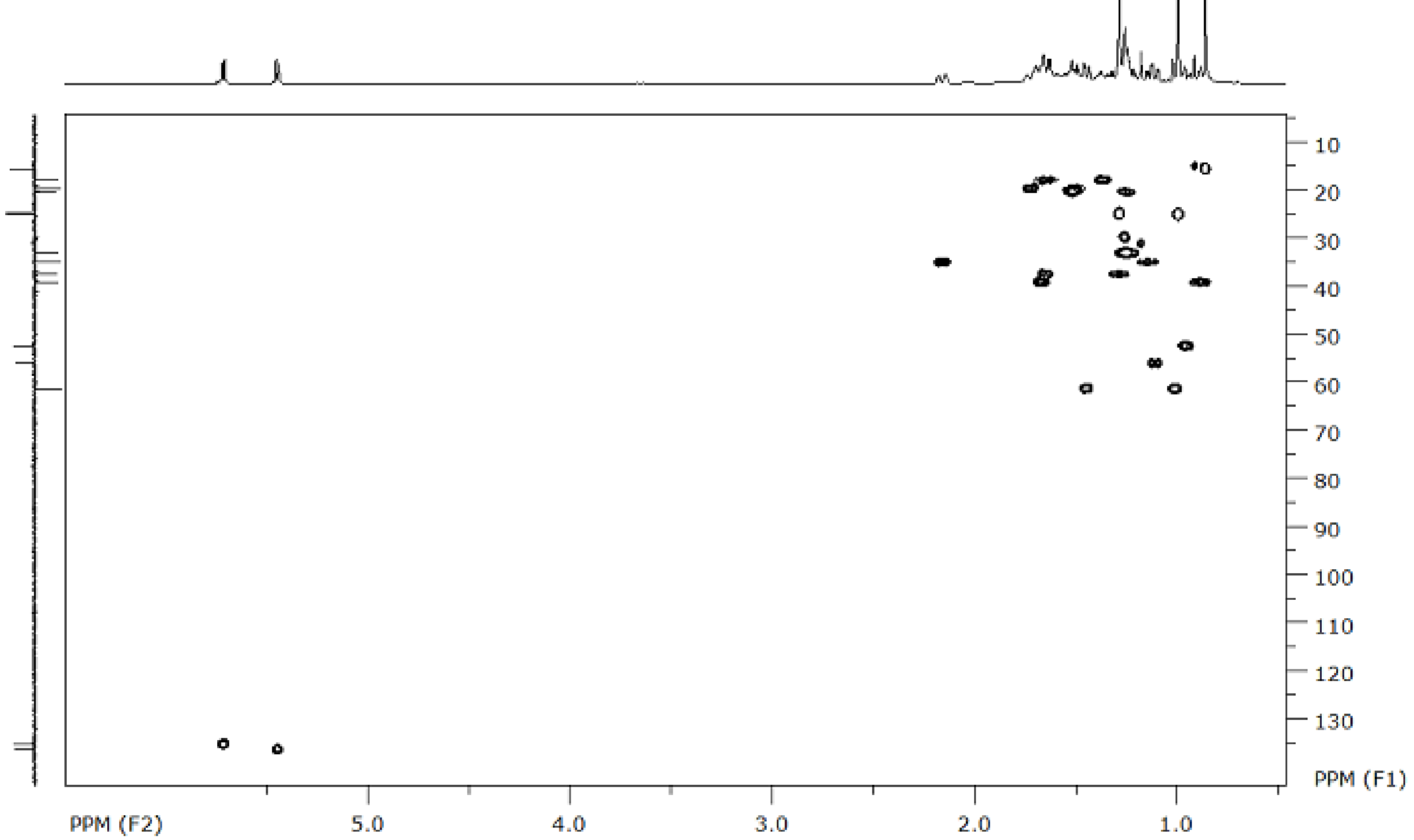

S42. HMBC Spectrum of **7** in $CDCl_3$

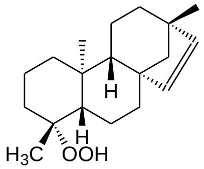

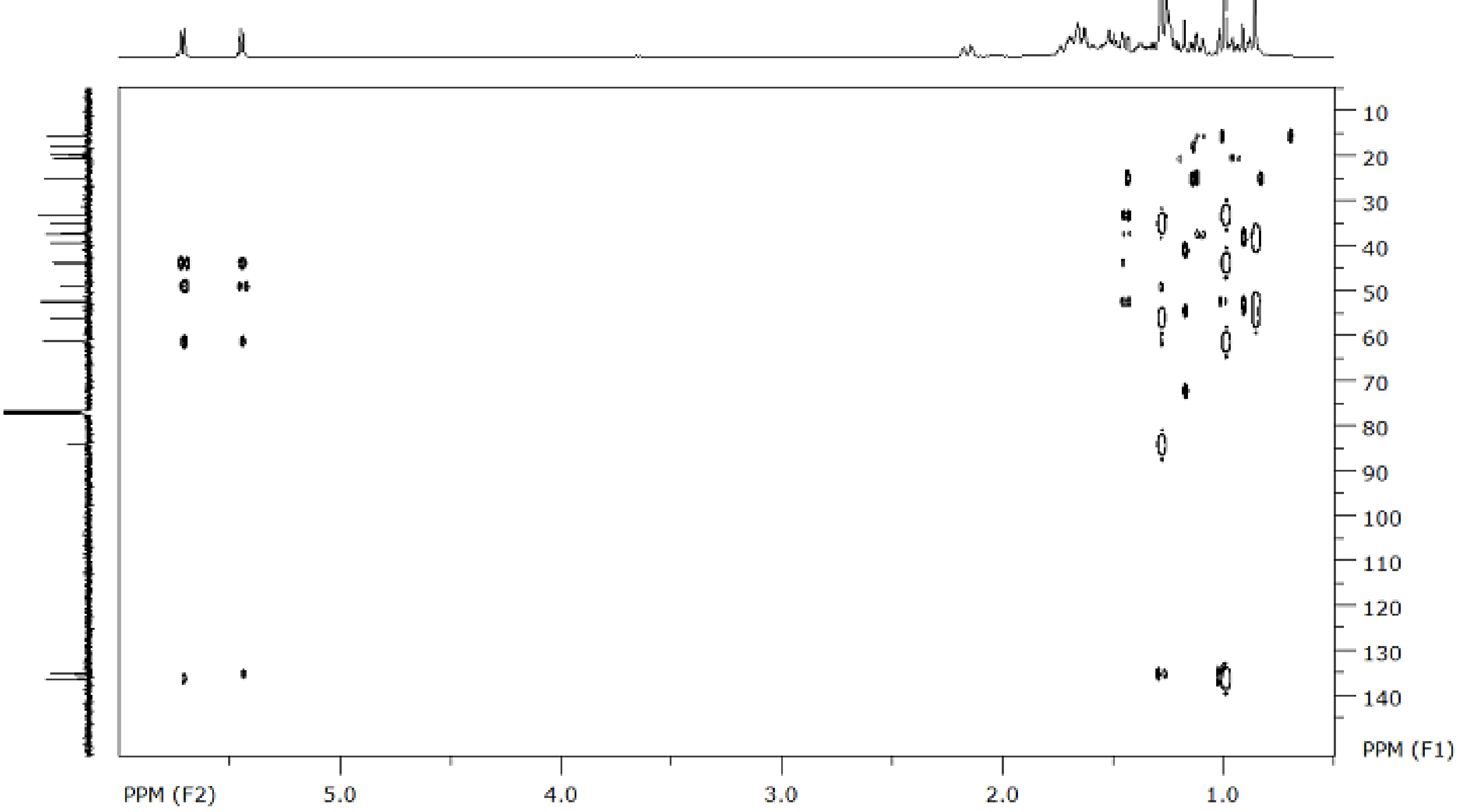

S43. 1D Selective Gradient NOESY spectrum (400 MHz) of **7** in $CDCl_3$; Irradiation at δ 0.856

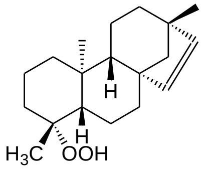

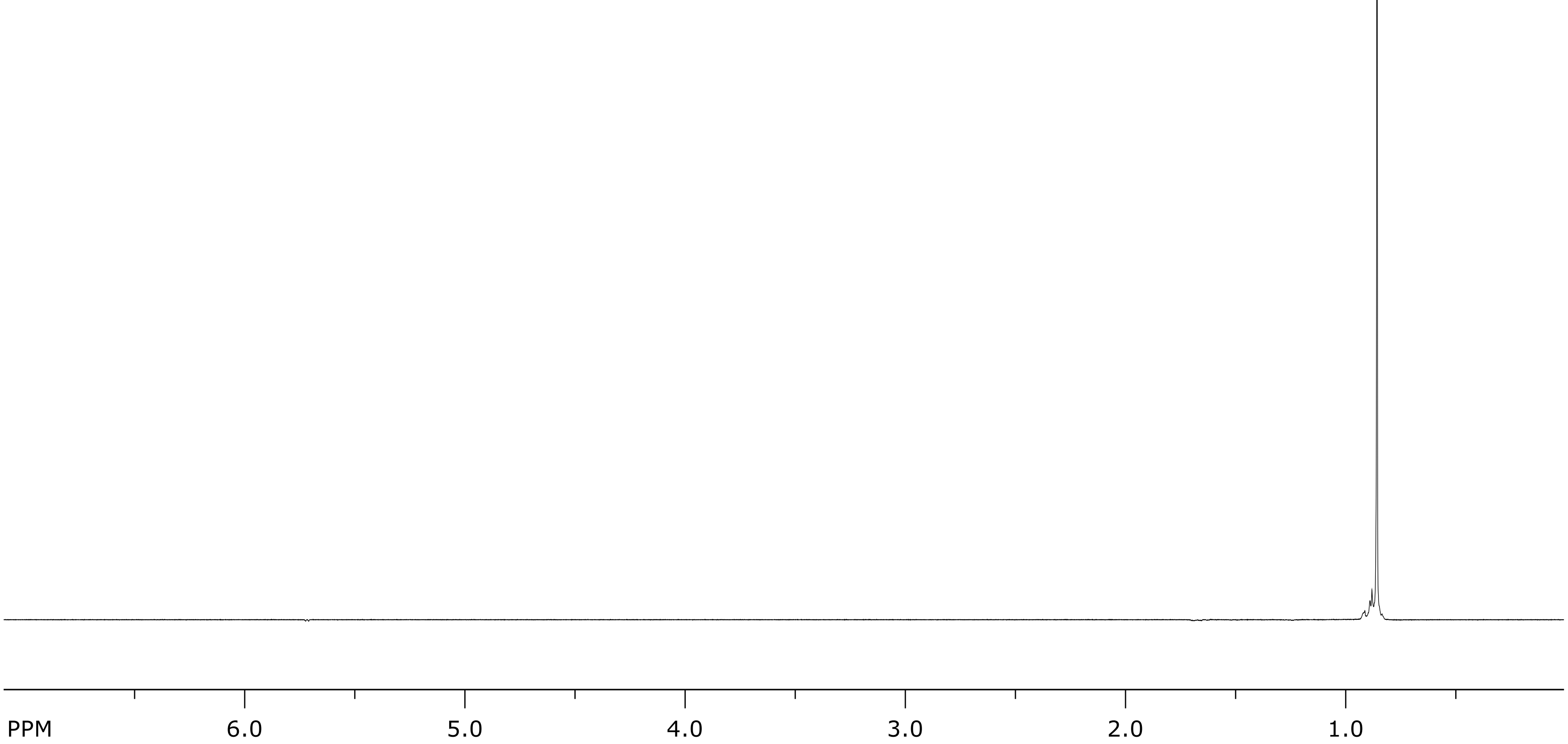

S44. 1D Selective Gradient NOESY spectrum (400 MHz) of **7** in $CDCl_3$; Irradiation at δ 1.282

1D Selective Gradient NOESY Freq: 1.282

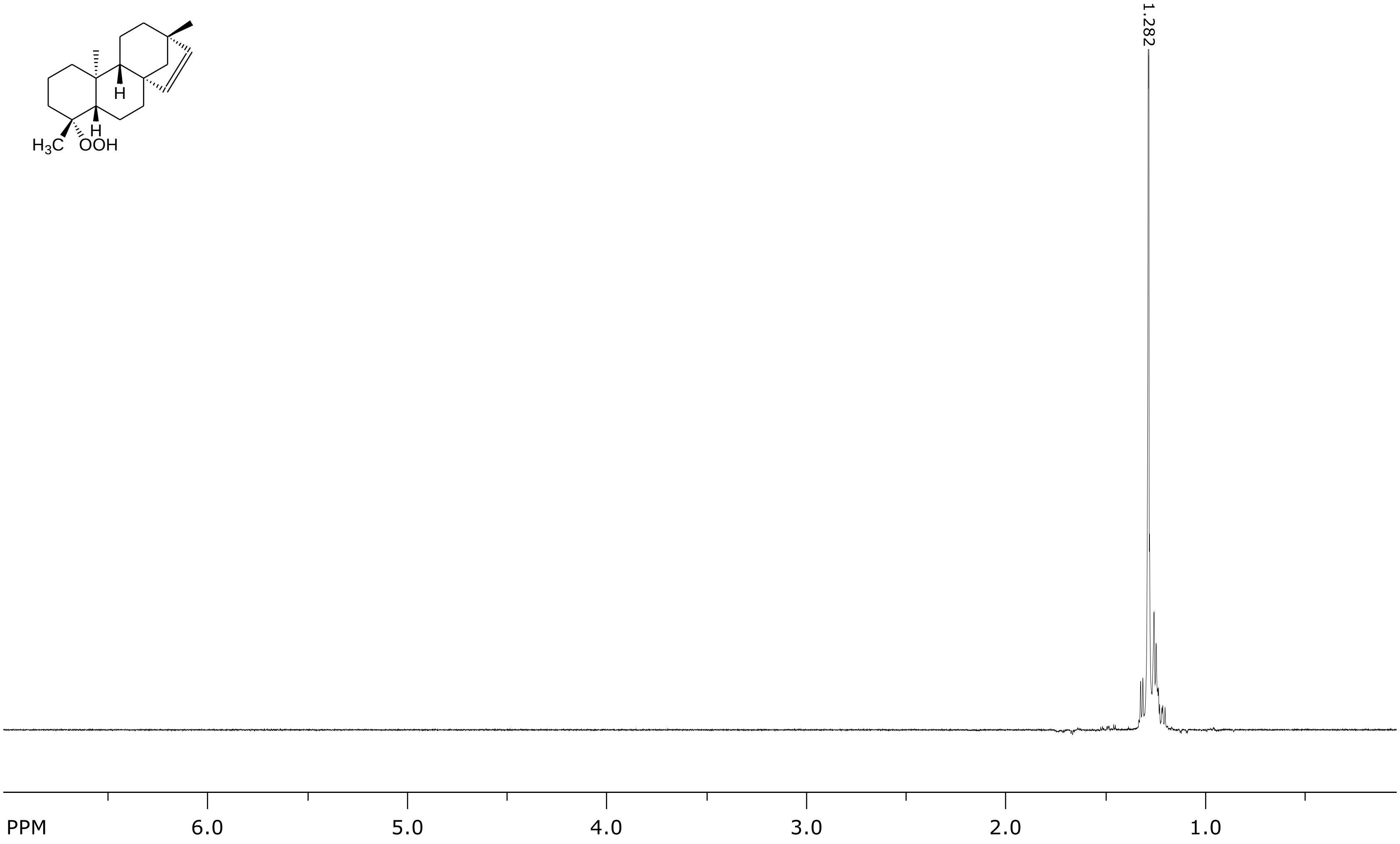

S45. ^{1}H NMR Spectrum (400 MHz) of **8** in $CDCl_3$

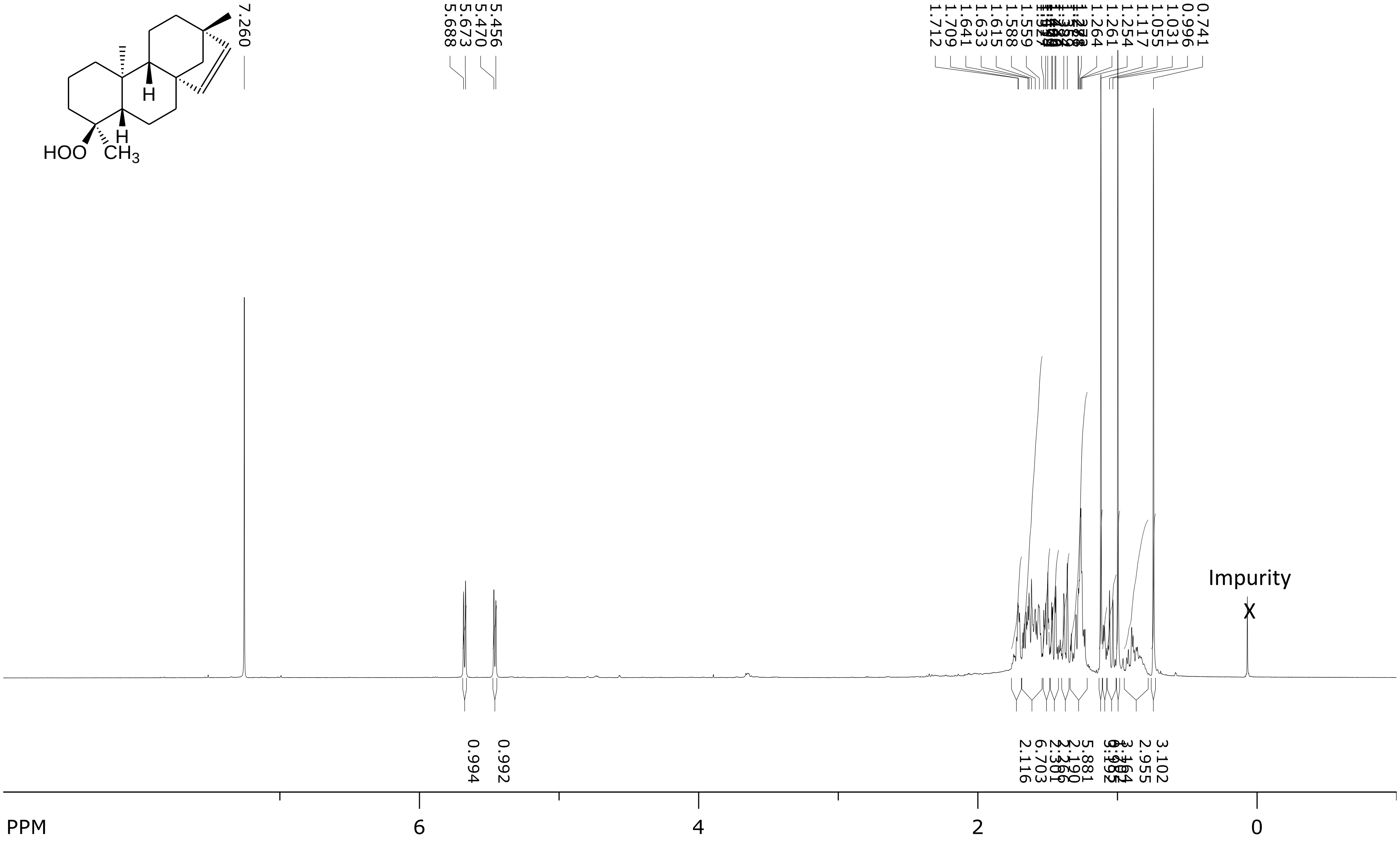

S46. ^{13}C NMR Spectrum (100 MHz) of **8** in $CDCl_3$

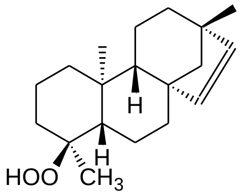

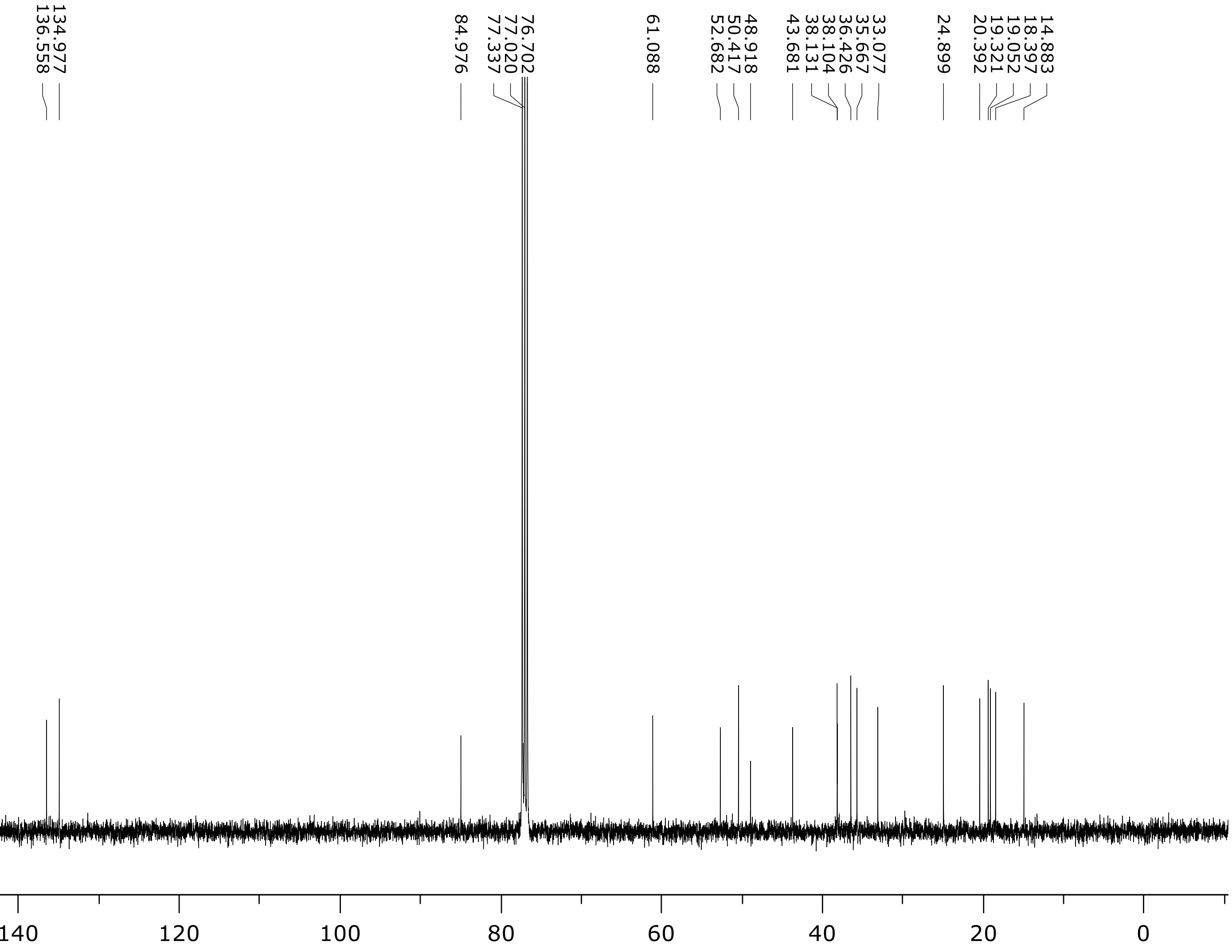

S47. DEPT135 Spectrum (100 MHz) of **8** in $CDCl_3$

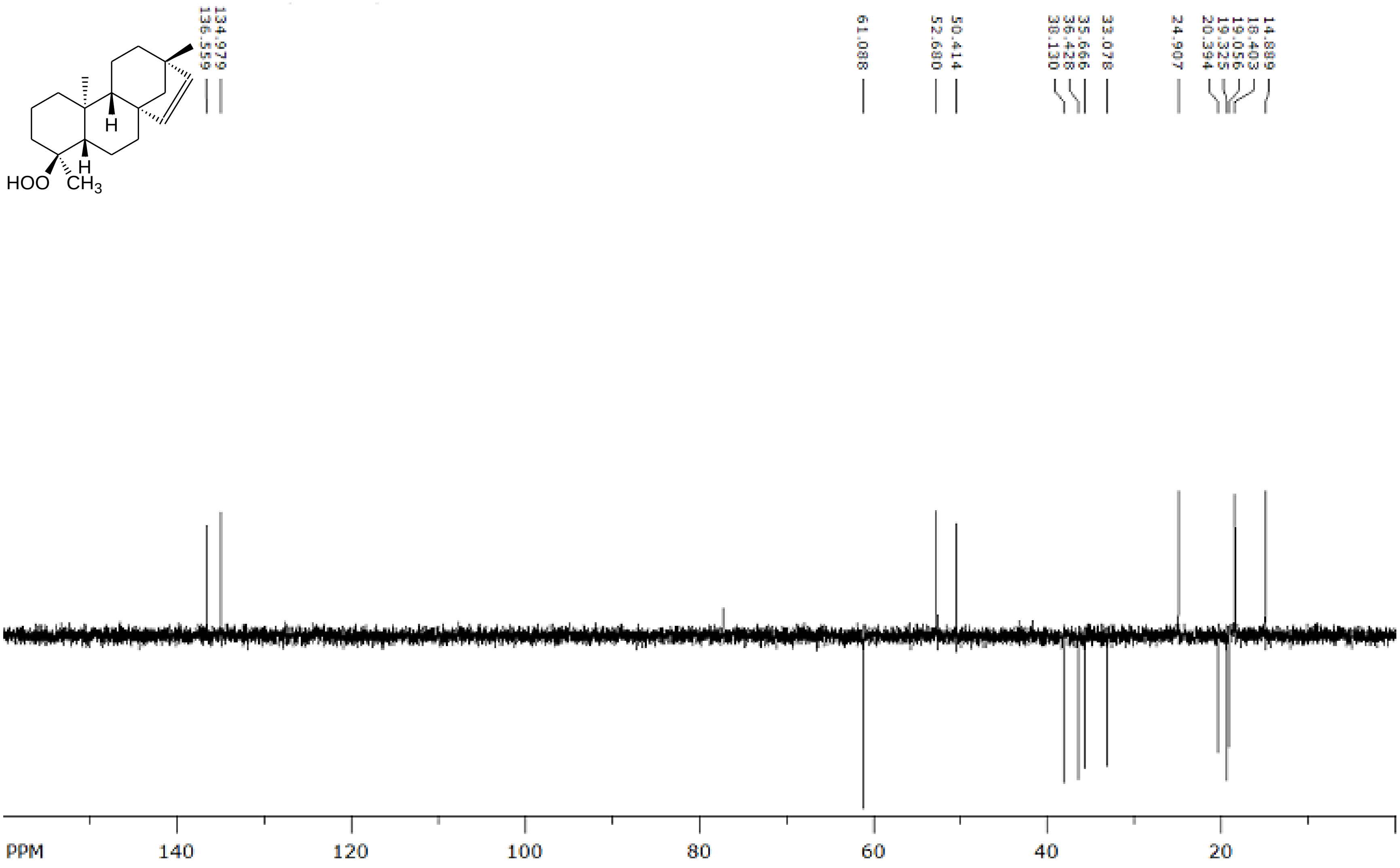

S48. HSQC Spectrum of **8** in $CDCl_3$

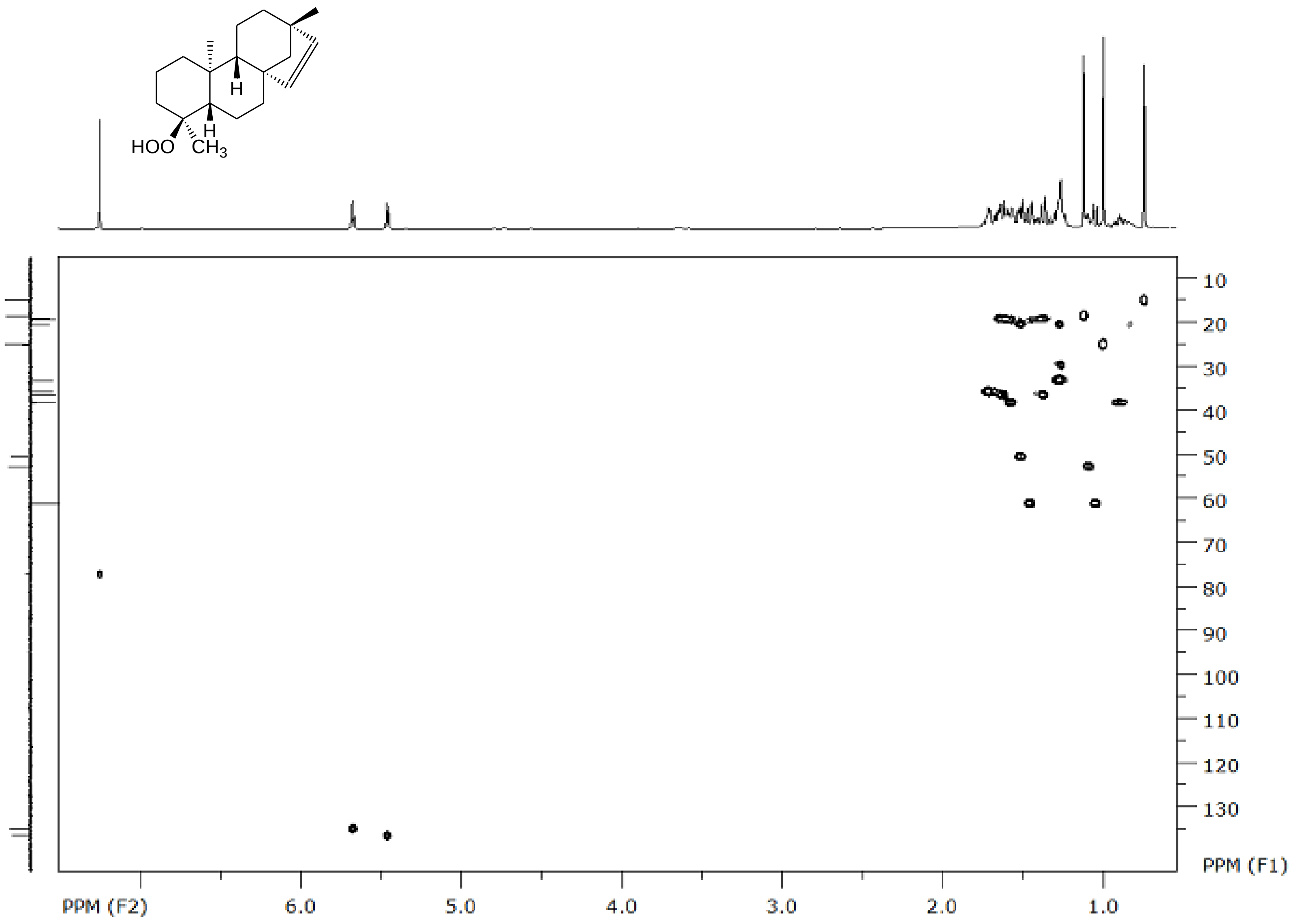

S49. HMBC Spectrum of **8** in $CDCl_3$

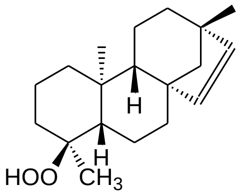

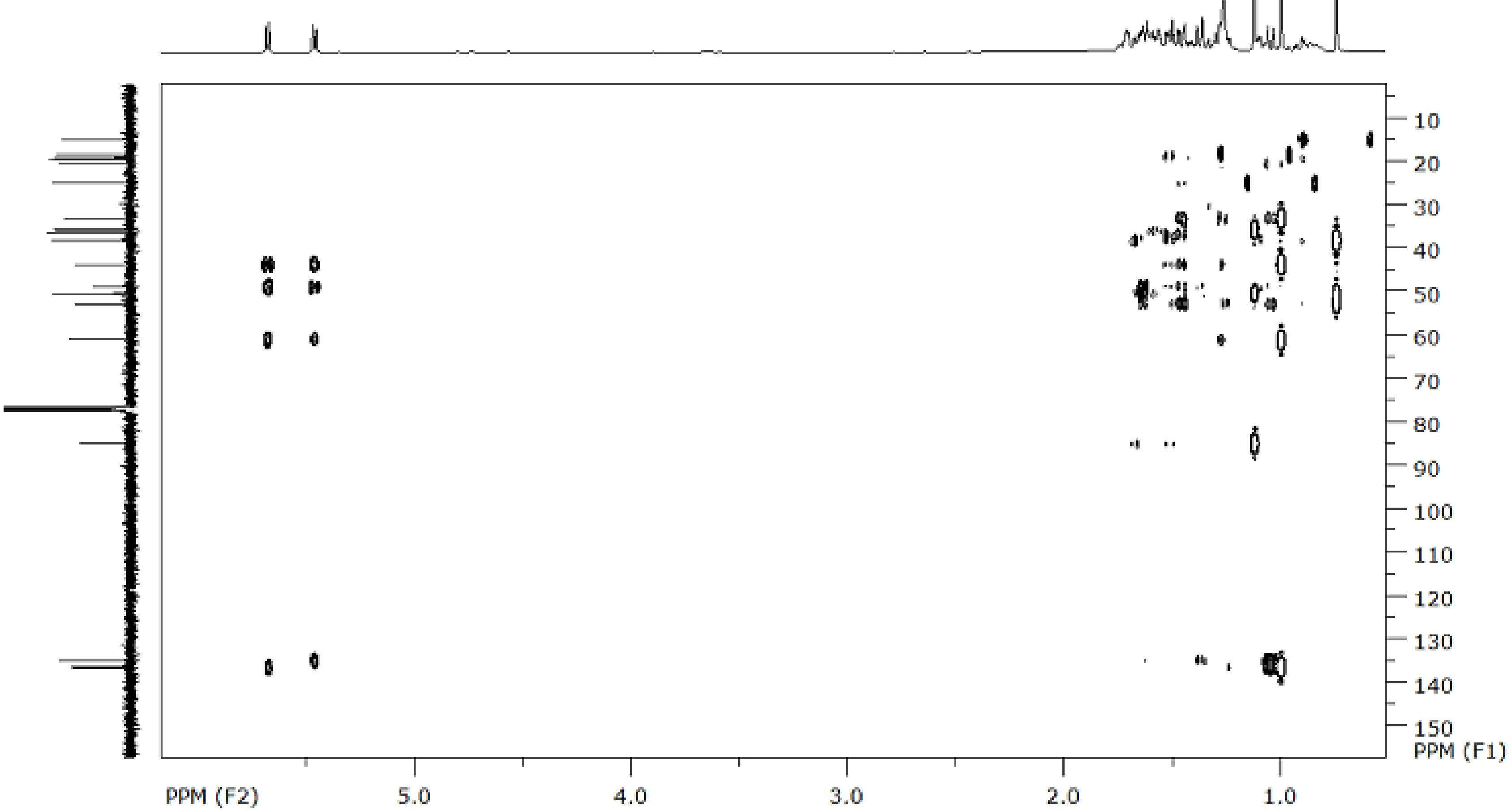

S50. 1D Selective Gradient NOESY spectrum (400 MHz) of **8** in $CDCl_3$; irradiation at δ 0.740

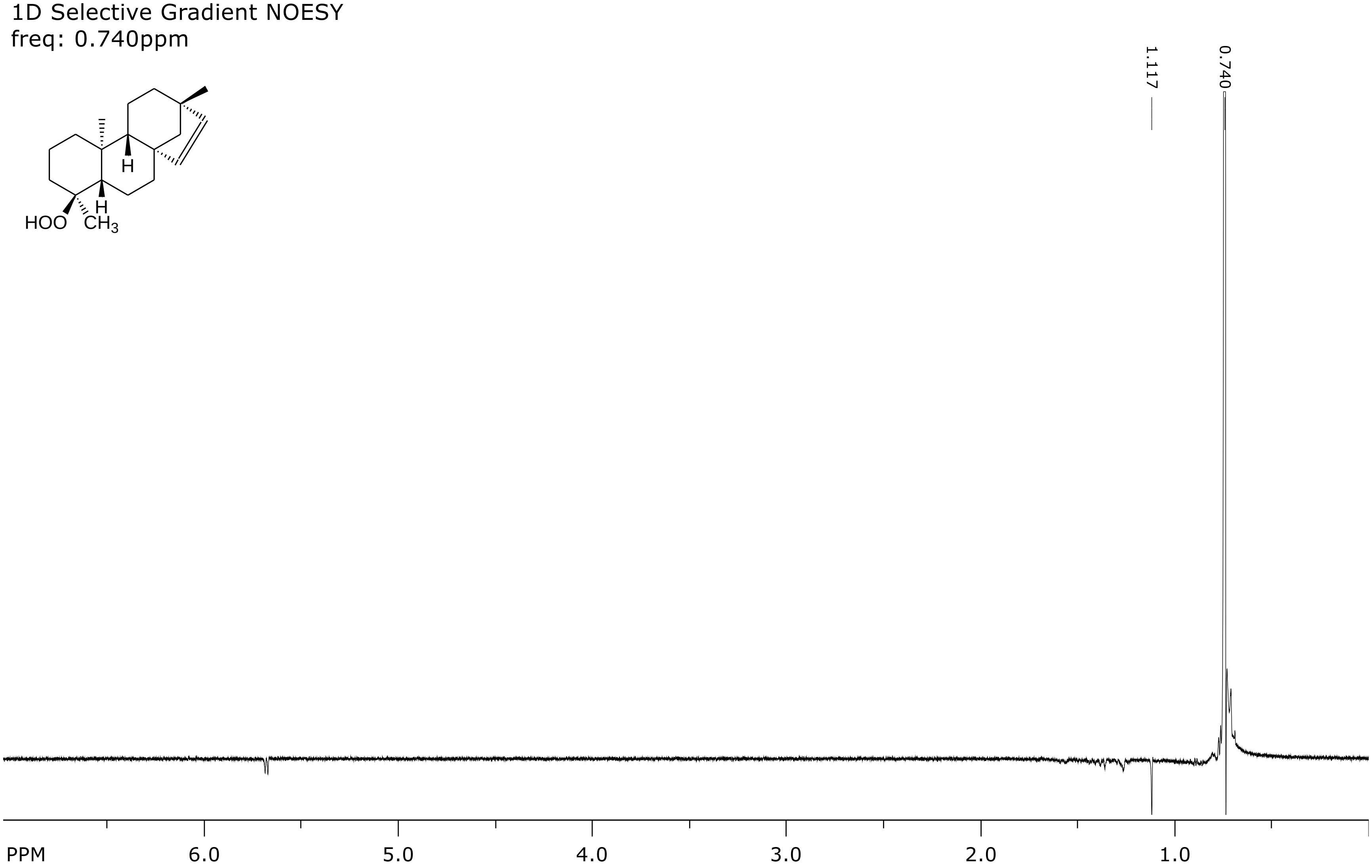

S51. 1D Selective Gradient NOESY spectrum (400 MHz) of **8** in $CDCl_3$; Irradiation at δ 1.117

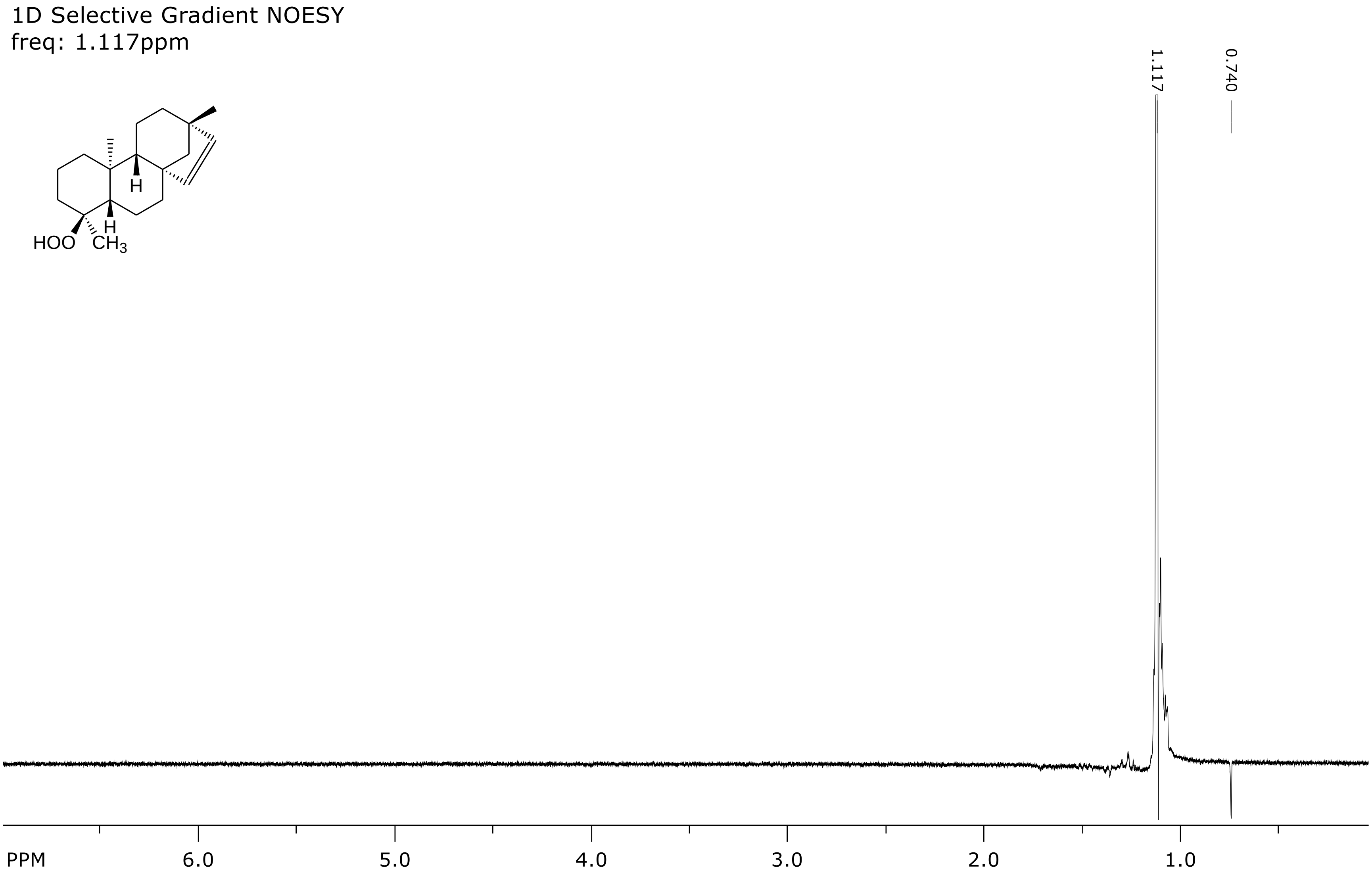

S52. ^{1}H NMR Spectrum (400 MHz) of **9** in $CDCl_3$

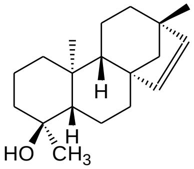

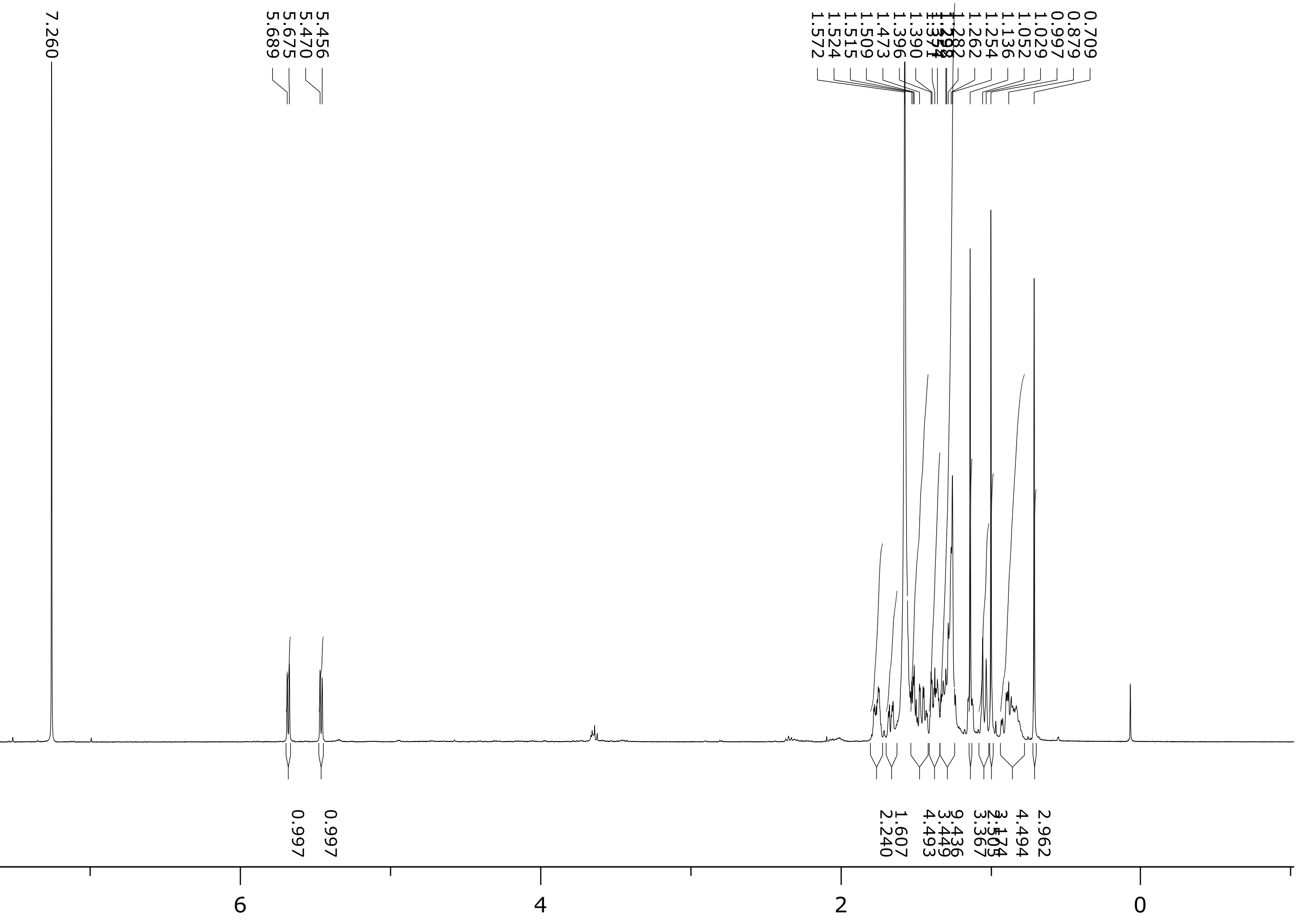

S53. ^{13}C NMR Spectrum (100 MHz) of **9** in $CDCl_3$

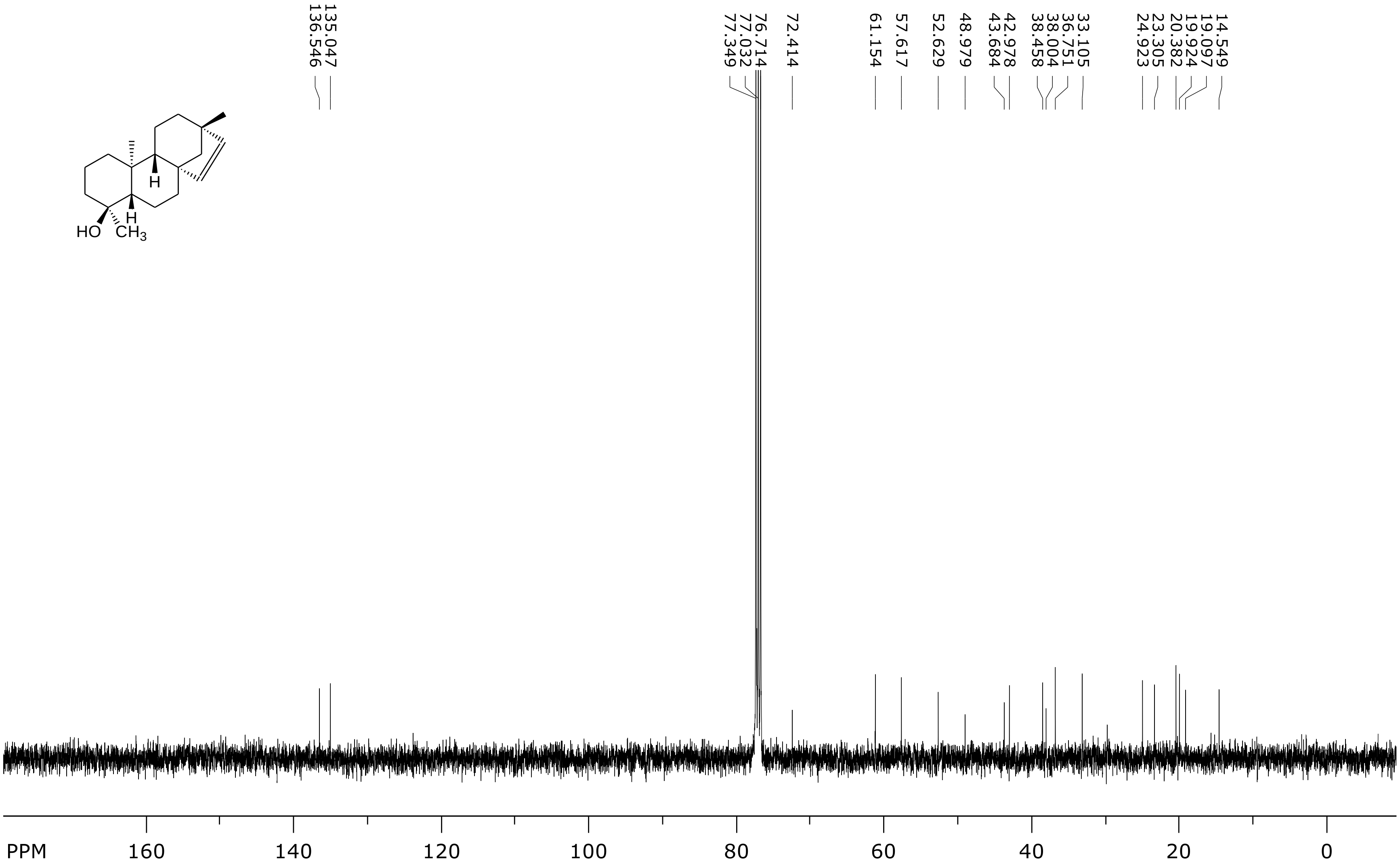

S54. DEPT135 Spectrum (100 MHz) of **9** in $CDCl_3$

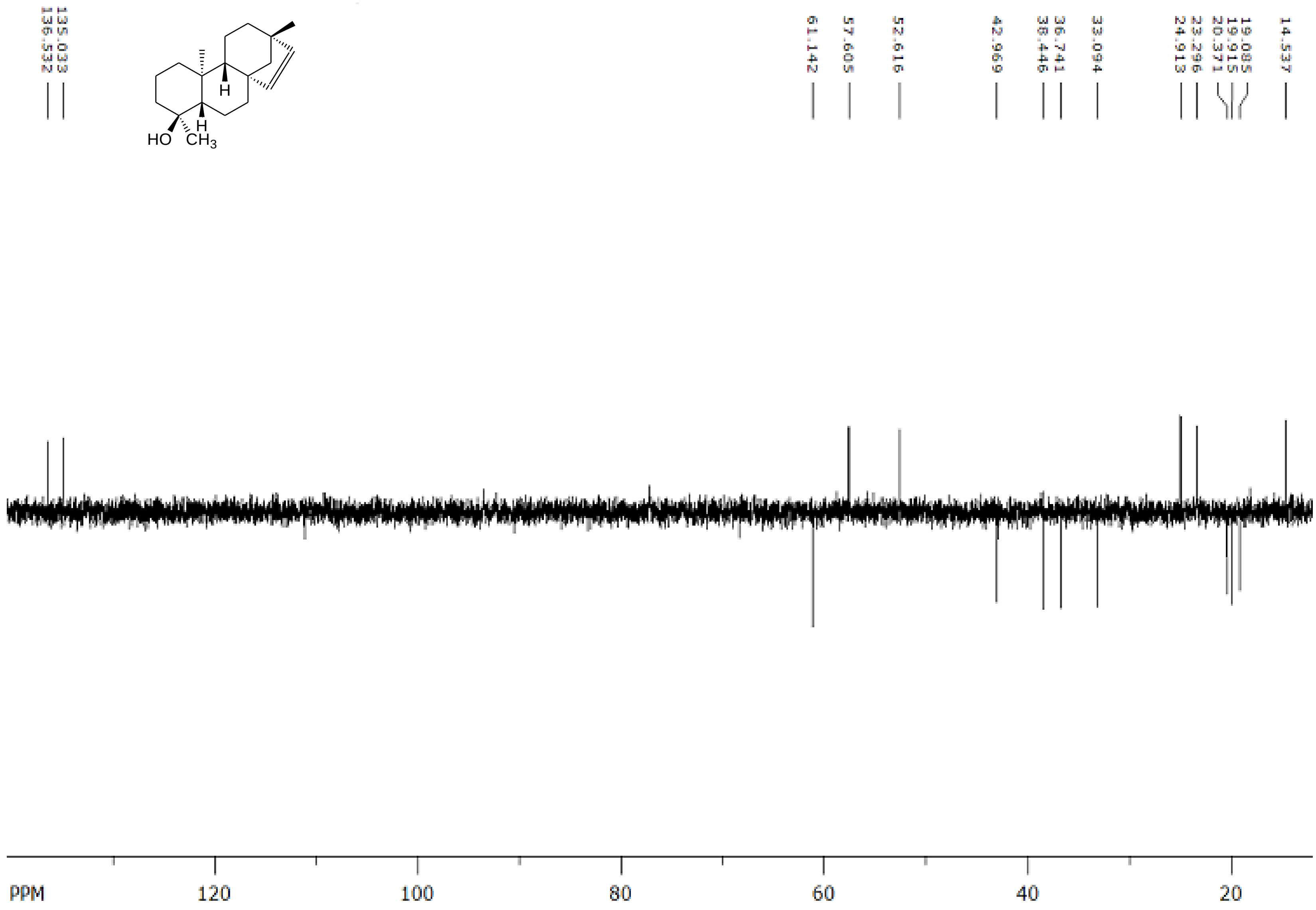

S55. HSQC Spectrum of **9** in $CDCl_3$

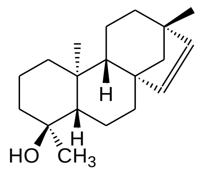

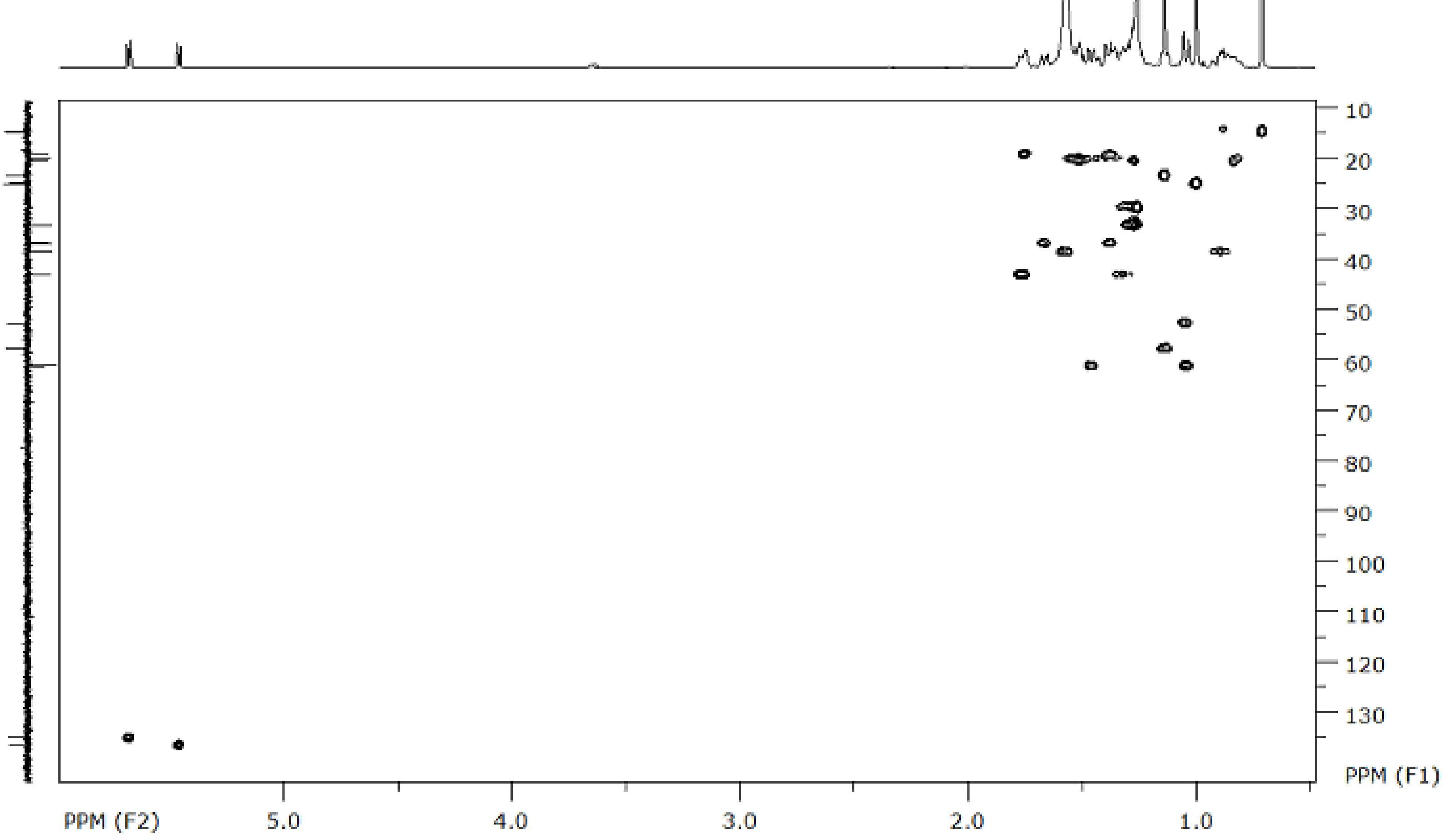

S56. HMBC Spectrum of **9** in $CDCl_3$

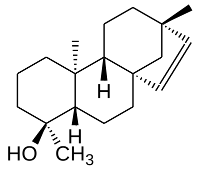

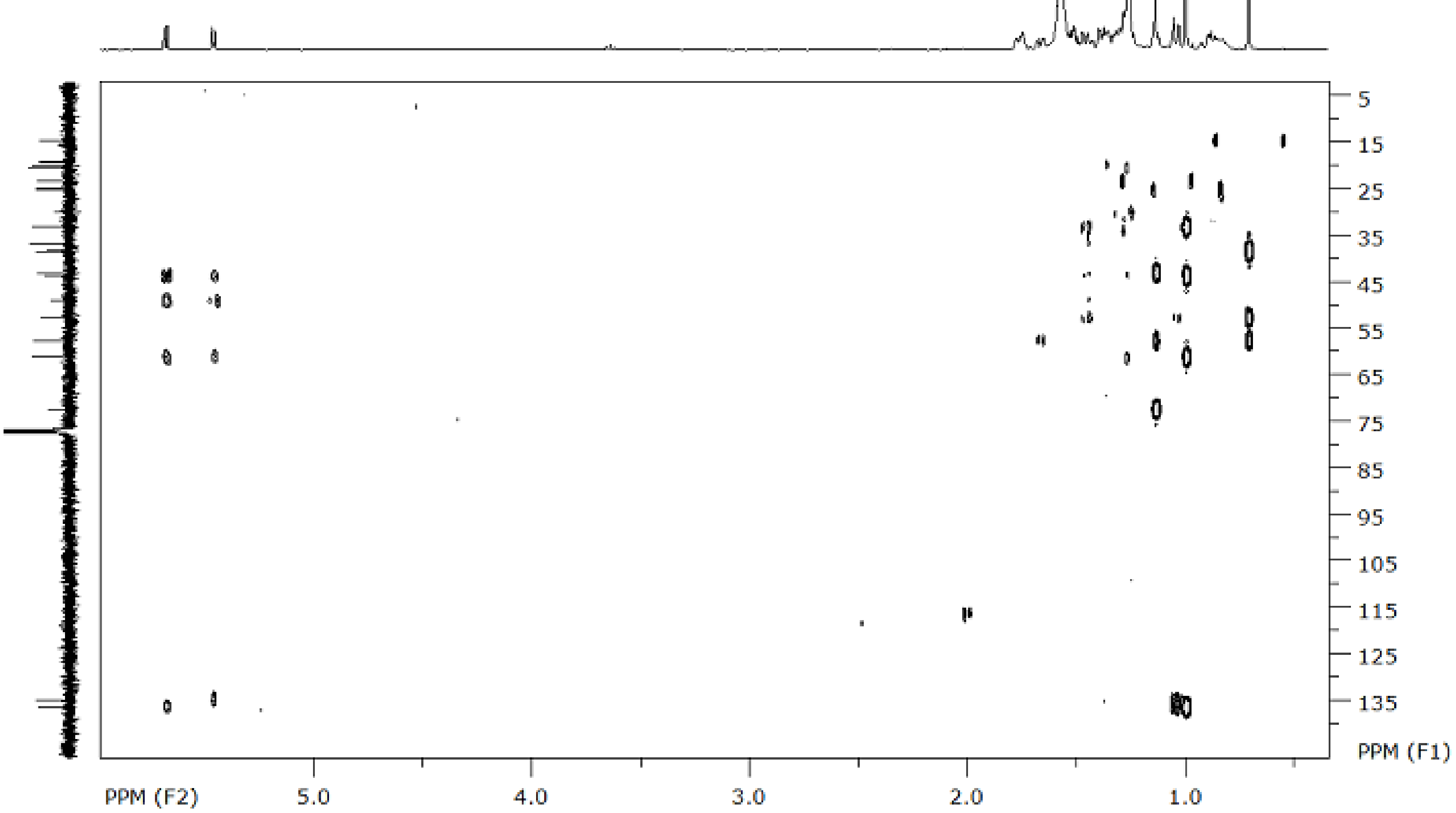

S57. 1D Selective Gradient NOESY spectrum (400 MHz) of **9** in $CDCl_3$; Irradiation at δ 0.708

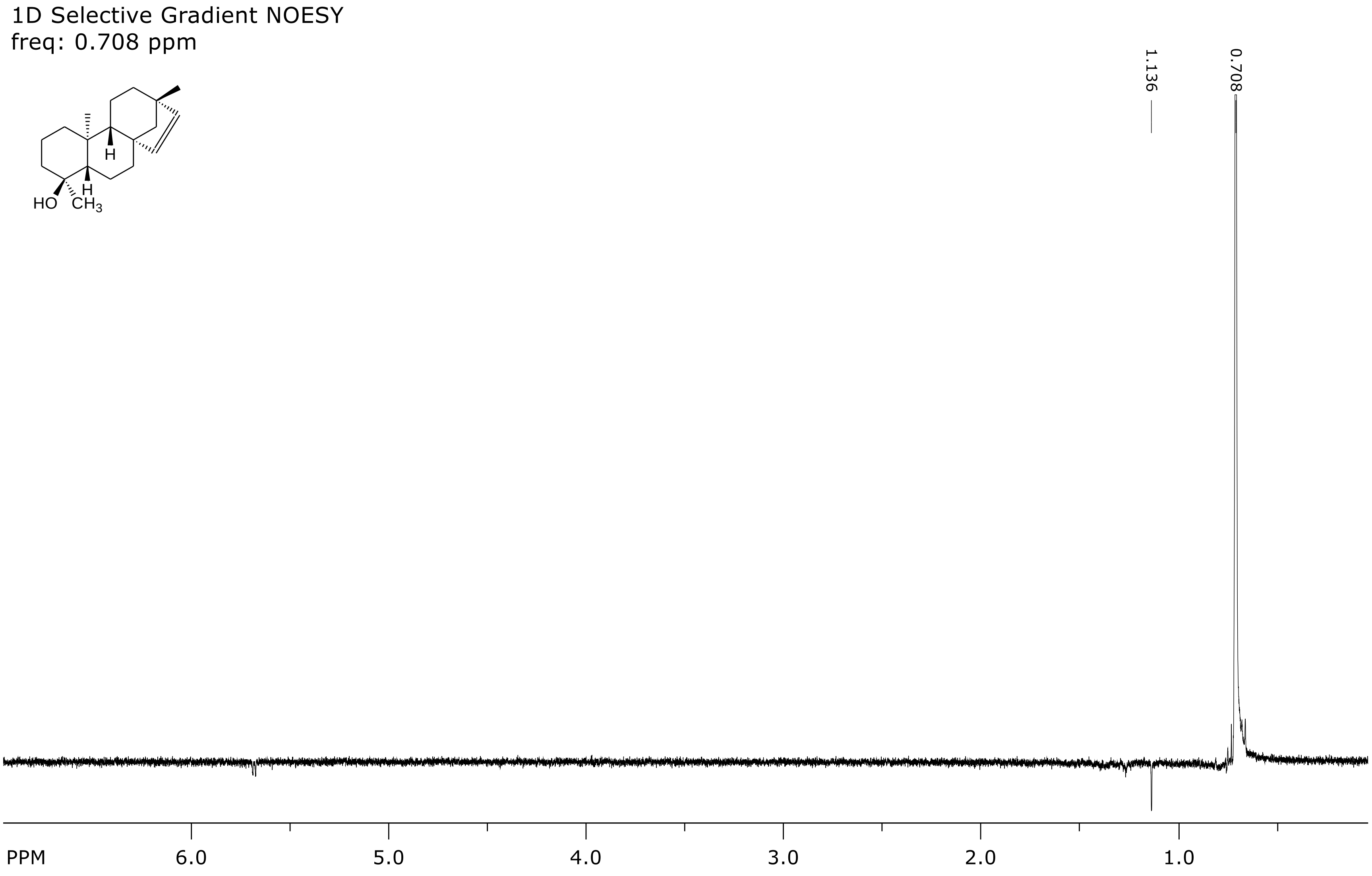

S58. 1D Selective Gradient NOESY spectrum (400 MHz) of **9** in $CDCl_3$; Irradiation at δ 1.135

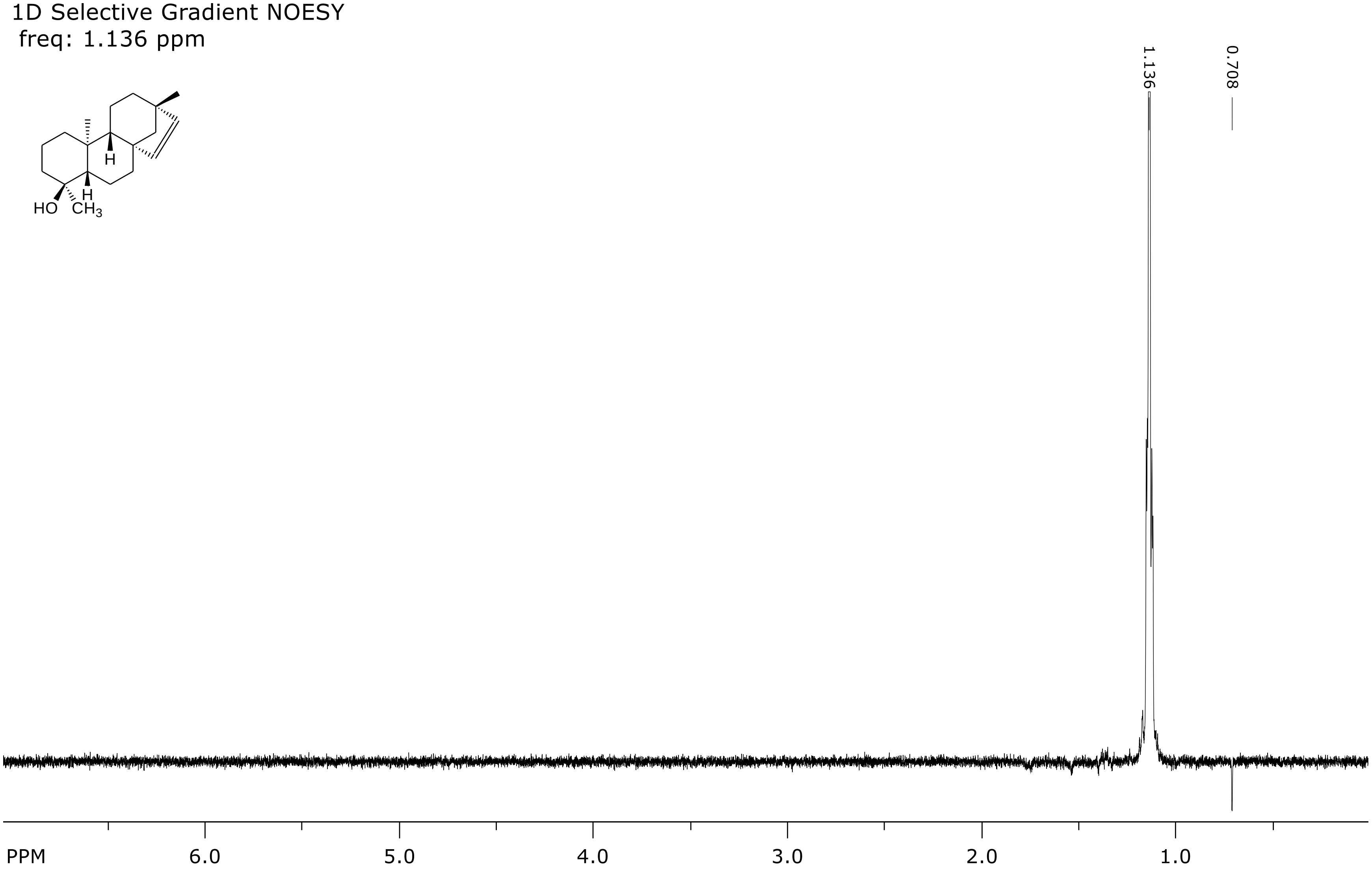

Supplement: Supplementary file 2 — Additional file 2: Table S1. Reported and observed 13C NMR data of ent-beyer-15-ene (1) and erythroxylol A (2); Tables S2 and S3. Assignments of spectroscopic data and selected HMBCs of compounds 5 and 9 respectively; Figures S4–S58.1H NMR, 13C NMR, DEPT135, HSQC, and HMBC spectra of compounds 4–9 and 1D Selective NOESY Gradient spectra of compounds 4, 6a, 6b, 7–9. [file 13065_2020_671_MOESM2_ESM.pdf]
